# Integrating Bidirectional Mendelian Randomization with Multi-Omics Reveals Causal Serum Metabolites and Novel Metabolic Drivers of Multiple Myeloma

**DOI:** 10.3390/ijms27041904

**Published:** 2026-02-16

**Authors:** Yuanheng Liu, Daoyuan Qin, Haohan Ye, Lujun Tang, Xiaoli Li

**Affiliations:** 1 School of Basic Medical Sciences, Chongqing Medical University, Chongqing 400016, China; 2 Chongqing College of Traditional Chinese Medicine, Chongqing 402760, China; 3 Laboratory of Developmental Biology, Department of Genetics and Cell Biology, School of Basic Medical Sciences, Chongqing Medical University, Chongqing 400016, China

**Keywords:** serum metabolites, multiple myeloma, mendelian randomization, multi-omics, causal mechanisms

## Abstract

Multiple myeloma (MM) is a clonal plasma cell neoplasm characterized by autonomous immunoglobulin overproduction. Despite associations between serum metabolites and MM, causal mechanisms remain unclear. Here, we employed bidirectional Mendelian randomization (MR) using 452 serum metabolites to elucidate causal associations with MM risk. The inverse variance-weighted (IVW) method was prioritized, complemented by MR-Egger and weighted median (WM) analyses to address horizontal pleiotropy. Sensitivity analyses—including Cochran’s Q test, MR-Egger intercept evaluation, and leave-one-out (LOO) robustness checks—confirmed result stability. Pathway enrichment was performed using MetaboAnalyst 6.0. RNA-seq data were integrated to identify transcriptional regulators and signaling pathways mediating serum metabolite-driven MM. Among 21 metabolites significantly associated with MM, 8 exhibited protective inverse correlations, while 13 showed risk-enhancing effects. Sensitivity analyses further confirmed the validity of the observed relationships, while bidirectional MR confirmed no reverse causality. Pathway enrichment highlighted valine/leucine/isoleucine biosynthesis and biotin metabolism as pivotal pathways. Integrating transcriptomic data revealed 11 overlapping genes enriched in metal ion transmembrane transporter activity and glycosaminoglycan biosynthesis—chondroitin sulfate/dermatan sulfate. This study established a causal relationship between specific serum metabolites and MM and revealed that key genes may affect the development of MM through metabolic-epigenetic crosstalk, providing preliminary insights into potential therapeutic targets.

## 1. Introduction

Multiple myeloma, a clonal plasma cell malignancy, represents 1% of all hematologic malignancies and ranks as the second most prevalent after lymphoproliferative disorders. The worldwide prevalence of MM has surged by 126%, driven by demographic expansion, population ageing, and rising age-specific incidence trends [1,2]. Current therapeutic strategies for MM encompass proteasome inhibitor-based regimens (e.g., bortezomib), oral immunomodulatory agents, anti-CD38 monoclonal antibodies, and autologous hematopoietic stem cell transplantation (ASCT) in eligible patients [1,3]. While these approaches have significantly improved median survival durations, MM remains incurable due to clonal heterogeneity and drug resistance mechanisms. This therapeutic impasse underscores the imperative to elucidate the genetic, epigenetic, and bone marrow microenvironmental drivers underlying MM pathogenesis and therapeutic resistance. Robust identification of pathogenic risk determinants in MM could catalyze the development of targeted prevention frameworks and personalized therapeutic interventions, thereby mitigating the global disease burden through early risk stratification and modifiable determinant mitigation.

According to reports, the carcinogenesis of MM and its precursor states is driven by a complex interplay of modifiable and non-modifiable factors, encompassing adiposity-related adipokine dysregulation [4,5], dietary patterns [6,7], vitamin D deficiency [8,9], innate immune suppression [10], and genotoxic stressors (ionizing radiation, benzene derivatives) [11,12]. Emerging evidence positions serum metabolomic signatures as multidimensional biomarkers reflecting both germline risk alleles and habitat-specific exogenous stressors, providing new insights for the progress of MM. A study identified 54 significantly altered metabolites via metabolomic analysis, with six exhibiting utility as discriminatory biomarkers to distinguish MM patients from healthy controls [13]. Another research revealed 70 metabolites with marked changes in newly diagnosed MM patients, including diagnostically relevant metabolites such as lactic acid and leucine [14]. However, the precise causal relationships between distinct serum metabolomic signatures and MM initiation/progression remain incompletely elucidated.

While metabolomic profiling has identified dysregulated pathways (e.g., lipid metabolism, amino acid cycling) in MM, these studies face three critical limitations: (1) Observational metabolomic associations are confounded by lifestyle, comorbidities, and reverse causality (e.g., disease progression altering metabolite levels). (2) Most metabolomic studies enroll < 100 cases, limiting the power to detect modest effect sizes. (3) Traditional statistical methods cannot disentangle metabolic drivers from downstream epiphenomena. MR circumvents these issues by leveraging germline genetic variants as instrumental variables (IVs) to infer causal relationships. By leveraging the random assortment of alleles during meiosis, MR circumvents confounding by unmeasured factors and mitigates reverse causality, as genetic variants are fixed at conception and temporally precede disease onset [15,16]. To systematically dissect the causal cascade linking serum metabolites to MM pathogenesis, we employed a multi-omics framework integrating MR, transcriptomic, and metabolomic profiling, an approach guided by two key hypotheses positing that serum metabolites exhibit causal effects on MM risk distinct from correlative associations and that these metabolites modulate MM progression through specific biological pathways. To test these, we first conducted bidirectional two-sample MR analysis. Next, metabolic pathway enrichment analysis prioritized pathways enriched with MR-identified causal metabolites to provide biological context. Third, we mapped IVs from significant metabolites to their corresponding genes and overlapped these with differentially expressed genes (DEGs) from MM transcriptomic datasets, followed by functional enrichment of overlapping genes to reveal mechanistic links. Specifically, this integrated strategy aimed to unravel the mechanistic underpinnings of metabolite-driven MM progression and identify modifiable metabolic nodes for precision therapy.

## 2. Results

### 2.1. Strength of the Instrumental Variables

For the forward MR analysis, a total of 8471 independent SNPs associated with 452 serum metabolites were initially extracted as candidate instrumental variables. Following harmonization with the outcome GWAS (MM) summary statistics, 7654 SNPs remained for the final MR analysis. The mean F-statistic for these instrumental variables was 26.68 (range: 17.64 to 2913.7), and this broad range in F-values, together with the varying SNP counts across metabolites, reflects the inherent differences in their genetic architecture and heritability. For the reverse MR analysis, a total of 33 independent SNPs associated with MM were selected as instrumental variables. The mean F-statistic for these reverse MR instrumental variables was 21.74 (range: 19.53 to 25.74). All F-values for instrumental variables in both analyses exceeded 10, confirming sufficient IV strength and no risk of weak instrument bias.

### 2.2. Mendelian Randomization Analysis Results

**Figure 1:**
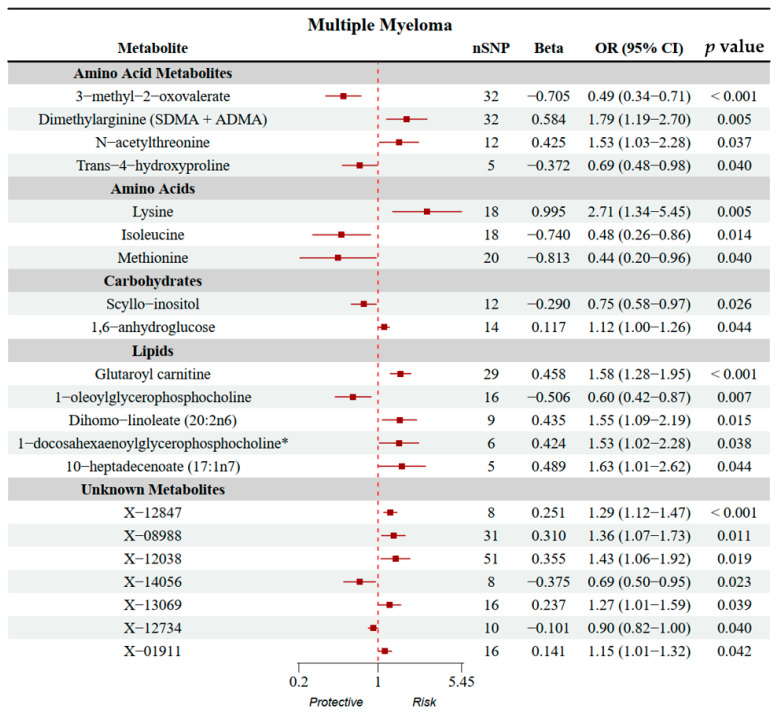
Forest plots showing MR analysis of serum metabolites and their association with MM risk. (Note: For visualization purposes in the forest plots, Beta and Standard Error (SE) values were scaled. The Odds Ratios (ORs) presented in the text and Table 1 represent the unscaled, true effect sizes.) * Indicates metabolites for which reference spectra of the pure substances were not directly measured on the Metabolon platform.

In the forward MR analysis, the Inverse Variance-Weighted (IVW) method identified 21 significant serum metabolites with suggestive causal effects at nominal significance (unadjusted *p*-value < 0.05) on the risk of MM as part of an exploratory analysis to prioritize potential candidate metabolites (note that this nominal threshold does not account for multiple testing and may be subject to false positive findings). Among these, 13 metabolites were associated with an increased risk of MM (odds ratio: OR > 1), while 8 metabolites were associated with a decreased risk (OR < 1). The metabolite showing the strongest positive association was glutaroyl carnitine (OR: 1.00, 95% CI: 1.00–1.01, *p* < 0.001), while the metabolite showing the strongest protective effect was 3-methyl-2-oxovalerate (OR: 0.99, 95% CI: 0.99–1.00, *p* < 0.001). Other identified risk-associated metabolites (OR > 1) included the unknown metabolite X-12847 (OR = 1.00, 95% CI: 1.00–1.00, *p* < 0.001), dimethylarginine (SDMA + ADMA) (OR = 1.01, 95% CI: 1.00–1.01, *p* = 0.005), lysine (OR = 1.01, 95% CI: 1.00–1.02, *p* = 0.005), 10-heptadecenoate (17:1n7) (OR = 1.00, 95% CI: 1.00–1.01, *p* = 0.044), 1-docosahexaenoylglycerophosphocholine (OR = 1.00, 95% CI: 1.00–1.01, *p* = 0.039), N-acetylthreonine (OR = 1.00, 95% CI: 1.00–1.01, *p* = 0.037), dihomo-linoleate (20:2n6) (OR = 1.00, 95% CI: 1.00–1.01, *p* = 0.015), and the unknown metabolites X-12038 (OR = 1.00, 95% CI: 1.00–1.01, *p* = 0.019), X-08988 (OR = 1.00, 95% CI: 1.00–1.01, *p* = 0.011), X-13069 (OR = 1.00, 95% CI: 1.00–1.01, *p* = 0.039), X-01911 (OR = 1.00, 95% CI: 1.00–1.00, *p* = 0.042), and 1,6-anhydroglucose (OR = 1.00, 95% CI: 1.00–1.00, *p* = 0.044). Conversely, additional protective metabolites (OR < 1) were identified as 1-oleoylglycerophosphocholine (OR = 0.99, 95% CI: 0.99–1.00, *p* = 0.007), isoleucine (OR = 0.99, 95% CI: 0.99–1.00, *p* = 0.014), methionine (OR = 0.99, 95% CI: 0.98–1.00, *p* = 0.040), trans-4-hydroxyproline (OR = 1.00, 95% CI: 0.99–1.00, *p* = 0.040), scyllo-inositol (OR = 1.00, 95% CI: 0.99–1.00, *p* = 0.026), and the unknown metabolites X-14056 (OR = 1.00, 95% CI: 0.99–1.00, *p* = 0.023) and X-12734 (OR = 1.00, 95% CI: 1.00–1.00, *p* = 0.040). These findings provide robust evidence for the causal role of systemic metabolic alterations in the development of MM. These results are presented in Figure 1, for which the SE and Beta coefficients were uniformly scaled by a factor of 100 solely to enhance the visual clarity of the confidence intervals. This graphical adjustment does not affect the statistical significance or direction of the reported associations. All numerical results and inferences are based on the original, unscaled data. Complete results are provided in Table 1. While 22 metabolites showed nominal significance (*p* < 0.05), these associations should be considered suggestive due to the limited power of the outcome GWAS. Replication in larger, well-powered cohorts is essential to confirm their validity.

### 2.3. Sensitivity and Reverse Causality Analysis Results

**Figure 2:**
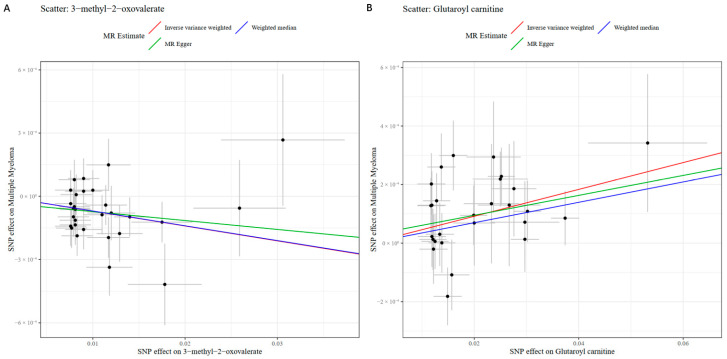

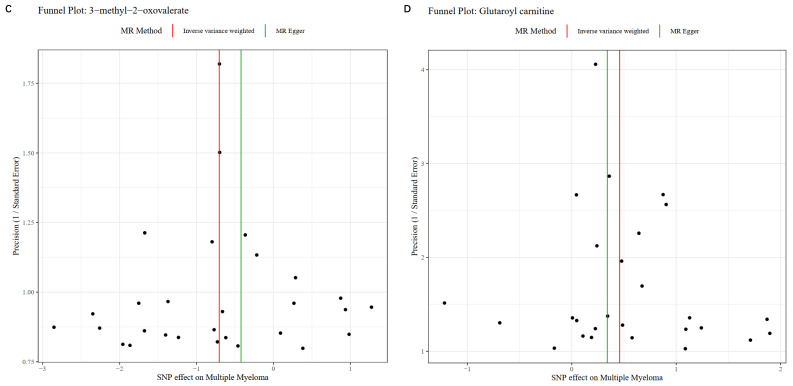

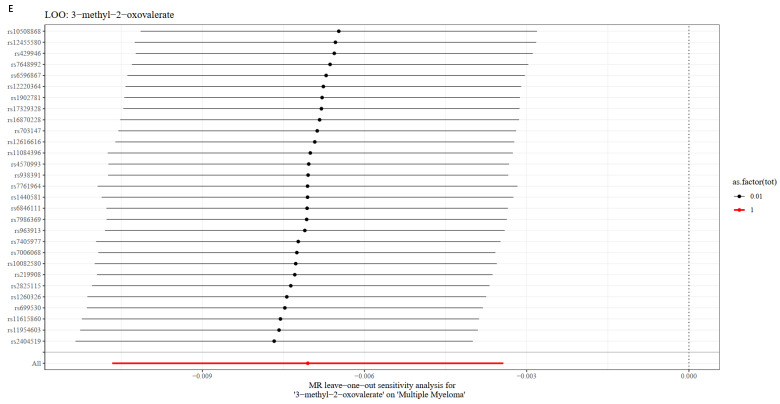

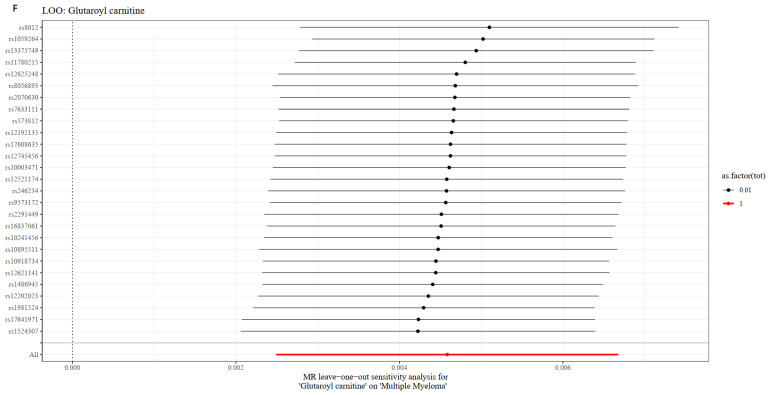
Visualization of MM estimates and sensitivity analyses for the two most significant causal metabolites (3-methyl-2-oxovalerate and Glutaroyl carnitine). (**A**) Scatter plot of the genetic association between 3-methyl-2-oxovalerate and MM risk. (**B**) Scatter plot of the genetic association between glutaroyl carnitine and MM risk. (**C**) Funnel Plot of the genetic association between 3-methyl-2-oxovalerate and MM risk. (**D**) Funnel Plot of the genetic association between glutaroyl carnitine and MM risk. (**E**) LOO plot of the genetic association between 3-methyl-2-oxovalerate and MM risk. (**F**) LOO plot of the genetic association between glutaroyl carnitine and MM risk.

For the forward MR analysis, sensitivity tests were conducted on the 21 significant metabolites. Cochran’s Q test using both IVW and MR-Egger methods showed no significant heterogeneity among instrumental variables (IVW *p*-values: 0.2023–0.9949; MR-Egger *p*-values: 0.2035–0.9872). The MR-Egger intercept test revealed no significant horizontal pleiotropy for 20 metabolites (*p*-values: 0.2578–0.9053). Only Lysine showed a potential weak pleiotropic signal (intercept = 0.0004, *p*-value = 0.0312), indicating that this metabolite should be interpreted with caution as the weak pleiotropy signal introduces minor uncertainty to its causal inference. Visualization of sensitivity analyses for the two most significantly causal metabolites (glutaroyl carnitine and 3-methyl-2-oxovalerate), which showed consistent results across all sensitivity assessments, is presented in Figure 2. Corresponding sensitivity analysis visualizations for the remaining 19 significantly causal metabolites are provided in Figures S1–S3. These sensitivity results are presented in Table 1. For the reverse MR analysis, the Steiger directionality test rejected the reverse causal direction for 18 metabolites (Steiger *p*-value < 0.05, Correct Direction = FALSE). For three metabolites (Isoleucine, 3-methyl-2-oxovalerate, and 10-heptadecenoate [17:1n7]), Steiger *p*-values were non-significant (0.1125, 0.1086, and 0.0679), though direction labels remained FALSE. A complex pattern emerged for Dimethylarginine (SDMA + ADMA) and glutaroyl carnitine: IVW analysis showed nominally significant reverse associations (*p*-value = 0.0163 and 0.0276), but Steiger tests strongly rejected the reverse direction (*p*-value = 0.0011 and 0.0021). For the remaining metabolites, reverse IVW results were non-significant (*p*-value > 0.05) and Steiger tests rejected reverse causality. While glutaroyl carnitine and dimethylarginine (SDMA + ADMA) are robust candidate biomarkers for MM risk based on forward MR findings, their discordant reverse MR and Steiger results warrant cautious interpretation of causal directionality for these metabolites. Complete reverse MR results are presented in Table S1.

### 2.4. Results of Metabolic Pathway Analyses

**Figure 3:**
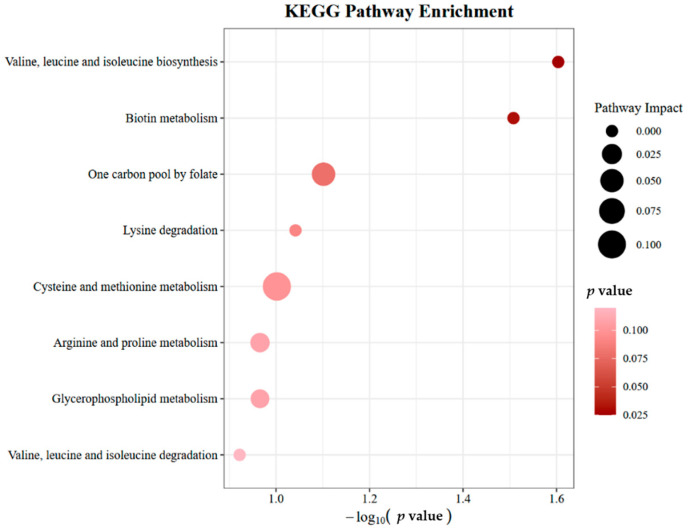
KEGG pathway enrichment analysis based on the causal metabolites identified via MR.

To elucidate the biological context of the metabolites implicated by MR analysis, we performed a metabolic pathway enrichment analysis. This analysis of the 21 significant causal metabolites identified 8 relevant KEGG pathways. It is important to explicitly acknowledge that these pathway enrichment results are based on nominal significance (unadjusted *p* < 0.05) and borderline *p*-values, without false discovery rate (FDR) correction or other pathway-level multiple testing adjustment, which limits the strength of the biological conclusions drawn from these findings. Two pathways reached nominal significance (Valine, leucine and isoleucine biosynthesis, *p*-value = 0.0249; Biotin metabolism, *p*-value = 0.0311). Six additional pathways showed borderline significance (*p*-values: 0.0791–0.1196). Pathway impact analysis revealed that cysteine and methionine metabolism had the highest impact value (0.1045), followed by one-carbon pool by folate (0.0508); other pathways showed minimal or no impact (0.0000–0.0209). These findings should be interpreted as providing a suggestive biological context for the observed metabolite-MM associations, rather than definitive mechanistic proof. These results are presented in Figure 3 and Table S2.

### 2.5. Mapping SNPs to Genes and Identification of DEGs in MM

**Figure 4:**
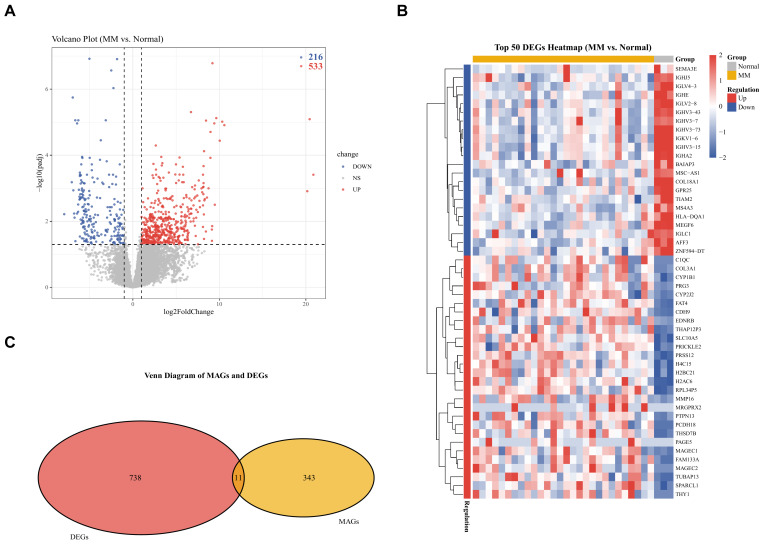
Identification of DEGs in the transcriptome and their association with MAGs in MM. (**A**) Volcanic diagram of DEGs between MM and Normal groups. (**B**) Heatmap of expression profiles of the top 50 DEGs in MM and Normal groups. (**C**) Venn diagram of the intersection between 749 MAGs and 533 DEGs.

To link genetic variants to gene expression, the 404 significant instrumental variables associated with the 21 significantly causal metabolites were mapped to genes, yielding a set of 354 unique metabolite-associated genes (MAGs). To clarify the prioritization and filtering strategy for these MAGs prior to integration with transcriptomic data, we prioritized genes based on their proximity to the mapped SNPs, and no arbitrary filtering was applied to the MAGs list before its intersection with transcriptomic data. This approach was adopted to maximize the sensitivity of identifying overlaps between MAGs and DEGs. Parallelly, differential expression analysis of the GSE153380 dataset (MM patients vs. healthy donors) identified 749 DEGs. Among these, 533 genes were up-regulated and 216 were down-regulated in MM. The overall distribution of these DEGs is visualized in Figure 4A, and the top 50 most significantly dysregulated genes are presented in Figure 4B.

### 2.6. Identification and Pathway Enrichment Analysis of Overlapping Genes

**Figure 5:**
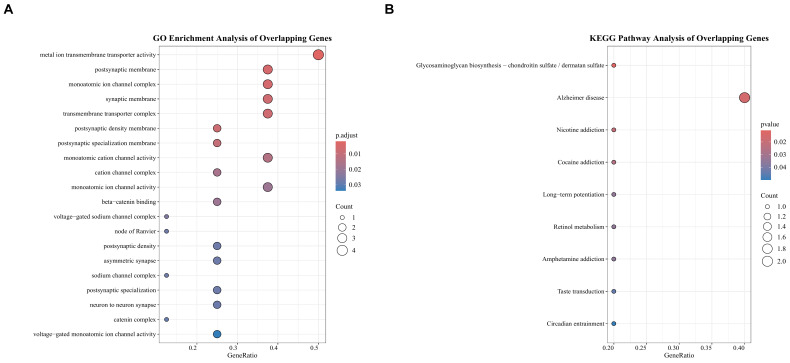
Functional annotation and pathway enrichment analysis for the 11 candidate genes identified at the intersection of MAGs and DEGs in MM. (**A**) GO enrichment for overlapping genes. (**B**) KEGG pathway enrichment for overlapping genes.

To identify potential key mediator genes, we intersected the MAGs with the DEGs. This yielded 11 overlapping genes, which are visualized in Figure 4C. While the number of overlapping genes is small, their identification represents a convergence of two independent data layers (genetic association from MAGs and transcriptomic dysregulation from DEGs, which confers high confidence in these 11 genes as candidate mediator targets for further investigation. Functional enrichment analysis of these 11 overlapping genes was subsequently performed as an exploratory analysis to reveal suggestive biological relevance and generate future experimental hypotheses. In the GO pathway analysis, 59 terms were statistically enriched. The top enriched term is metal ion transmembrane transporter activity. The top 20 results of the GO enrichment analysis are presented in Figure 5A. KEGG pathway analysis identified 9 significant pathways, with the most prominent being glycosaminoglycan biosynthesis—chondroitin sulfate/dermatan sulfate (*p*-value = 0.011). All significant KEGG pathways are displayed in Figure 5B.

## 3. Discussion

This MR study identified 21 serum metabolites (8 protective, 13 risk-promoting) significantly associated with MM risk. The strongest risk-enhancing metabolite was glutaroyl carnitine, while 3-methyl-2-oxovalerate exhibited the most robust protective effect. Preliminary pathway analysis (nominal enrichment) suggests that dysregulated valine/leucine/isoleucine (BCAA) biosynthesis and biotin metabolism may represent metabolic vulnerabilities in MM progression. However, these findings are exploratory and require validation with stricter multiple-testing correction in larger cohorts. Multi-omics integration further uncovered 11 hub genes mediating metabolomic-transcriptional crosstalk, which were enriched in metal ion transmembrane transporter activity (e.g., zinc/copper transporters) and glycosaminoglycan (GAG) biosynthesis (e.g., chondroitin sulfate/dermatan sulfate). Our results suggest that metal ion transporters (e.g., zinc/copper transporters) may modulate epigenetic enzyme activity (e.g., DNA methyltransferases), and GAG (e.g., chondroitin sulfate) might influence the bone marrow microenvironment to support tumor survival. However, these links are currently speculative and require experimental validation to establish direct causality. Our findings propose a potential metabolic-epigenetic axis in MM progression, where altered amino acid metabolism, biotin-dependent chromatin remodeling, and the aforementioned pathways collectively contribute to disease mechanisms. This study advances the understanding of MM pathogenesis through integrative metabolomic and genomic analyses, providing a biomarker-guided framework for exploring personalized therapeutic interventions targeting these pathways.

This study demonstrated that glutaroyl carnitine elevation significantly correlates with MM risk. Glutaroyl carnitine is an acylcarnitine compound that is an intermediate in the glutaric acid metabolism pathway and serves as a pivotal diagnostic biomarker for glutaric acidemia type I in clinical medicine. In other diseases, elevated glutaroyl carnitine levels have been positively associated with pro-inflammatory cytokine (e.g., IL-1β) [17] and linked to systemic inflammation (e.g., cardiovascular diseases, age-related decline) [18,19,20]. Elevated glutaroyl carnitine levels characteristic of glutaroyl carnitine type I due to glutaryl-CoA dehydrogenase deficiency are associated with progressive neurodegeneration and systemic inflammation, driven by mitochondrial dysfunction and oxidative stress pathways [21]. Emerging evidence implicates glutaroyl carnitine in the modulation of immune-inflammatory pathways, with recent MR analyses revealing its unique capacity to drive serum C-reactive protein (CRP) elevation post-FDR correction in IVW models, thereby establishing it as a pivotal biomarker in systemic inflammation [22]. While these findings suggest a potential role in inflammation, their relevance to MM pathogenesis remains to be directly tested in myeloma-specific models (e.g., MM cell lines, patient-derived xenografts). Our findings further identify glutaroyl carnitine as a predominant risk determinant in MM, potentially contributing to disease progression via inflammatory pathways that may underlie its prognostic and therapeutic relevance.

Elevated plasma concentrations of 3-methyl-2-oxovalerate demonstrated the most robust protective association with MM progression. 3-Methyl-2-oxovalerate, a BCAA derivative generated during the catabolism of branched-chain keto acid (BCKA), serves as a metabolic intermediate in the valine degradation pathway [23]. 3-Methyl-2-oxovalerate exhibits anticancer activity by disrupting mitochondrial energy metabolism through inhibition of a key tricarboxylic acid (TCA) cycle enzyme. This metabolic disruption shifts energy production toward glycolysis, a phenomenon linked to the Warburg effect, while simultaneously impairing cellular redox balance [24]. BCAA supplementation prevents skeletal muscle wasting in gastric cancer patients by enhancing mTORC1-mediated protein synthesis and suppressing proteasomal degradation, as evidenced by clinical trials showing reduced muscle depletion and improved survival outcomes [25]. The inverse association between 3-methyl-2-oxovalerate and gastric cancer risk observed herein aligns with emerging evidence that 3-methyl-2-oxovalerate may modulate this pathway by disrupting TCA cycle flux, a mechanism distinct from its role in solid tumors. This metabolic modulation may disrupt cancer cell energy homeostasis while enhancing cellular redox capacity, thereby suppressing tumorigenic processes. This discrepancy highlights the need for MM-specific mechanistic studies. For example, 3-methyl-2-oxovalerate’s inhibition of α-ketoacid dehydrogenase complexes—critical in MM energy homeostasis—could suppress myeloma cell survival under hypoxic conditions, though this hypothesis requires experimental validation.

The identification of valine/leucine/isoleucine biosynthesis and biotin metabolism as enriched pathways aligns with MM’s metabolic reprogramming, though these conclusions are based on nominal significance (FDR < 0.05) and a small set of overlapping genes. As highlighted in pathway analysis guidelines, small gene overlaps and nominal significance thresholds require cautious interpretation due to potential biases in gene set overlap and statistical sensitivity. BCAA accumulation in MM aligns with prior studies linking leucine uptake to mTORC1 activation and tumor cell proliferation [26]. Unlike prior studies focusing on BCAA catabolism, our results suggest that BCAA may serve as a key nutrient source for MM cell proliferation, potentially reflecting metabolic reprogramming to sustain rapid growth under hypoxic bone marrow conditions [27,28]. Biotin is an essential coenzyme for histone acetyltransferases (HATs), and histone acetylation is a key event in epigenetic activation. In MM cells, dysregulation of biotin metabolism can lead to abnormal histone acetylation, which in turn activates the expression of oncogenes such as c-MYC [29,30]. Metal ions (such as iron and zinc) are essential cofactors for epigenetic enzymes (e.g., DNA methyltransferase requires S-adenosylmethionine (SAM) as a methyl donor, and SAM synthesis depends on metal ions). The enrichment of metal ion transmembrane transporter activity in our data suggests that dysregulation of metal ion transport may correlate with altered epigenetic enzyme activity (e.g., DNA methyltransferases), which has been implicated in abnormal gene expression in MM [31]. Glycosaminoglycans (such as chondroitin sulfate) are important components of the extracellular matrix (ECM), and disorders in glycosaminoglycan synthesis may alter the MM microenvironment (e.g., extracellular matrix composition), which has been linked to epigenetic regulation via cytokine signaling pathways [32]. While these associations are intriguing, they remain hypothesis-generating. Future studies should employ MM-specific models (e.g., glutaroyl carnitine-exposed RPMI8226 cells) to determine whether BCAA metabolism directly drives MM progression via NF-κB or mTORC1 pathways. Additionally, integrating metabolomic and transcriptomic data with single-cell resolution could resolve pathway-specific contributions to MM heterogeneity.

While this study advances our understanding of metabolite-MM causal relationships and underlying mechanisms, critical limitations warrant consideration. First, the validity of MR inferences relies on three core assumptions (relevance, independence, exclusion restriction). While we tested for weak instruments (F-statistic > 10) and horizontal pleiotropy, residual pleiotropy (e.g., unmeasured confounding) may still bias estimates. Additionally, the limited sample size of the outcome GWAS reduces statistical power to detect weak metabolite-MM associations, increasing the risk of false negatives. Thus, the identified “significant” associations should be considered suggestive and require replication in independent cohorts. Second, while two-sample MR provides evidence for serum metabolite-MM pathogenesis associations, mechanistic validation through orthogonal experimental frameworks remains essential. Cellular models (e.g., metabolite-exposed MM cell lines) and genetically engineered murine models (e.g., Mmset-haploinsufficient mice with targeted metabolite modulation) are indispensable for systematically addressing confounding variables and elucidating whether metabolic dysregulation represents a primary pathogenic driver or secondary downstream consequence of MM progression. Third, the outcome GWAS included only 601 cases, resulting in limited statistical power to detect modest genetic effects. This increases the risk of false negatives and suggests that our identified metabolite-MM associations should be interpreted as preliminary evidence. Larger cohorts (e.g., *n* > 1000 cases) are needed to validate these findings and improve estimate stability. Finally, while emerging evidence implicates dysregulated BCAA metabolism and epigenetic remodeling in MM pathogenesis, experimental validation of their pathogenic mechanisms remains fragmented. Systematic elucidation of these pathways demands advanced functional approaches, including CRISPR-Cas9-mediated gene disruption of metabolic regulators and single-cell RNA sequencing to resolve metabolite-induced cellular reprogramming dynamics [33,34], particularly in bone marrow niche microenvironments. Future investigations must systematically resolve these constraints to elucidate MM’s biological underpinnings, thereby informing precision therapeutic frameworks.

## 4. Materials and Methods

### 4.1. Study Design

**Figure 6:**
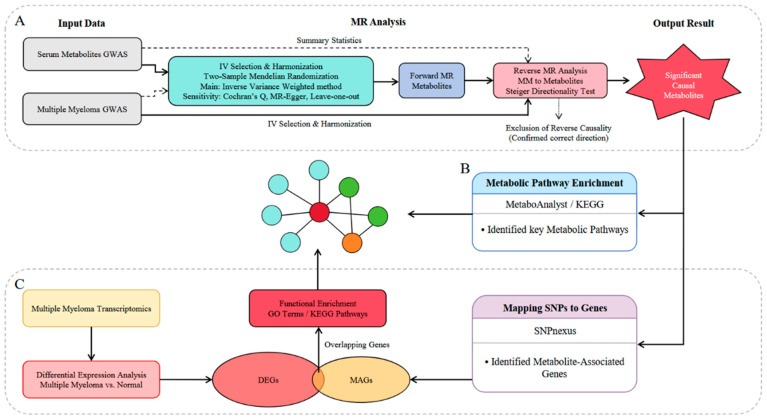
Study design and analytical workflow. (**A**) MR Analysis. Bidirectional two-sample MR was performed to infer causal associations between serum metabolites and MM. The IVW method was prioritized, complemented by MR-Egger and WM analyses to address horizontal pleiotropy. Sensitivity analyses—including Cochran’s Q test, MR-Egger intercept evaluation, and LOO robustness checks—confirmed result stability. Steiger directionality testing excluded reverse causality. (**B**) Causal metabolites identified by MR were analyzed using MetaboAnalyst 6.0 for pathway enrichment. (**C**) Multi-omics Integration. Transcriptomic data from MM patients were integrated with metabolomic data. Overlapping genes between DEGs and MAGs were identified, followed by functional annotation to explore signaling pathways mediating serum metabolite-driven MM. *MR*, Mendelian randomization. *MM*, multiple myeloma. *IVW*, inverse variance-weighted. *WM*, weighted median. *LOO*, leave-one-out. *DEGs*, Differentially Expressed Genes. *MAGs*, metabolite-associated genes.

A multi-step study design was employed to explore the causal association between serum metabolites and MM and investigate potential biological mechanisms (Figure 6). First, a bidirectional two-sample MR analysis was conducted to assess the causal effects of serum metabolites on MM risk. Causal effects were estimated using dual-sample MR with three complementary analytical strategies: inverse variance-weighted (IVW), MR-Egger regression, and weighted median estimators. Robustness assessments included Cochran’s Q statistic, MR-Egger, and LOO validation. Second, metabolic pathway analysis was performed to identify biological pathways enriched by the identified causal metabolites. Third, to bridge the gap between genetic variants and gene expression, we mapped the IVs of significant metabolites to their corresponding genes. Finally, we utilized transcriptome sequencing data to identify DEGs in MM patients compared to healthy controls. The overlapping genes between MAGs and DEGs were subjected to functional enrichment analyses to uncover potential molecular mechanisms.

### 4.2. Data Source

Summary statistics for 452 serum metabolites were obtained from a large-scale genome-wide association study (GWAS) available in the IEU OpenGWAS project (dataset IDs: met-a-303 to met-a-754). The original GWAS identified metabolite-associated loci in 7824 individuals of European descent [35]. Genetic associations for MM were derived from the summary statistics of a European genome-wide association study (GWAS ID: ieu-b-4957, 2021) comprising 601 cases and 372,016 controls; this relatively small number of MM cases limits the statistical power to detect weaker metabolite-MM genetic associations. For transcriptomic analysis, the raw RNA-seq counts data of the dataset GSE153380 (released in 2020) were downloaded from the Gene Expression Omnibus (GEO) database. This dataset, derived from a European cohort, comprises the following samples: 5 primary plasma cell (PC) samples from healthy donors, 28 MM plasma cell samples from patients, and 5 MM cell line samples. Detailed sample information was extracted from the series matrix file to ensure correct grouping. Differential expression analysis was performed using DESeq2, which internally accounts for technical covariates including library size differences via size factor normalization; a design formula of ~ Group was applied to directly assess gene expression differences between MM and healthy control samples, with this analytical approach mitigating potential batch effects and sample heterogeneity related to technical sequencing variation.

### 4.3. Selection of Instrumental Variables

To select valid IVs for the forward MR analysis, we applied a significance threshold of *p* < 1 × 10^−5^. This relaxed threshold (rather than the conventional genome-wide significance of 5 × 10^−8^) was adopted for metabolomic MR analyses because metabolites represent intermediate phenotypes that typically have smaller GWAS sample sizes compared to major complex diseases; use of the more stringent genome-wide threshold would yield an insufficient number of SNPs to support robust sensitivity analyses (e.g., MR-Egger). This approach is a standard practice in metabolomic MR studies to ensure an adequate number of IVs for all analytical steps. To ensure independence among the selected IVs, a clumping procedure was performed with a linkage disequilibrium (LD) threshold of r^2^ < 0.001 and a window size of 10,000 kb [36], using the 1000 Genomes Project European reference panel. Similarly, for the reverse MR analysis, the same IV selection strategy was applied. We selected single-nucleotide polymorphisms (SNPs) significantly associated with MM as the exposure, using the same significance threshold (*p* < 1 × 10^−5^) and clumping parameters. For both analyses, the summary statistics for these SNPs were extracted directly from the corresponding outcome GWAS summary statistics files (in VCF format). Specifically, the genetic associations with metabolite levels (as outcomes for reverse MR) and with MM risk (as outcomes for forward MR) were extracted from their respective VCF files. If a specific SNP was not present in the outcome data, it was automatically excluded. Data harmonization was conducted to ensure that the effect alleles for the exposure and outcome were consistent across datasets. During the harmonization process, palindromic SNPs with intermediate allele frequencies (i.e., ambiguous strand orientation) were explicitly excluded to eliminate strand alignment bias between exposure and outcome GWAS datasets. Additionally, the strength of the selected IVs was evaluated using the F-statistic, which was calculated as the square of the ratio of the effect size (Beta) to the standard error (SE) for each SNP-exposure association, a widely accepted approximation in MR analyses. An F-statistic > 10 was considered indicative of sufficient IV strength, indicating a low risk of weak instrument bias.

### 4.4. Mendelian Randomization and Sensitivity Analysis

The IVW method was employed as the primary approach for both forward (metabolites to MM) and reverse (MM to metabolites) MR analyses to estimate causal effects, with a *p*-value < 0.05 in IVW considered statistically suggestive of a causal association. This nominal significance threshold (*p* < 0.05) was adopted because the present study was designed as a discovery-phase analysis focused on identifying potential metabolite-MM causal associations. Applying a strict multiple testing correction (e.g., Bonferroni correction) at the MR stage would likely result in unacceptably high Type II error rates (false negatives) given the available sample size, which could lead to the omission of biologically relevant causal signals. Instead of relying solely on *p*-values to validate causal associations, we prioritized the consistency of results across multiple complementary MR methods (IVW, MR Egger, Weighted Median) and multi-omics integration support, which enhances the reliability of our discovery-phase findings. MR Egger and WM were used as supplementary methods to verify their robustness [37,38]. To evaluate the robustness and validity of the MR findings, sensitivity analyses were performed. Cochran’s Q statistic was calculated to assess heterogeneity among IVs and the MR-Egger regression intercept was examined to detect horizontal pleiotropy for the forward MR analysis [39,40,41]. Robustness against outlier-induced instability was systematically assessed through stepwise exclusion of individual SNPs within a LOO framework [40,42]. The Steiger directionality test was applied to empirically confirm the causal direction for the reverse MR analysis [43]. All MR analyses were conducted using the TwoSampleMR package.

### 4.5. Metabolic Pathway Analyses

To understand the biological functions of the identified causal metabolites, we performed metabolic pathway analysis using the MetaboAnalyst 6.0 platform [44]. Only metabolites with valid HMDB or KEGG IDs were included in the enrichment analysis; metabolites without known pathway mappings (i.e., those not annotated in any KEGG pathway) were excluded from this specific analytical step. Pathway enrichment was assessed using Fisher’s Exact Test, and pathway topology analysis was based on relative-betweenness centrality. The reference pathway library was homo sapiens, and the algorithm considered all compounds in the selected pathway library as the background. Consistent with the discovery-phase nature of this study and as noted in previous sections, no pathway-level multiple testing correction was applied to avoid excessively high Type II error rates that could obscure biologically relevant suggestive pathway associations.

### 4.6. Mapping SNPs to Genes and Identification of Differentially Expressed Genes

The significant IVs (SNPs) associated with the identified causal metabolites were mapped to potential target genes using the web-based tool SNPnexus (version 4) “https://www.snp-nexus.org/v4/ (accessed on 5 January 2026)” [45]. SNPnexus mapping used a genomic window of 0 kb (direct overlap) and extended upstream/downstream regions of 2 kb to capture regulatory variants; we considered genes that directly overlapped with the SNP loci as well as upstream and downstream genes within a defined genomic window. The mapped gene symbols were standardized, and ensemble IDs were converted to Gene Symbols using the org.Hs.eg.db package. The resulting gene set is referred to as the MAGs. Using the GSE153380 dataset, samples were categorized into the MM group (multiple myeloma samples) and the Normal group (healthy donor samples), excluding cell lines. Differential expression analysis was performed using the DESeq2 package with a design formula specified as ~Group to model expression differences between MM and Normal samples. Prior to statistical testing, low-expression genes (count < 10 in fewer than 3 samples) were filtered out to reduce noise. Normalization was performed via DESeq2′s default size factor method to account for variations in library size across samples, and gene-wise dispersion values were estimated using an empirical Bayes shrinkage approach (the default in the DESeq() function) to model biological and technical variability in the dataset. DEGs were identified based on an adjusted *p*-value (padj) < 0.05 and an absolute log2 fold change (|log2FC|) > 1.

### 4.7. Identification and Functional Enrichment Analysis of Overlapping Genes

To identify potential key mediator genes, we performed an intersection between the MAGs and the DEGs (including both up- and down-regulated genes). The genes common to both sets are defined as the “Overlapping Genes”. Functional enrichment analyses, including Gene Ontology (GO) (covering Biological Process, Cellular Component, and Molecular Function) and Kyoto Encyclopedia of Genes and Genomes (KEGG) pathway analyses, were performed on the overlapping genes using the clusterProfiler package [46]. For the GO enrichment analysis, terms with an adjusted *p*-value (p.adjust) < 0.05 and a Q-value < 0.2 (Benjamini-Hochberg correction) were considered statistically significant. For the KEGG pathway enrichment analysis, pathways with a *p*-value < 0.05 were considered significant.

### 4.8. Statistical Analysis Environment

Data processing, statistical analyses, and all graphical visualizations were conducted in the R software (v4.5.1). The key packages and their versions used in this study included TwoSampleMR (v0.6.25), org.Hs.eg.db (v3.21.0), DESeq2 (v1.48.2), and clusterProfiler (v4.16.0). Random seed setting was not required for the deterministic analyses performed (non-stochastic MR, pathway enrichment, and DEG analysis). All custom R scripts are available from the corresponding author upon request to ensure reproducibility.

## 5. Conclusions

These findings suggest associations between 21 serum metabolites (8 protective and 13 risk-promoting) and MM through MR, highlighting that valine/leucine/isoleucine biosynthesis and biotin metabolism may be potential key pathways in MM progression, with potential links to amino acid metabolic reprogramming and epigenetic activation. Multi-omics integration revealed co-enriched pathways of metal ion transmembrane transport and glycosaminoglycan biosynthesis, suggesting that they may play potential roles in modulating epigenetic enzyme activity (e.g., DNA methyltransferases reliant on metal cofactors) and extracellular matrix remodeling. These findings highlight metabolic-epigenetic crosstalk as a potential critical axis in MM, providing putative diagnostic markers and therapeutic targets for further exploration.

## Figures and Tables

**Table 1:** MR analysis of causal effects of significant serum metabolites on MM risk with heterogeneity and horizontal pleiotropy assessment.

Metabolite	nSNP	Cochran’s Q Test (*p*-Value)		MR-Egger Intercept	
		IVW	MR Egger	Egger Intercept	*p*-Value
Isoleucine	18	0.888	0.915	−0.0001	0.259
Lysine	18	0.202	0.454	0.0004	0.031
Methionine	20	0.241	0.204	−0.0001	0.651
3-methyl-2-oxovalerate	32	0.486	0.445	0.0000	0.632
Dihomo-linoleate (20:2n6)	9	0.995	0.987	0.0000	0.843
1,6-anhydroglucose	14	0.714	0.660	0.0000	0.620
Dimethylarginine (SDMA + ADMA)	32	0.596	0.585	0.0001	0.388
Trans-4-hydroxyproline	5	0.366	0.256	−0.0001	0.692
Scyllo-inositol	12	0.653	0.595	−0.0001	0.572
Glutaroyl carnitine	29	0.425	0.384	0.0000	0.635
10-heptadecenoate (17:1n7)	5	0.808	0.815	−0.0002	0.476
1-docosahexaenoylglycerophosphocholine *	6	0.592	0.625	−0.0002	0.353
N-acetylthreonine	12	0.485	0.437	−0.0001	0.507
1-oleoylglycerophosphocholine	16	0.324	0.261	0.0000	0.905
X-08988	31	0.912	0.898	0.0000	0.558
X-01911	16	0.707	0.656	0.0000	0.626
X-12038	51	0.552	0.561	−0.0001	0.275
X-12734	10	0.337	0.269	0.0000	0.679
X-12847	8	0.519	0.417	0.0000	0.740
X-13069	16	0.906	0.870	0.0000	0.800
X-14056	8	0.899	0.847	0.0000	0.674

nSNP: The number of single-nucleotide polymorphisms (SNPs). Cochran’s Q test: Tests if the variation in results across studies is greater than expected by chance. A *p*-value > 0.05 indicates no significant heterogeneity between studies. IVW: Inverse variance-weighted (giving more weight to studies with more precise estimates). An IVW estimate of 1.5 suggests a positive effect, with a 1.5 times increase in the outcome for each unit increase in the exposure. MR-Egger Intercept: Measures horizontal pleiotropy (bias from confounding). An intercept close to 0 indicates no significant bias. Egger intercept: Similar to the MR-Egger intercept. A non-zero intercept suggests possible pleiotropic effects. *p*: Indicates statistical significance. A *p*-value < 0.05 suggests a significant result. * Indicates metabolites for which reference spectra of the pure substances were not directly measured on the Metabolon platform.

## Data Availability

The data supporting the findings of this study are publicly available from several sources. Publicly accessible datasets used in this study include those from the IEU OpenGWAS database “https://gwas.mrcieu.ac.uk/ (accessed on 12 December 2025)”; the MM dataset IDs were ieu-b-4957 “https://opengwas.io/datasets/ieu-b-4957 (accessed on 12 December 2025)”. GWAS data of metabolomics can be found here “https://opengwas.io/datasets/ (accessed on 12 December 2025)”. Transcriptomic data can be found here “https://www.ncbi.nlm.nih.gov/geo/query/acc.cgi?acc=GSE15338 (accessed on 20 December 2025)”.

## Supplementary Materials

The following supporting information can be downloaded at https://www.mdpi.com/article/10.3390/ijms27041904/s1.

## Abbreviations

MM: Multiple myeloma; MR: Mendelian randomization; IVW: inverse variance-weighted; WM: weighted median; LOO: leave-one-out; BCAA: branched-chain amino acid; ASCT: Autologous hematopoietic stem cell transplantation; GWAS: Genome-wide association study; SNPs: Single-nucleotide polymorphisms; IVs: Instrumental variables; LD: linkage disequilibrium; DEGs: differentially expressed genes; MAGs: Metabolite-associated genes; KEGG: Kyoto encyclopedia of genes and genomes; GO: Gene Ontology; TCA: tricarboxylic acid; GAG: glycosaminoglycan.
