# Supplementary material for: Integrating Bidirectional Mendelian Randomization with Multi-Omics Reveals Causal Serum Metabolites and Novel Metabolic Drivers of Multiple Myeloma
Source: Int J Mol Sci. 2026 Feb 16;27(4):1904. doi: 10.3390/ijms27041904 (PMC12941277; doi:10.3390/ijms27041904)
Supplement: Supplementary file 1 [file ijms-27-01904-s001.zip › Figure_S1.pdf]

Supplementary Figure S1. Funnel plots for remaining 19 metabolites with MM risk.

Funnel Plot: 1−docosahexaenoylglycerophosphocholine*

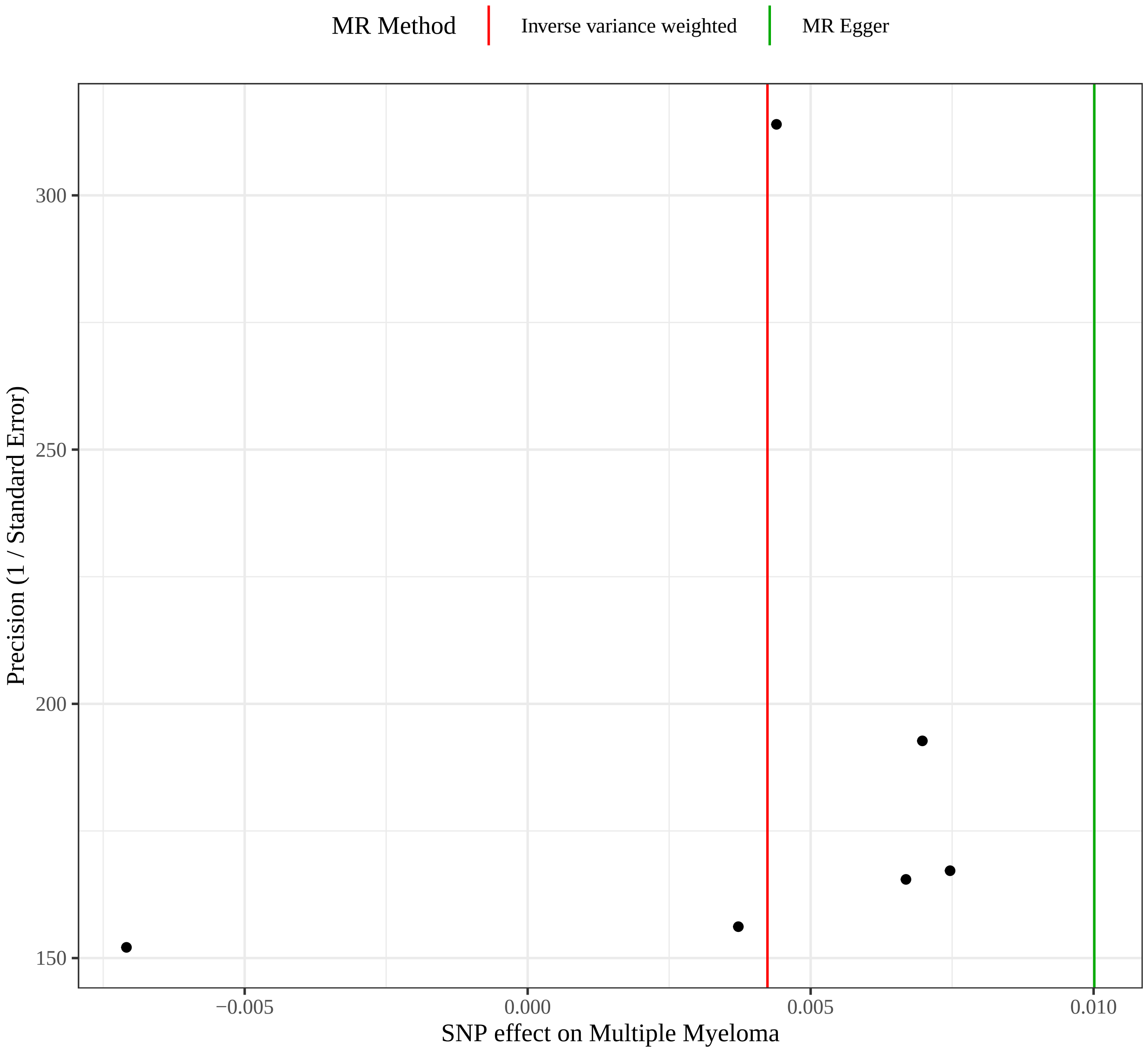

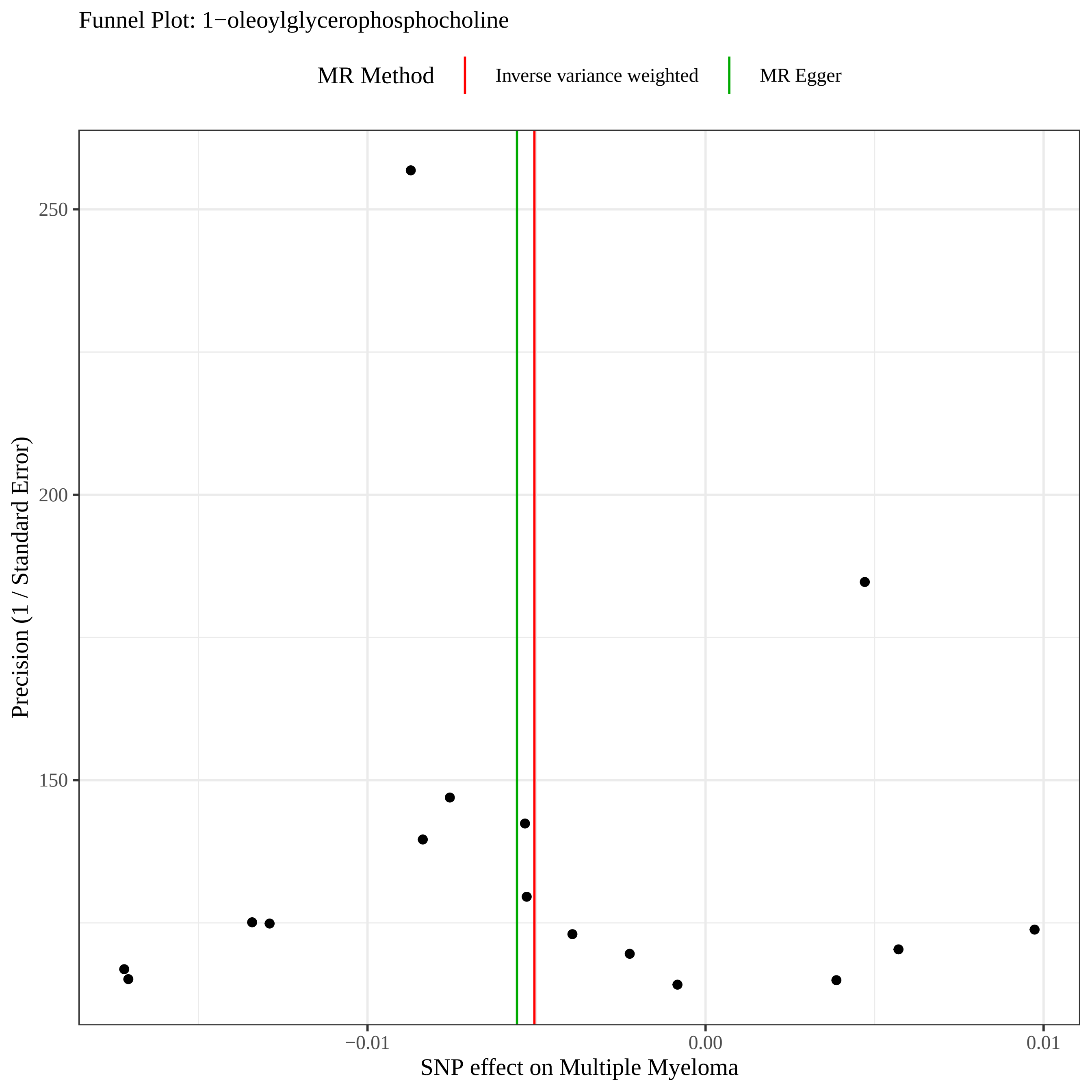

Funnel Plot: 1−oleoylglycerophosphocholine
MR Method
Inverse variance weighted
MR Egger
Precision (1 / Standard Error)
250
200
150
−0.01
0.00
0.01
SNP effect on Multiple Myeloma

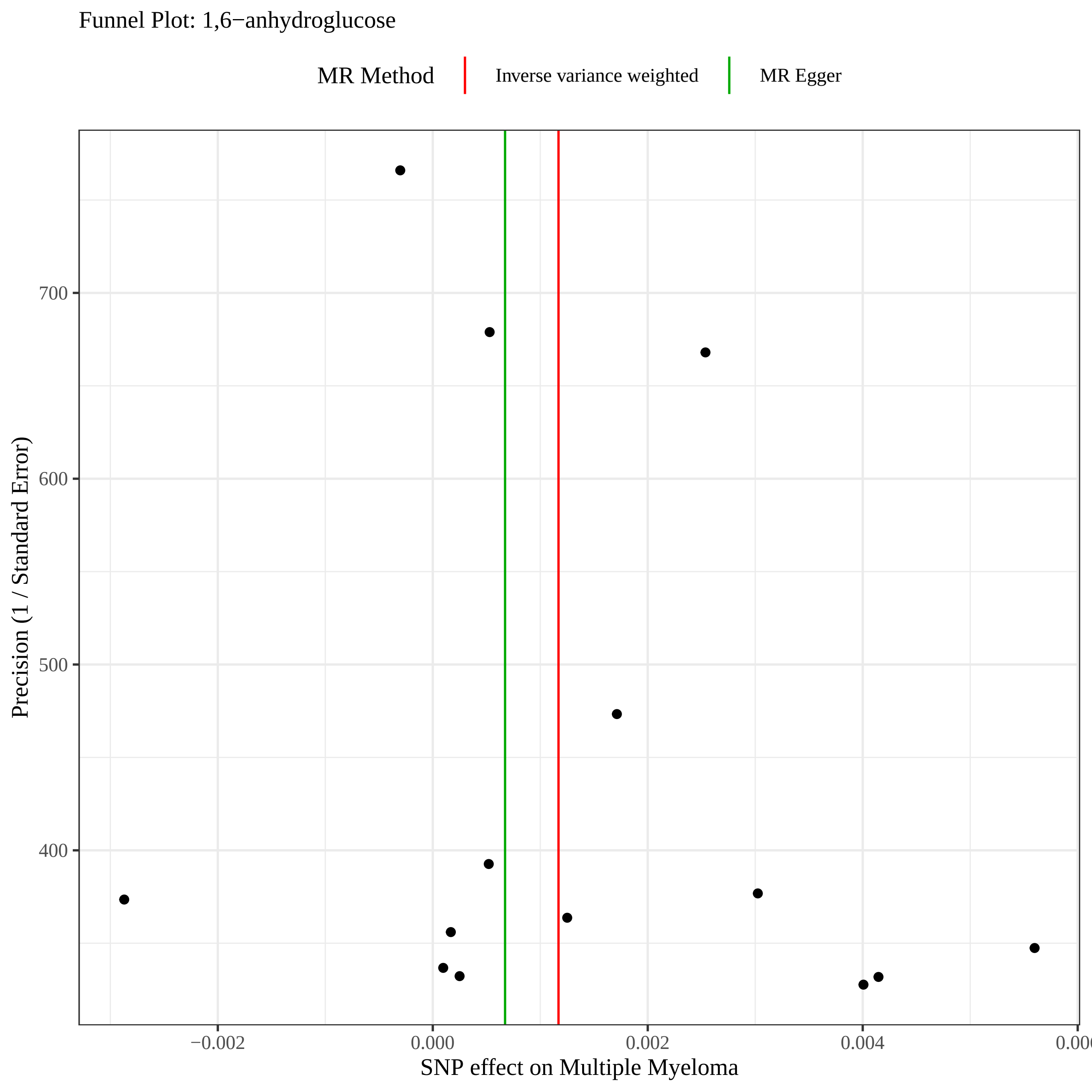
Funnel Plot: 1,6−anhydroglucose
MR Method
Inverse variance weighted
MR Egger
Precision (1 / Standard Error)
700
600
500
400
−0.002
0.000
0.002
0.004
SNP effect on Multiple Myeloma

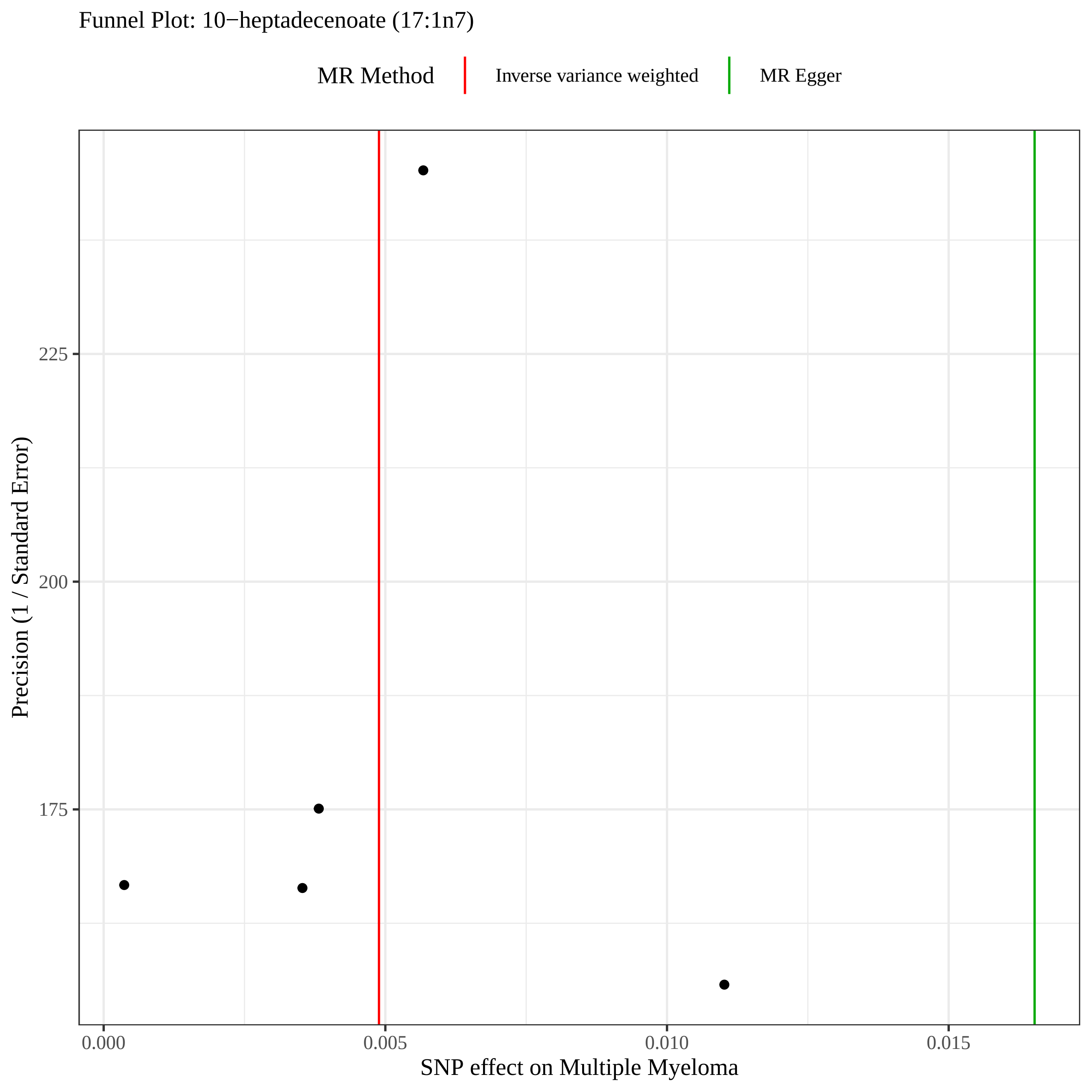

Funnel Plot: 10−heptadecenoate (17:1n7)
MR Method
Inverse variance weighted
MR Egger
Precision (1 / Standard Error)
225
200
175
0.000
0.005
0.010
0.015
SNP effect on Multiple Myeloma

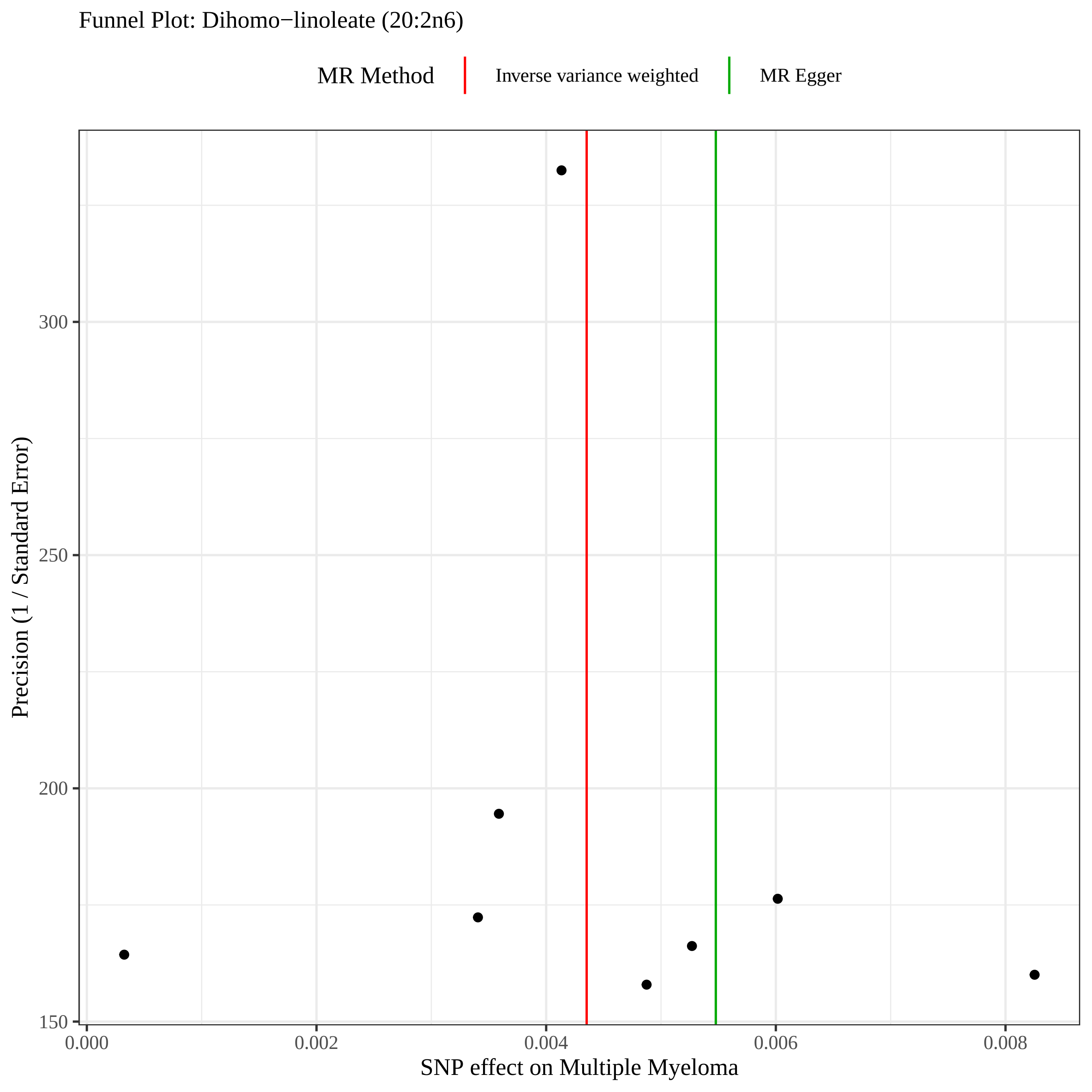
Funnel Plot: Dihomo−linoleate (20:2n6)
MR Method
Inverse variance weighted
MR Egger
Precision (1 / Standard Error)
300
250
200
150
0.000
0.002
0.004
0.006
0.008
SNP effect on Multiple Myeloma

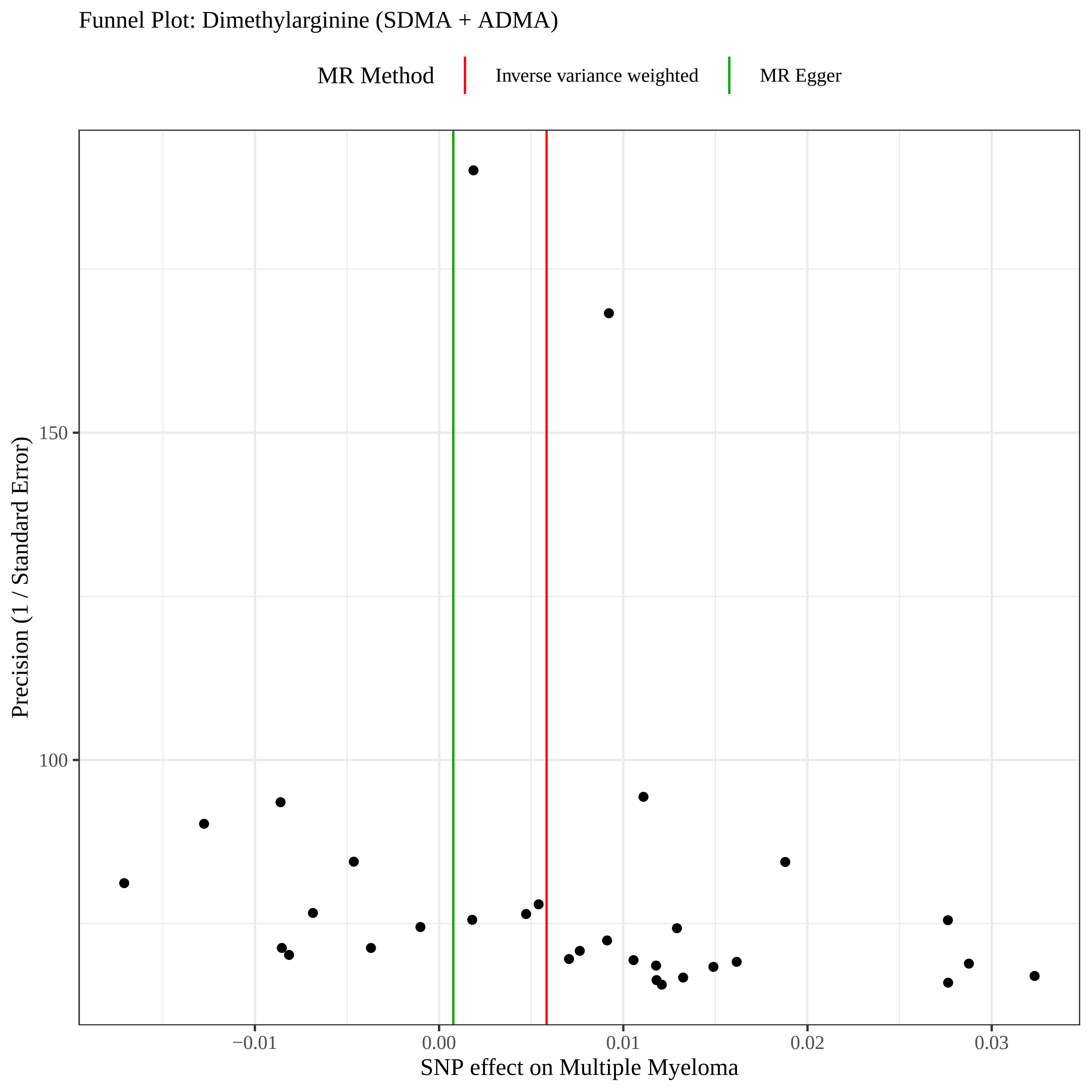

Funnel Plot: Dimethylarginine (SDMA + ADMA)
MR Method
Inverse variance weighted
MR Egger
150
100
Precision (1 / Standard Error)
−0.01
0.00
0.01
0.02
0.03
SNP effect on Multiple Myeloma

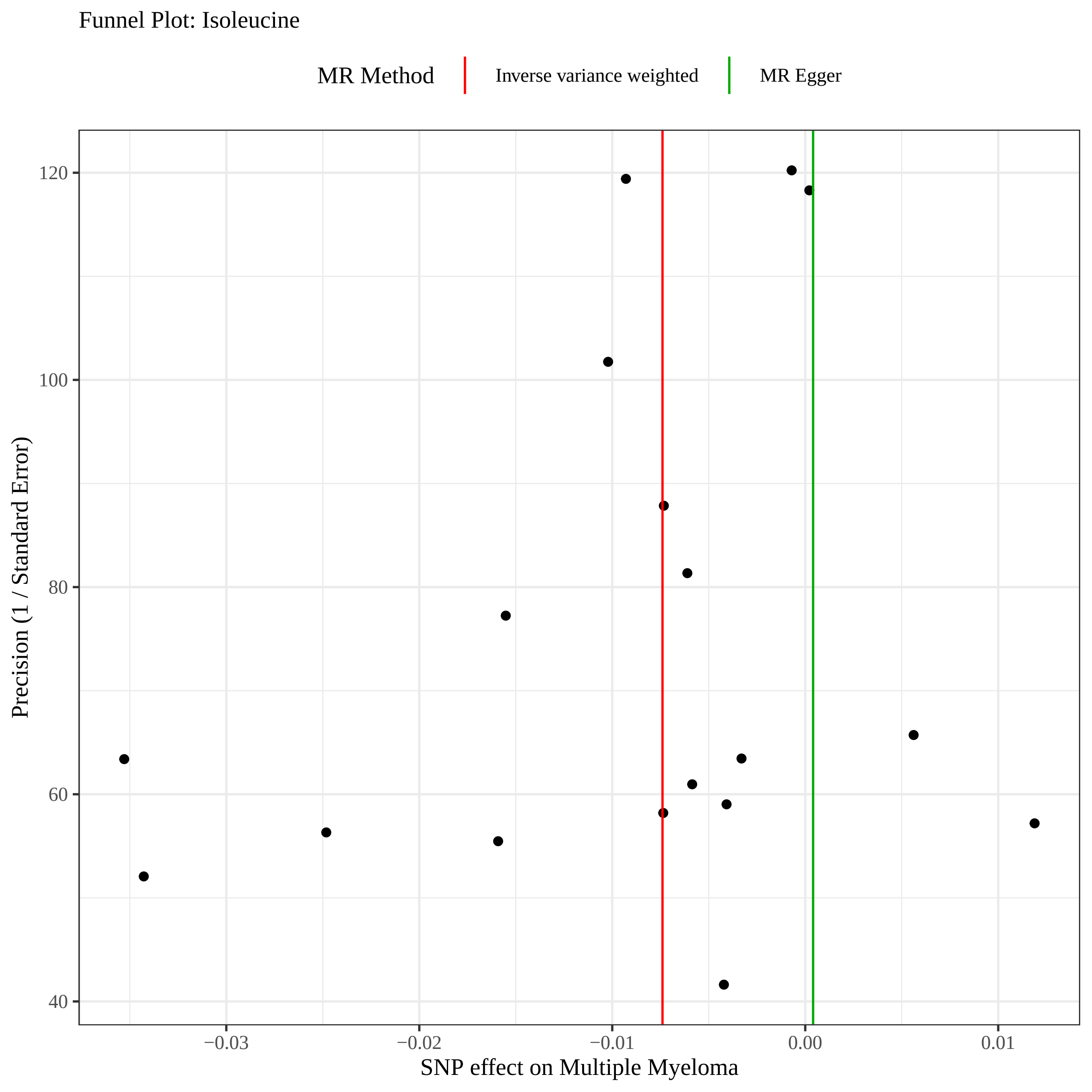

Funnel Plot: Isoleucine
MR Method
Inverse variance weighted
MR Egger
Precision (1 / Standard Error)
120
100
80
60
40
−0.03
−0.02
−0.01
0.00
0.01
SNP effect on Multiple Myeloma

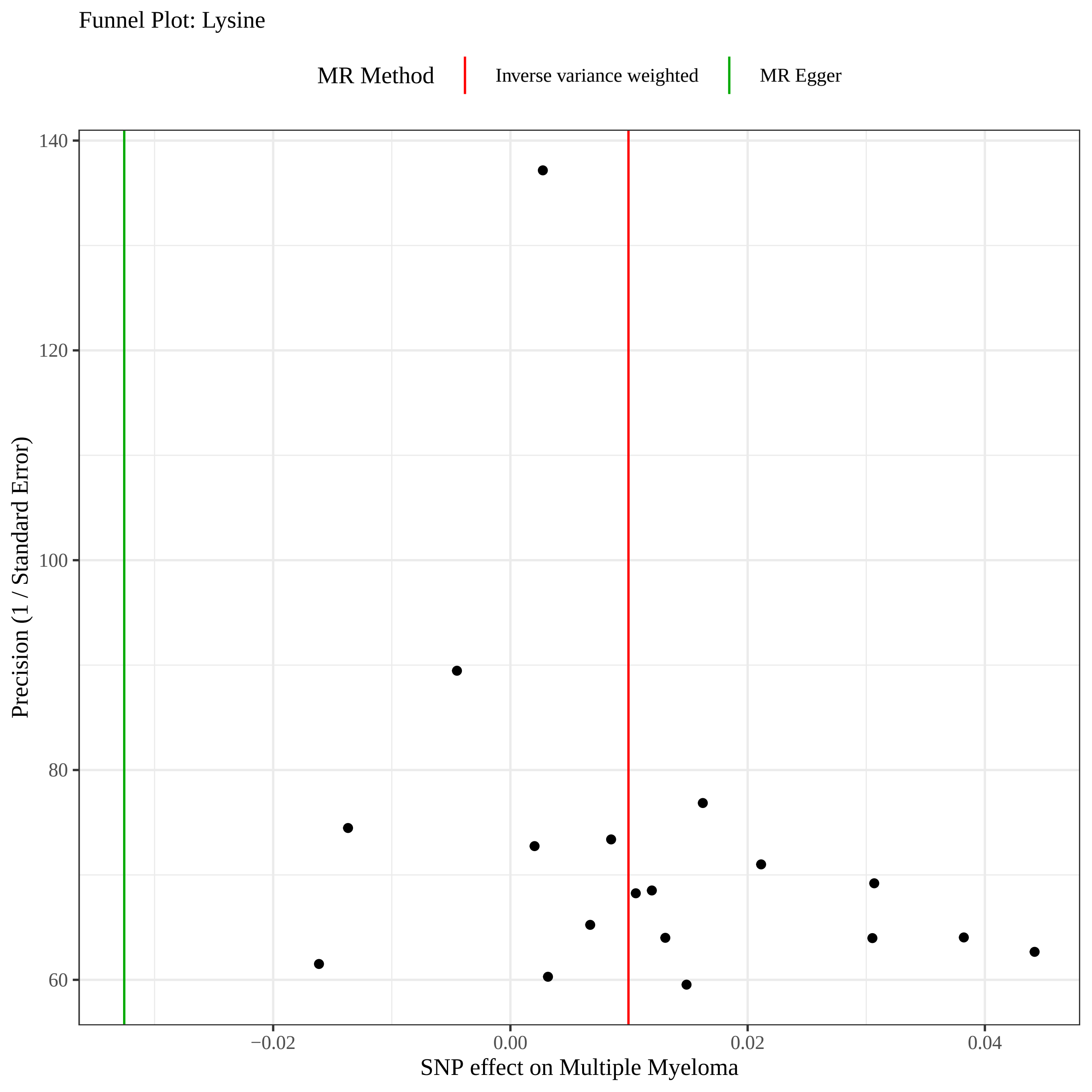
Funnel Plot: Lysine
MR Method
Inverse variance weighted
MR Egger
Precision (1 / Standard Error)
140
120
100
80
60
−0.02
0.00
0.02
0.04
SNP effect on Multiple Myeloma

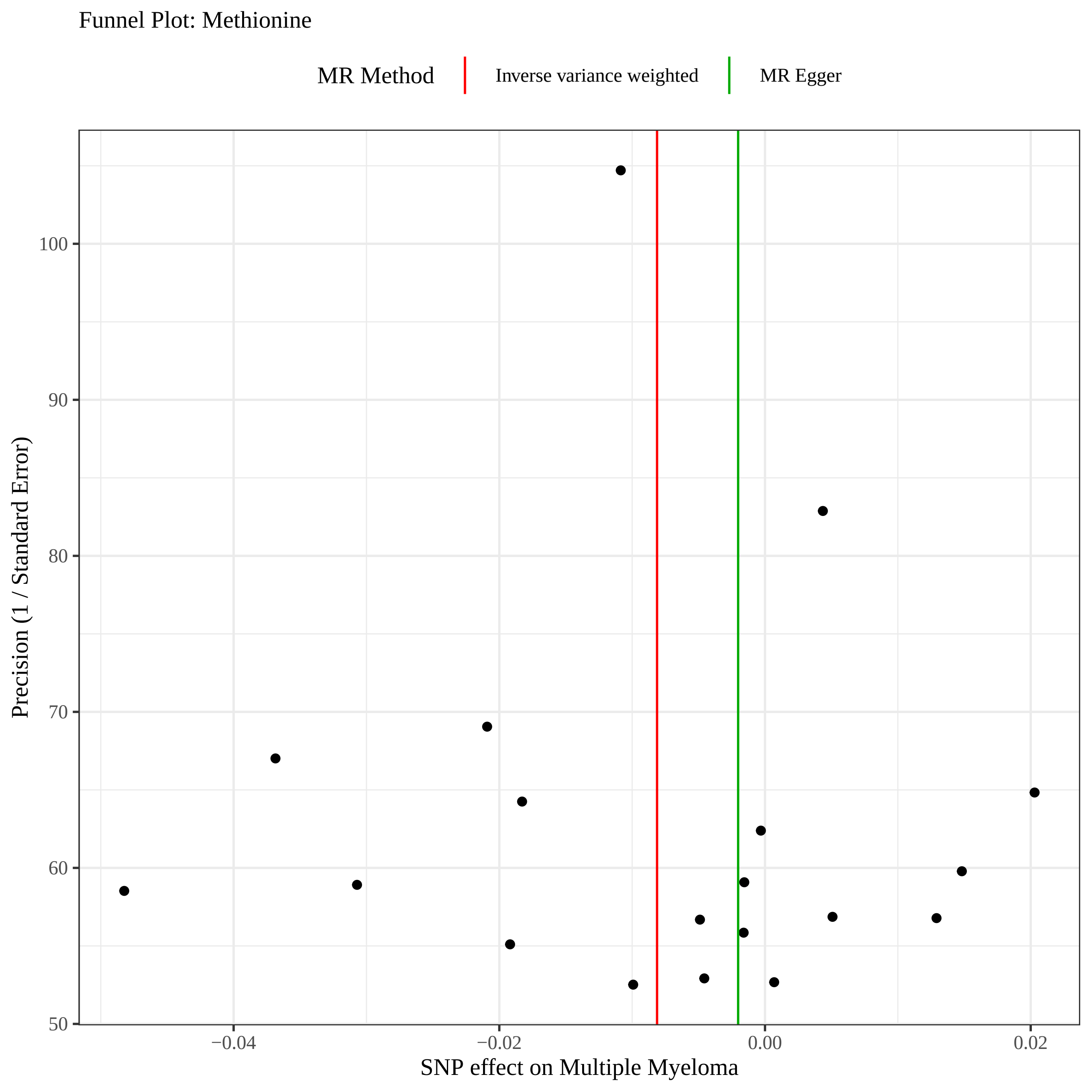

Funnel Plot: Methionine
MR Method
Inverse variance weighted
MR Egger
Precision (1 / Standard Error)
100
90
80
70
60
50
−0.04
−0.02
0.00
0.02
SNP effect on Multiple Myeloma

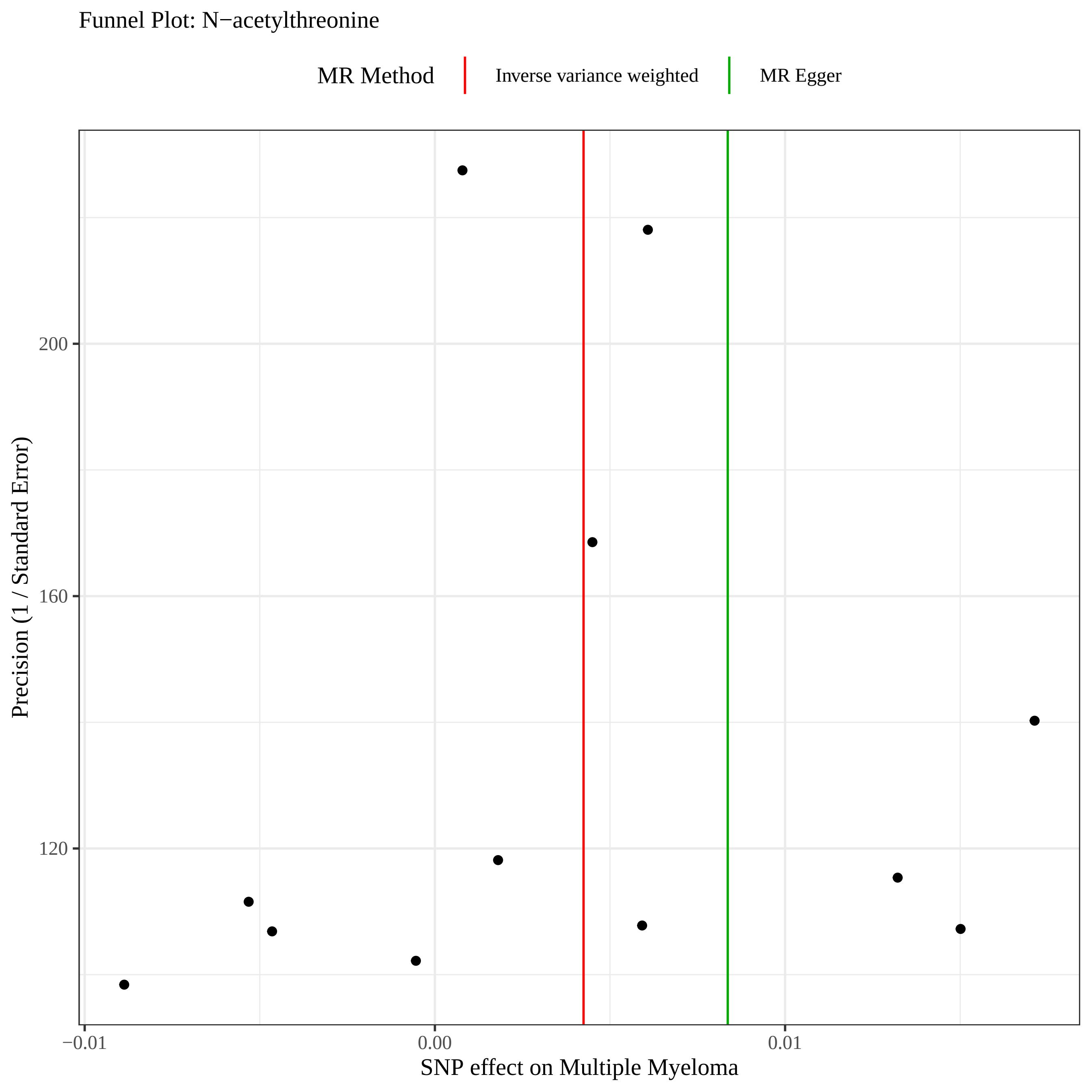
Funnel Plot: N−acetylthreonine
MR Method
Inverse variance weighted
MR Egger
Precision (1 / Standard Error)
200
160
120
−0.01
0.00
0.01
SNP effect on Multiple Myeloma

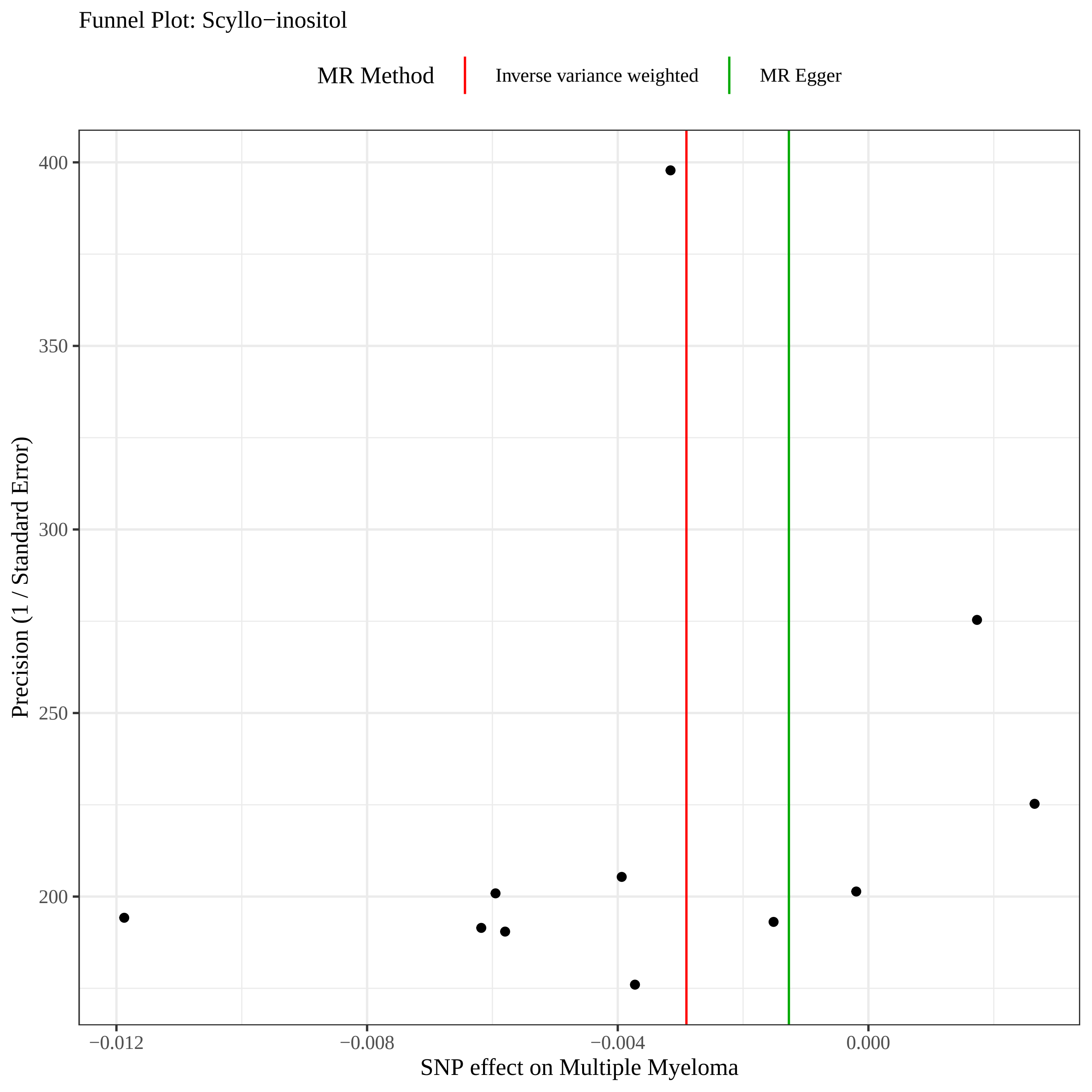
Funnel Plot: Scyllo−inositol
MR Method
Inverse variance weighted
MR Egger
Precision (1 / Standard Error)
400
350
300
250
200
−0.012
−0.008
−0.004
0.000
SNP effect on Multiple Myeloma

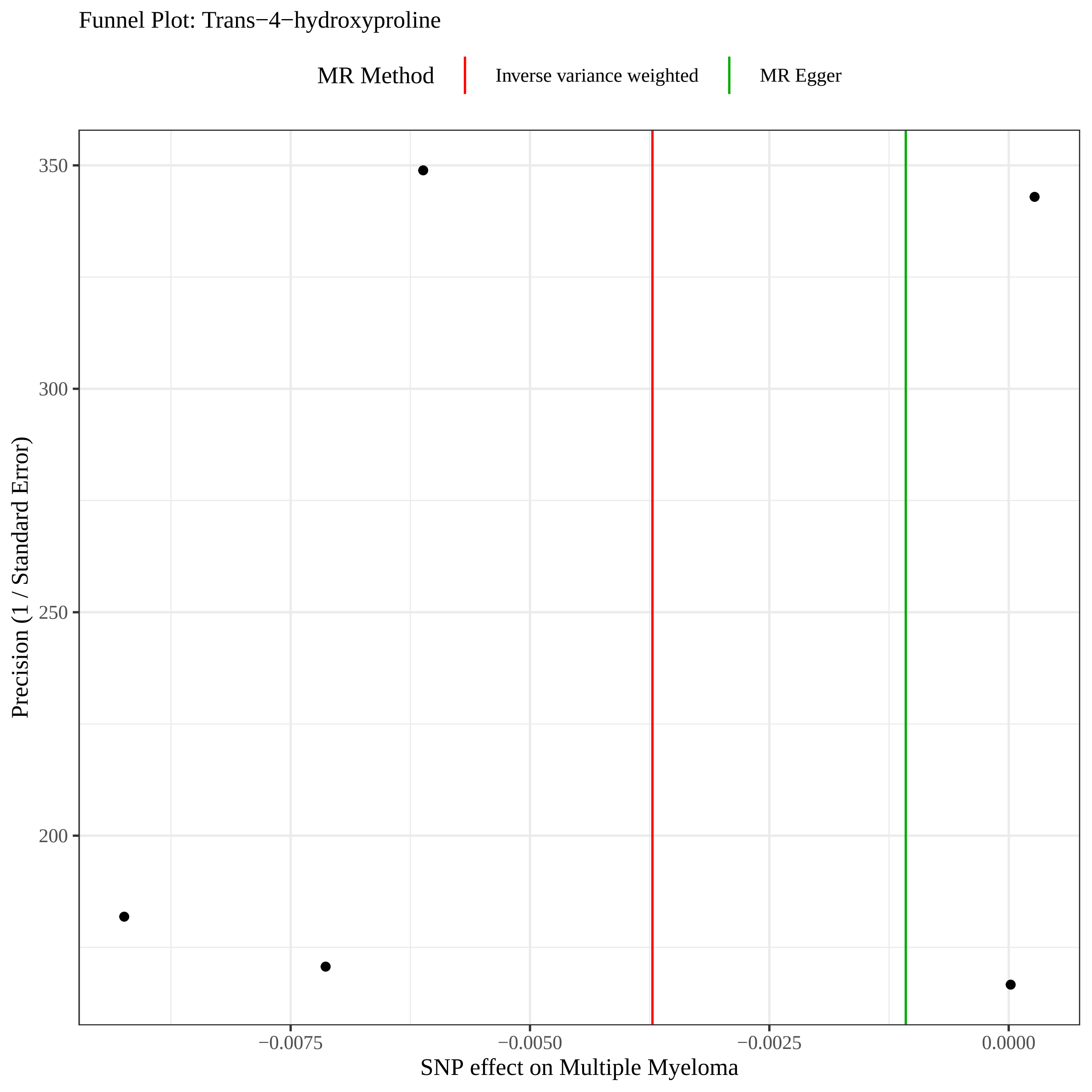

Funnel Plot: Trans−4−hydroxyproline
MR Method
Inverse variance weighted
MR Egger
Precision (1 / Standard Error)
350
300
250
200
−0.0075
−0.0050
−0.0025
0.0000
SNP effect on Multiple Myeloma

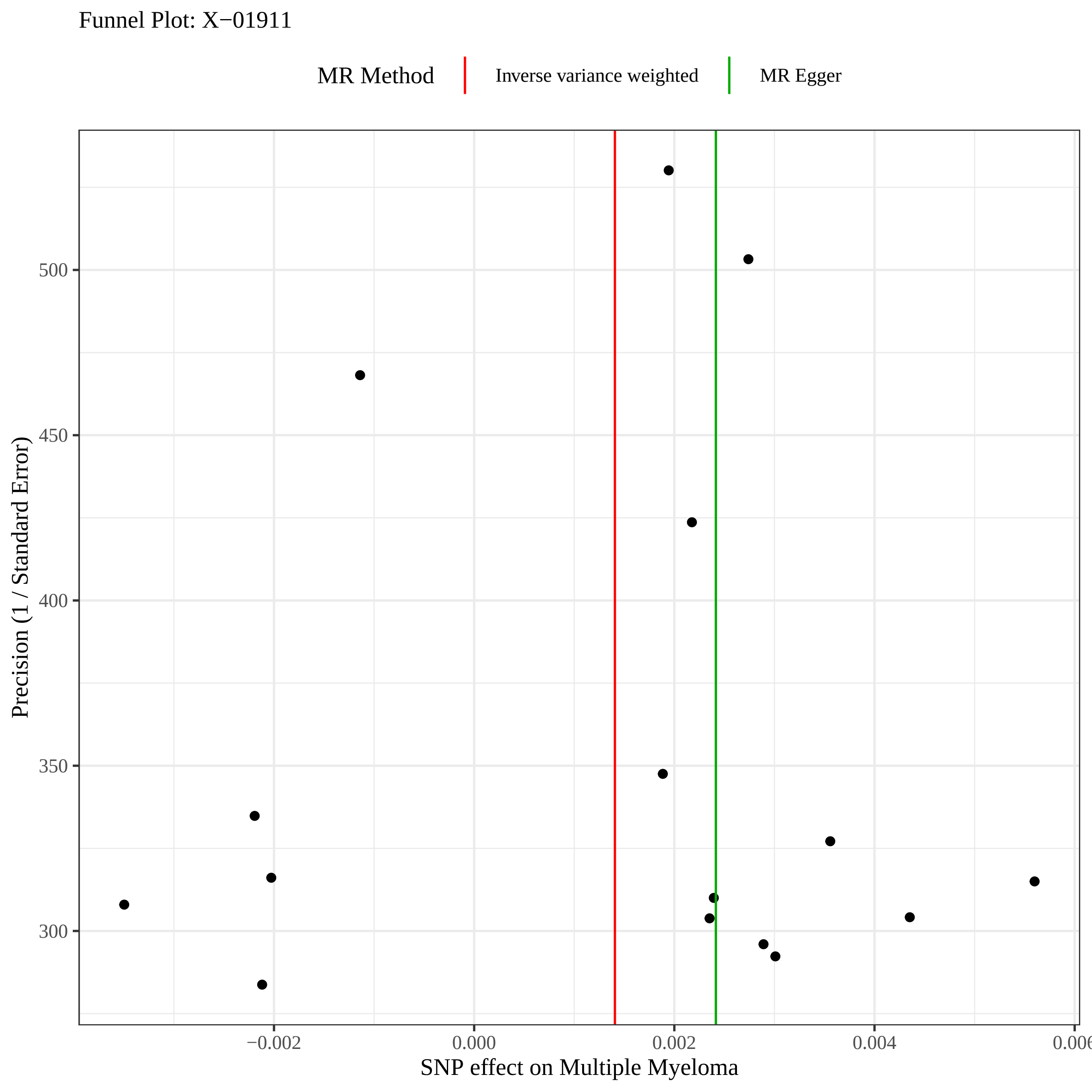

Funnel Plot: X−01911
MR Method
Inverse variance weighted
MR Egger
Precision (1 / Standard Error)
500
450
400
350
300
−0.002
0.000
0.002
0.004
0.006
SNP effect on Multiple Myeloma

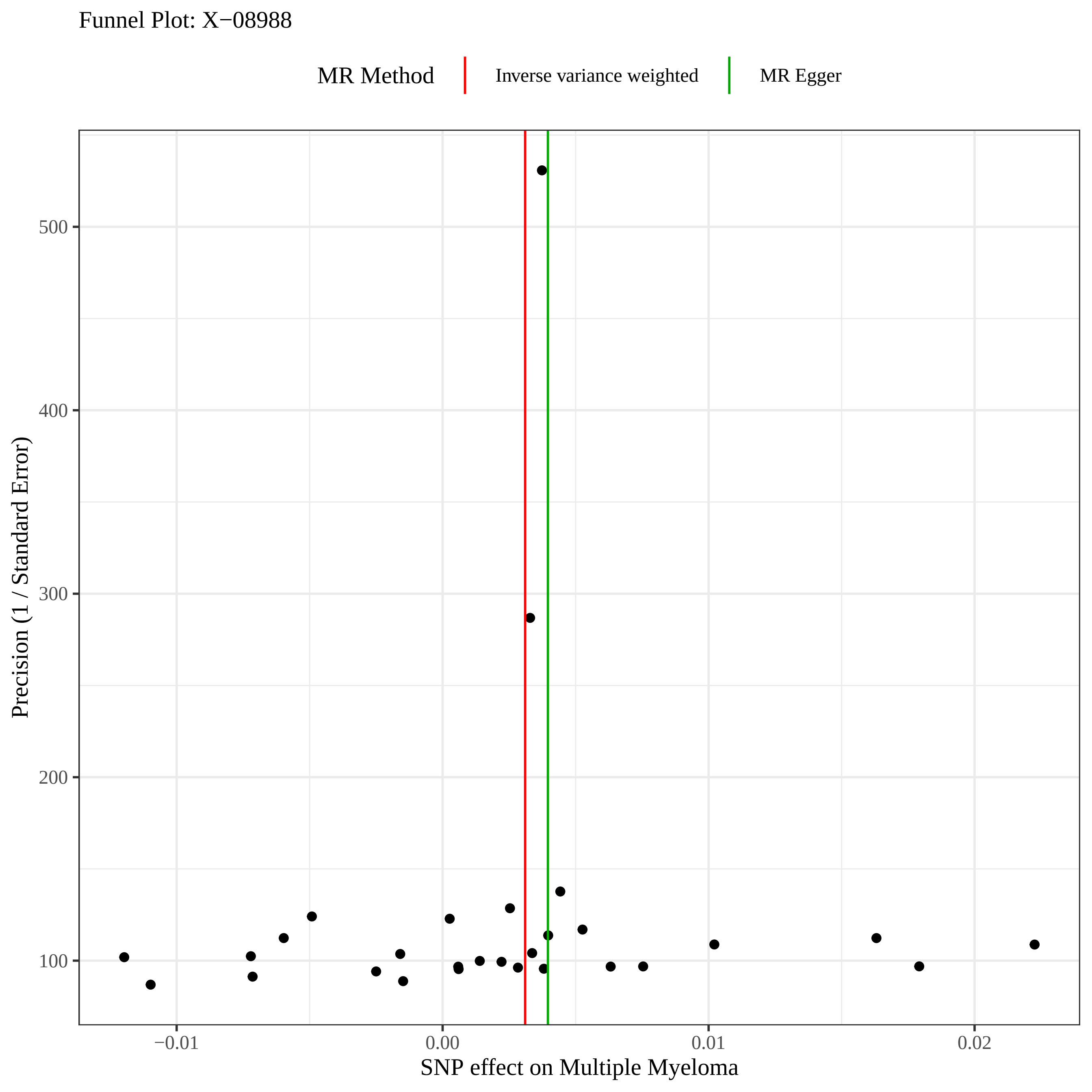

Funnel Plot: X−08988
MR Method
Inverse variance weighted
MR Egger
Precision (1 / Standard Error)
500
400
300
200
100
−0.01
0.00
0.01
0.02
SNP effect on Multiple Myeloma

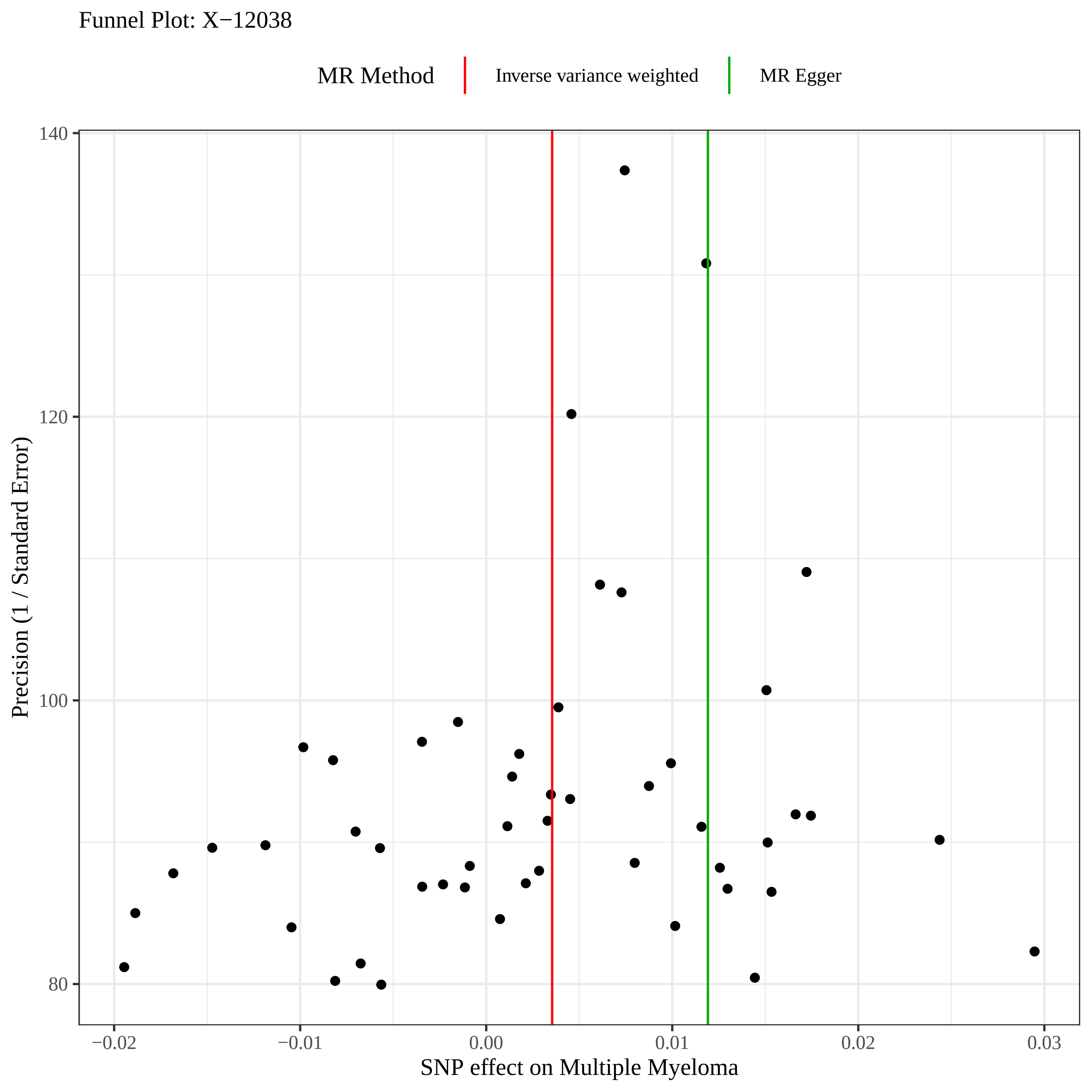
Funnel Plot: X−12038
MR Method
Inverse variance weighted
MR Egger
140
120
100
80
Precision (1 / Standard Error)
−0.02
−0.01
0.00
0.01
0.02
0.03
SNP effect on Multiple Myeloma

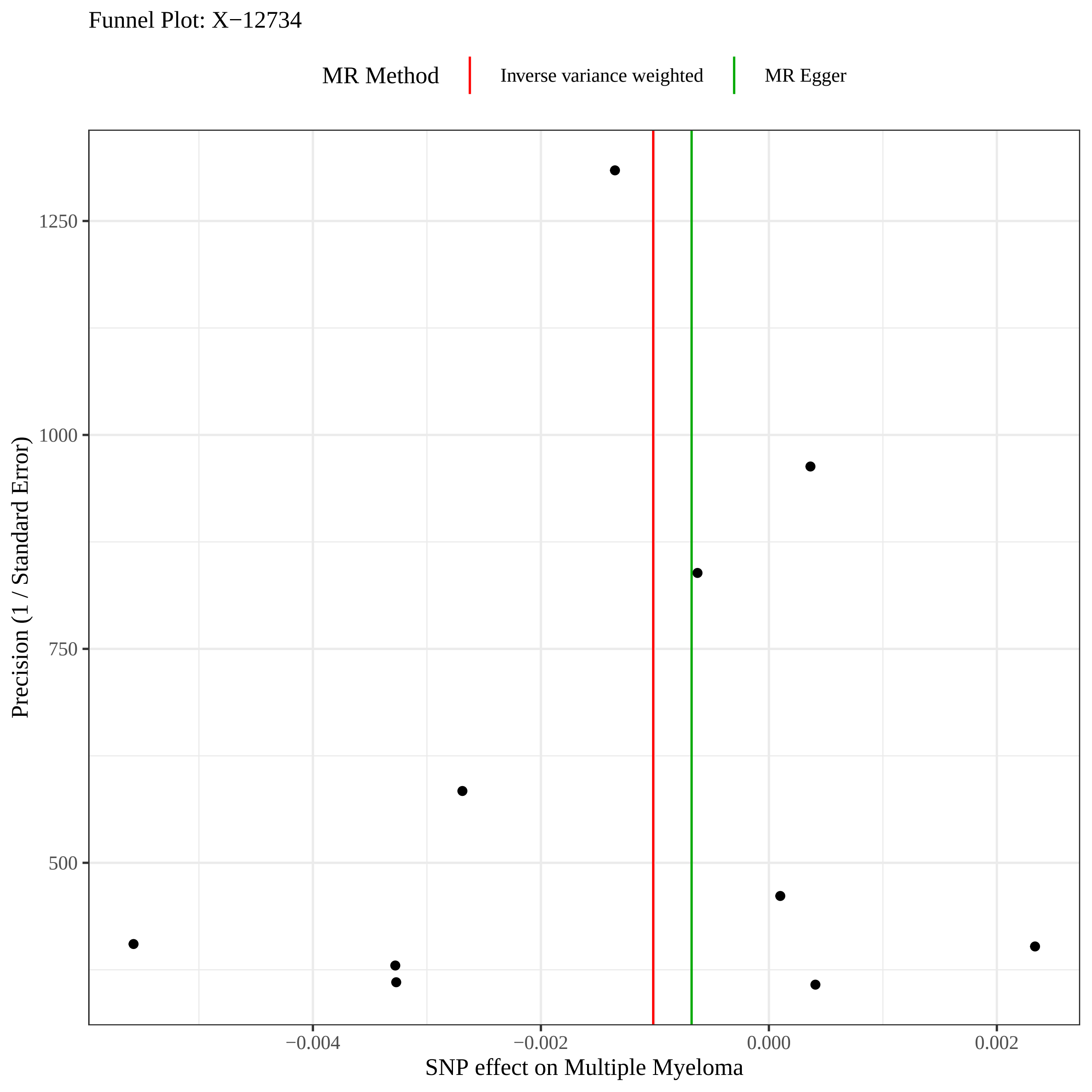
Funnel Plot: X−12734
MR Method
Inverse variance weighted
MR Egger
Precision (1 / Standard Error)
1250
1000
750
500
−0.004
−0.002
0.000
0.002
SNP effect on Multiple Myeloma

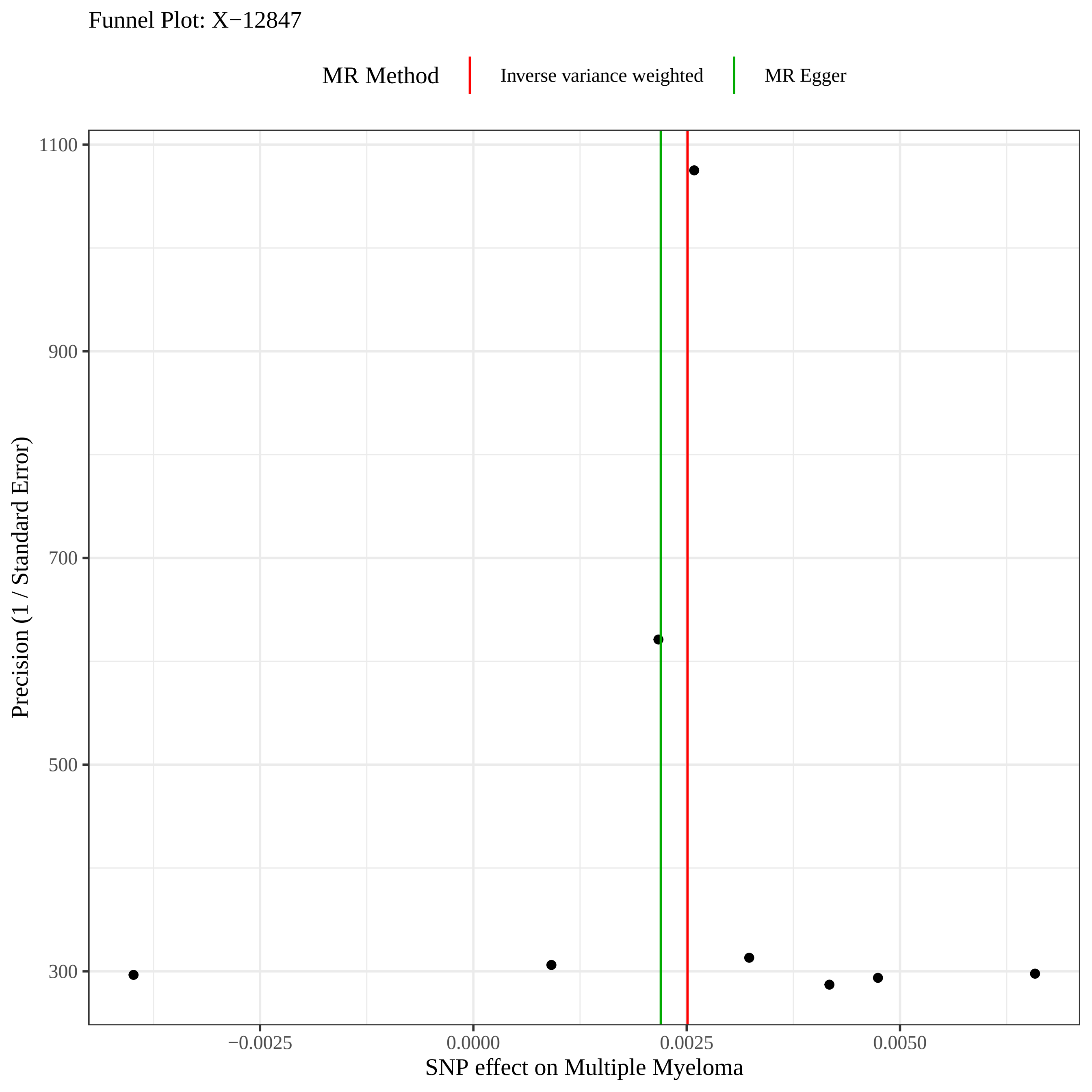
Funnel Plot: X−12847
MR Method
Inverse variance weighted
MR Egger
1100
900
700
500
300
Precision (1 / Standard Error)
−0.0025
0.0000
0.0025
0.0050
SNP effect on Multiple Myeloma

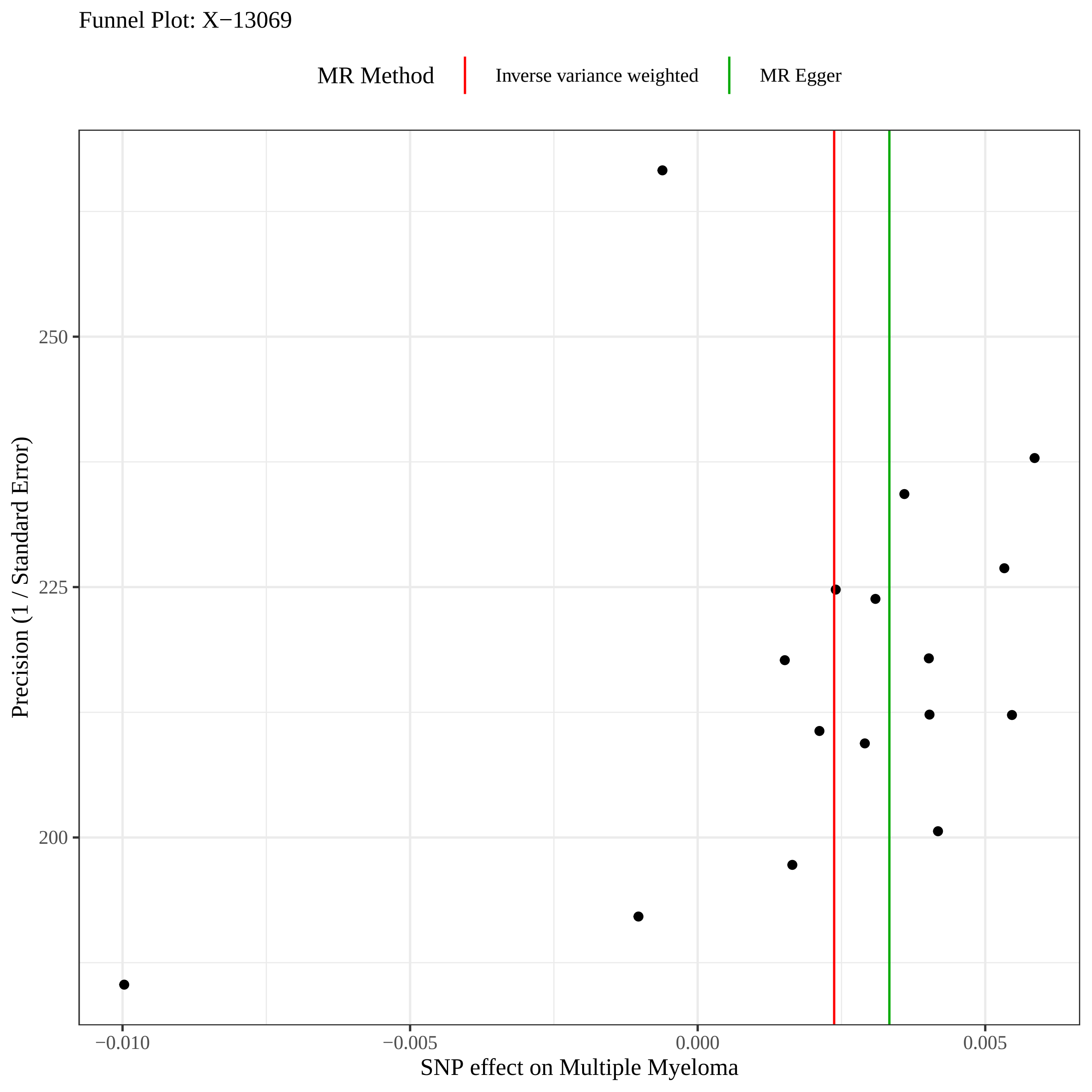

Funnel Plot: X−13069
MR Method
Inverse variance weighted
MR Egger
Precision (1 / Standard Error)
250
225
200
−0.010
−0.005
0.000
0.005
SNP effect on Multiple Myeloma

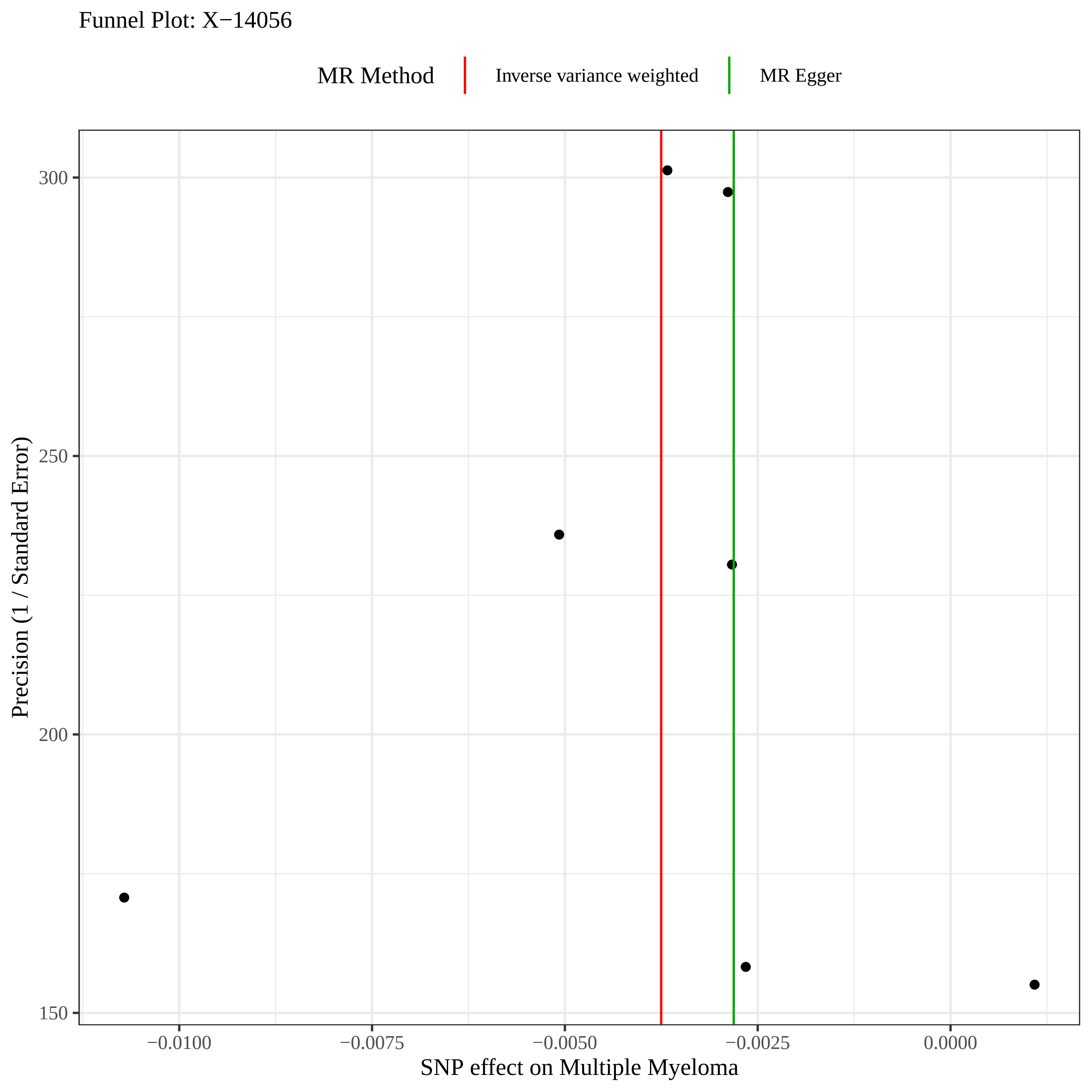

Funnel Plot: X−14056
MR Method
Inverse variance weighted
MR Egger
Precision (1 / Standard Error)
300
250
200
150
−0.0100
−0.0075
−0.0050
−0.0025
0.0000
SNP effect on Multiple Myeloma
