# Supplementary material for: Integrating Bidirectional Mendelian Randomization with Multi-Omics Reveals Causal Serum Metabolites and Novel Metabolic Drivers of Multiple Myeloma
Source: Int J Mol Sci. 2026 Feb 16;27(4):1904. doi: 10.3390/ijms27041904 (PMC12941277; doi:10.3390/ijms27041904)
Supplement: Supplementary file 1 [file ijms-27-01904-s001.zip › Figure_S2.pdf]

Supplementary Figure S2. LOO plots for remaining 19 metabolites with MM risk.

LOO: 1−docosahexaenoylglycerophosphocholine*

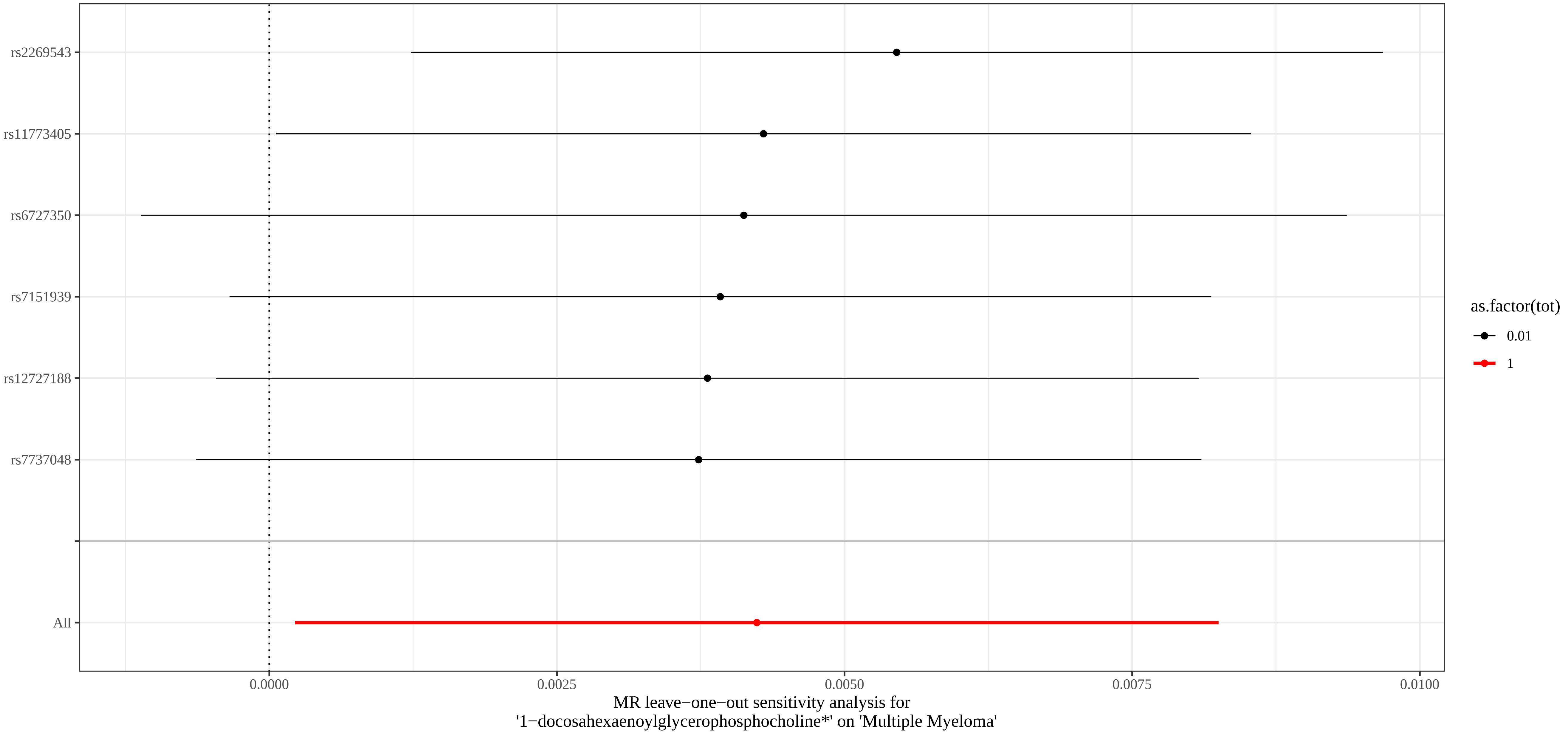

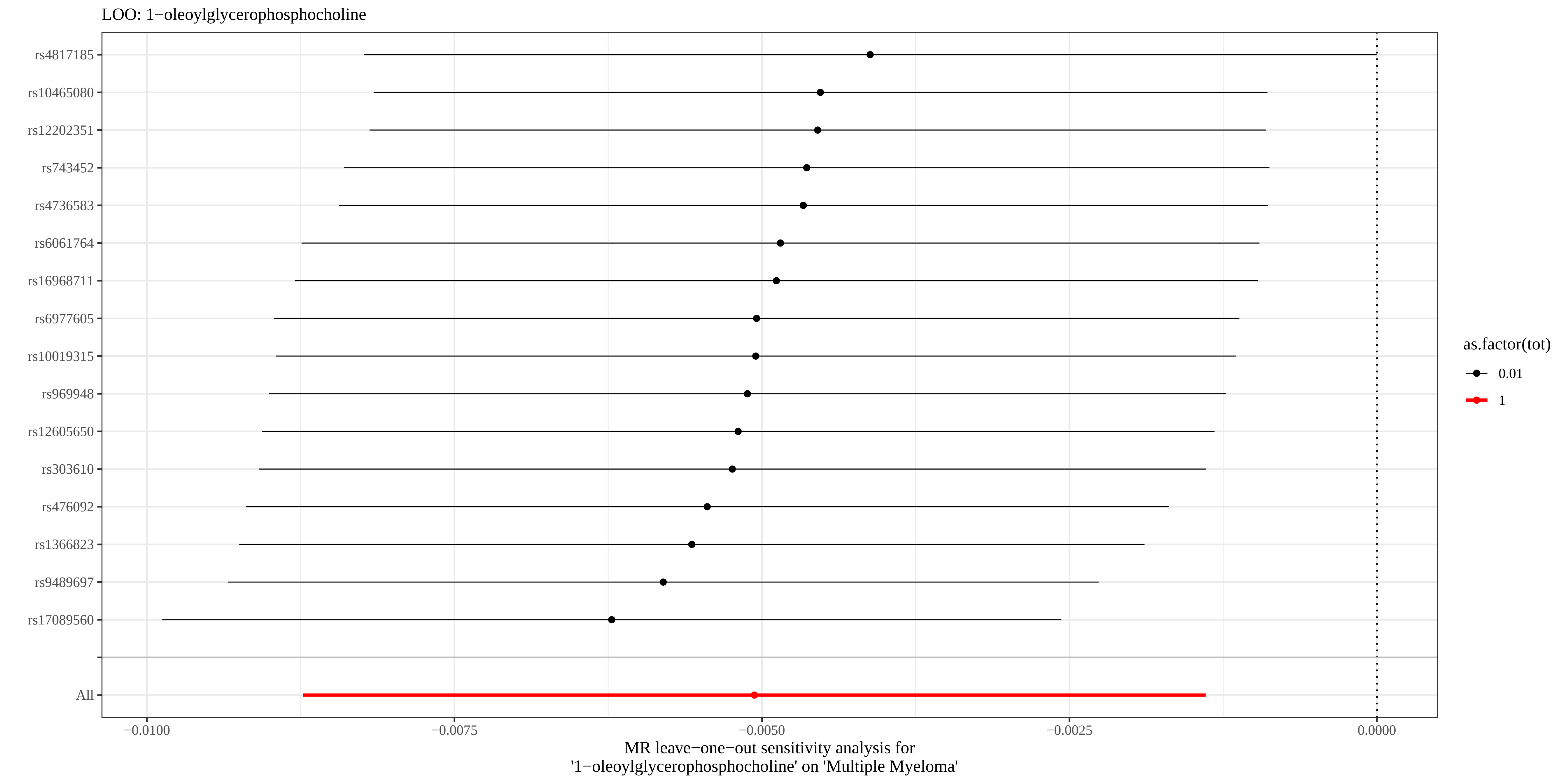

LOO: 1−oleoylglycerophosphocholine
rs4817185
rs10465080
rs12202351
rs743452
rs4736583
rs6061764
rs16968711
rs6977605
rs10019315
rs969948
rs12605650
rs303610
rs476092
rs1366823
rs9489697
rs17089560
All
−0.0100
−0.0075
−0.0050
−0.0025
0.0000
MR leave−one−out sensitivity analysis for
'1−oleoylglycerophosphocholine' on 'Multiple Myeloma'
as.factor(tot)
0.01
1

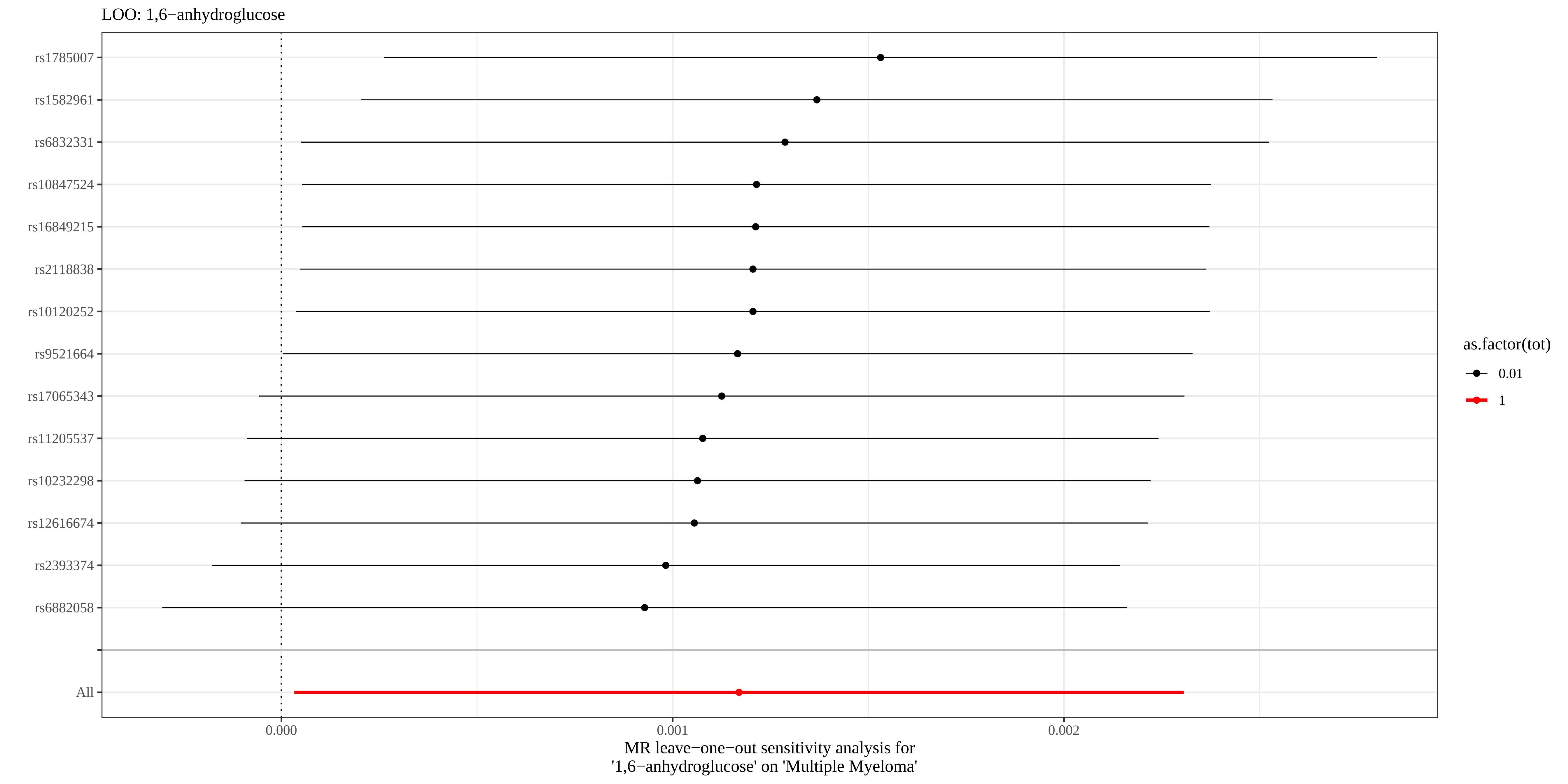

LOO: 1,6−anhydroglucose
rs1785007
rs1582961
rs6832331
rs10847524
rs16849215
rs2118838
rs10120252
rs9521664
rs17065343
rs11205537
rs10232298
rs12616674
rs2393374
rs6882058
All
0.000
0.001
0.002
MR leave−one−out sensitivity analysis for
'1,6−anhydroglucose' on 'Multiple Myeloma'
as.factor(tot)
0.01
1

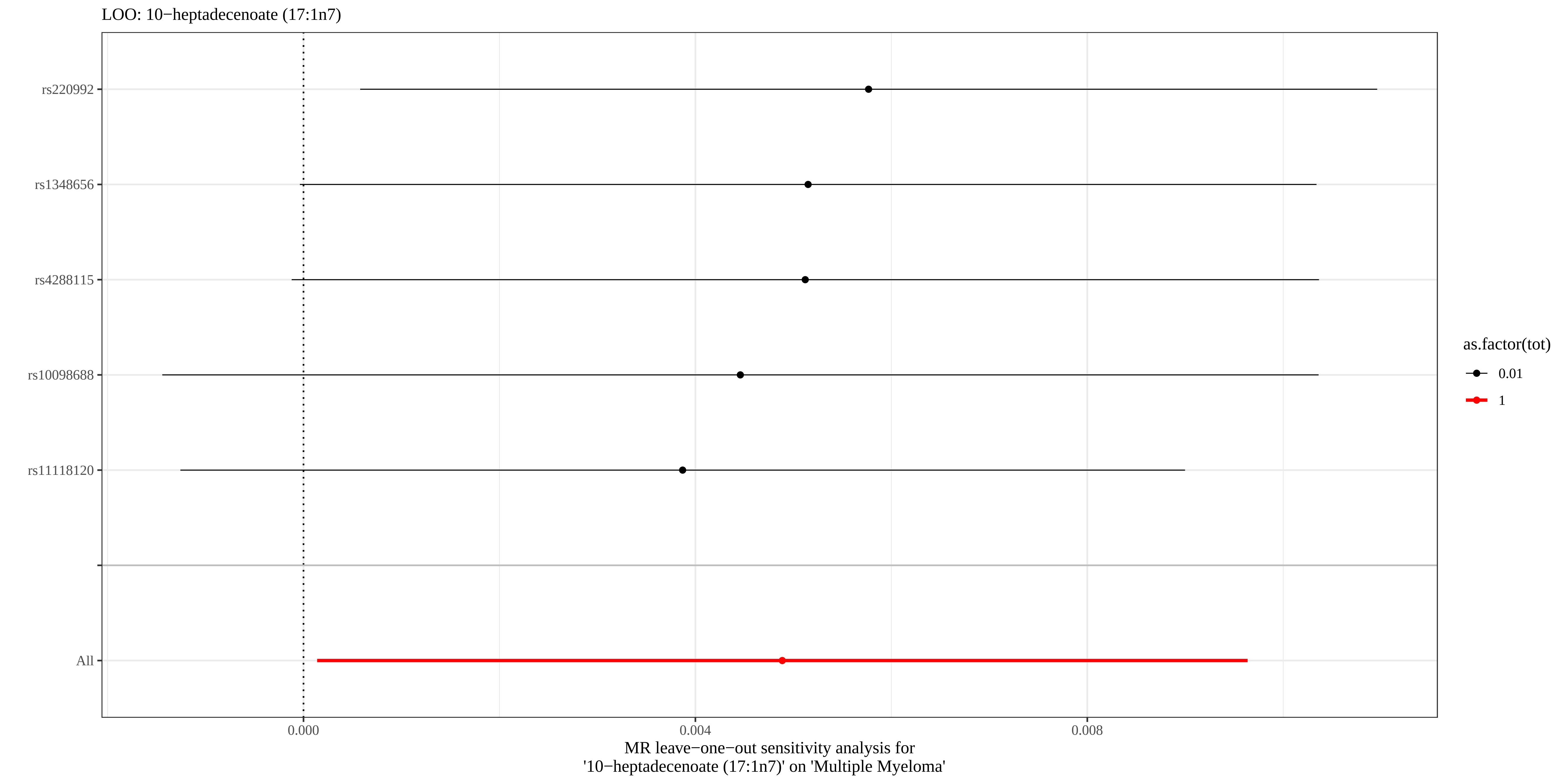
LOO: 10−heptadecenoate (17:1n7)
rs220992
rs1348656
rs4288115
rs10098688
rs11118120
All
0.000
0.004
0.008
MR leave−one−out sensitivity analysis for
'10−heptadecenoate (17:1n7)' on 'Multiple Myeloma'
as.factor(tot)
0.01
1

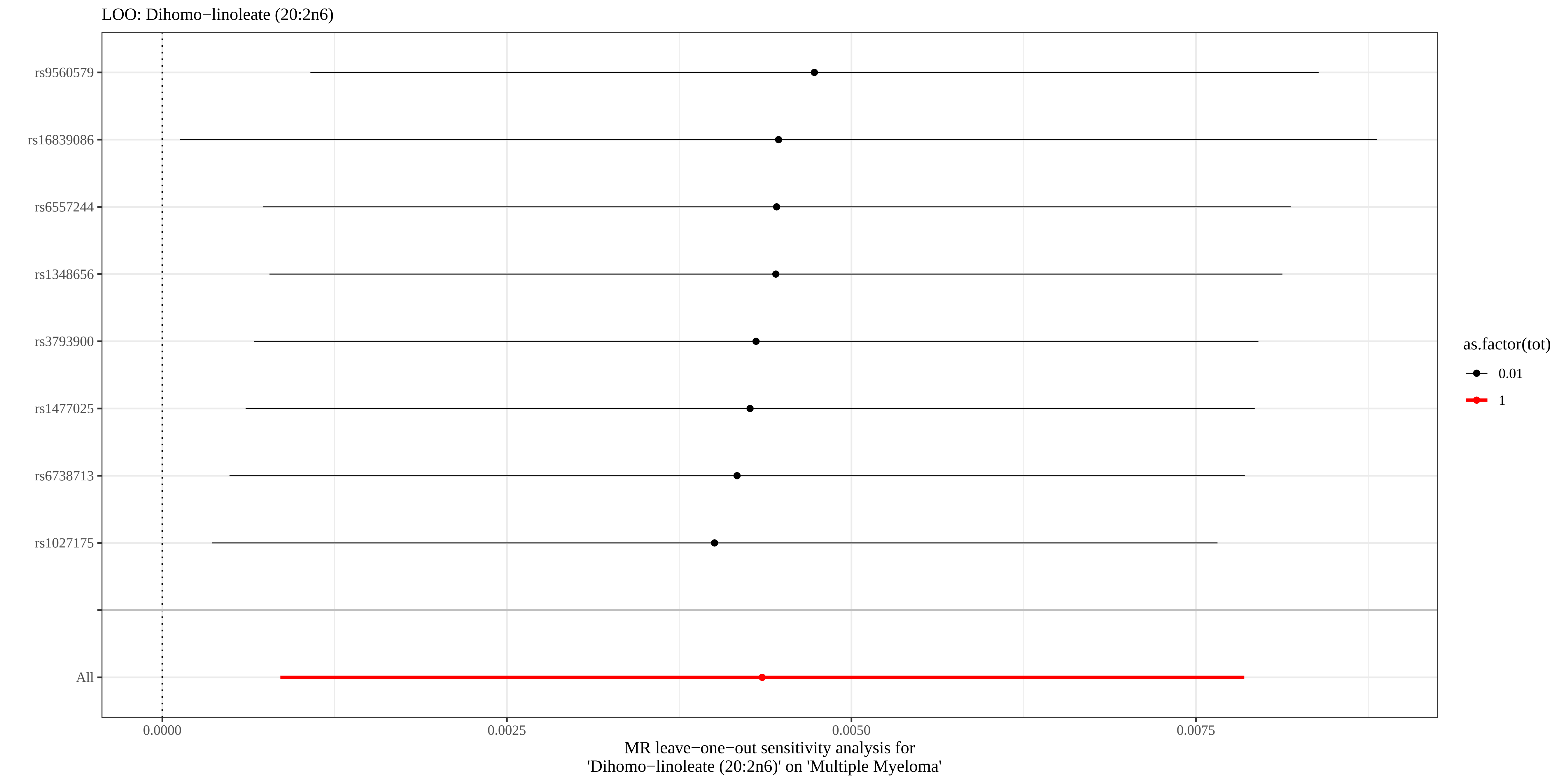

LOO: Dihomo−linoleate (20:2n6)
rs9560579
rs16839086
rs6557244
rs1348656
rs3793900
rs1477025
rs6738713
rs1027175
All
0.0000
0.0025
0.0050
0.0075
MR leave−one−out sensitivity analysis for
'Dihomo−linoleate (20:2n6)' on 'Multiple Myeloma'
as.factor(tot)
0.01
1

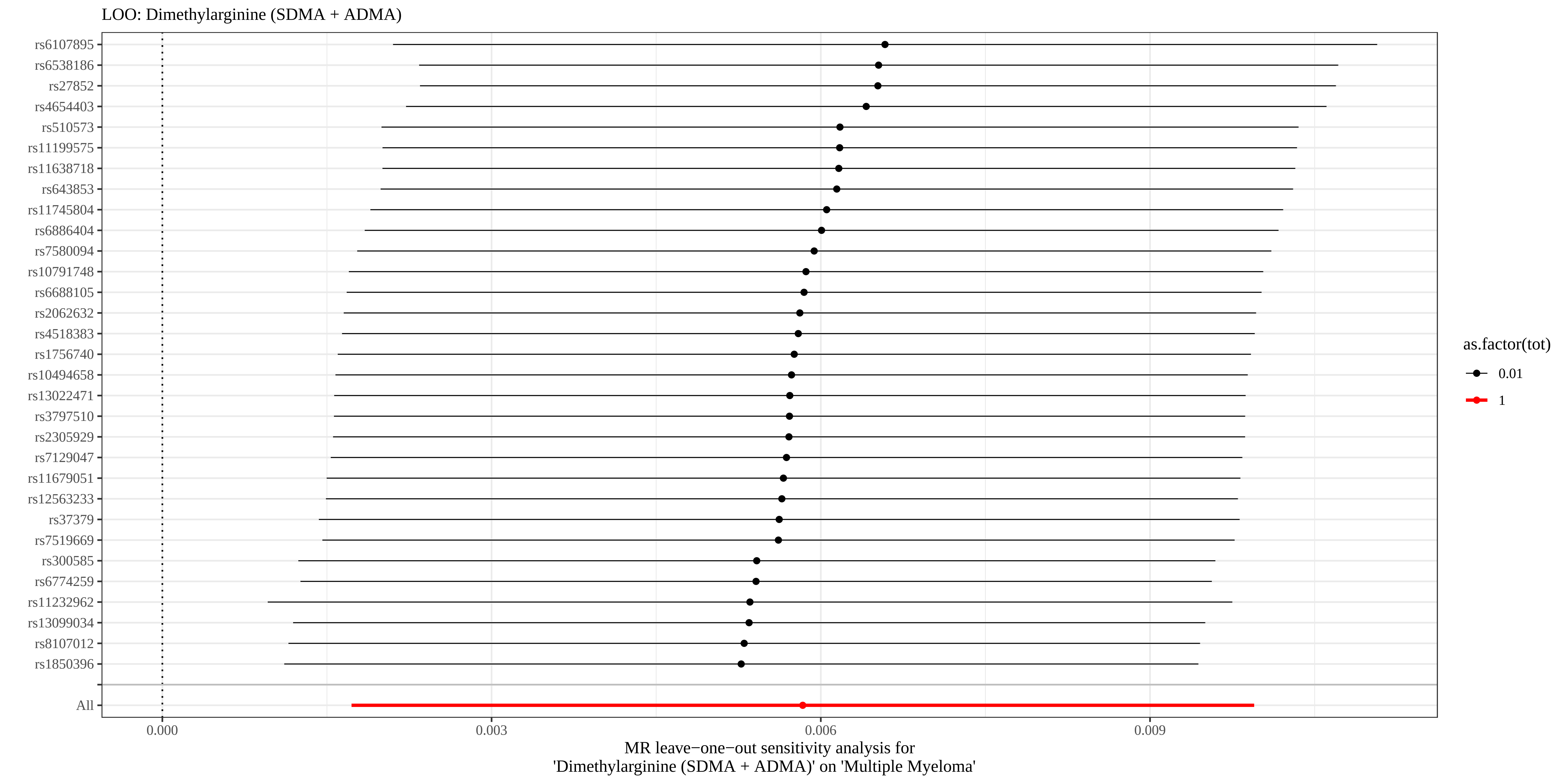

LOO: Dimethylarginine (SDMA + ADMA)
rs6107895
rs6538186
rs27852
rs4654403
rs510573
rs11199575
rs11638718
rs643853
rs11745804
rs6886404
rs7580094
rs10791748
rs6688105
rs2062632
rs4518383
rs1756740
rs10494658
rs13022471
rs3797510
rs2305929
rs7129047
rs11679051
rs12563233
rs37379
rs7519669
rs300585
rs6774259
rs11232962
rs13099034
rs8107012
rs1850396
All
0.000
0.003
0.006
0.009
MR leave−one−out sensitivity analysis for
'Dimethylarginine (SDMA + ADMA)' on 'Multiple Myeloma'
as.factor(tot)
0.01
1

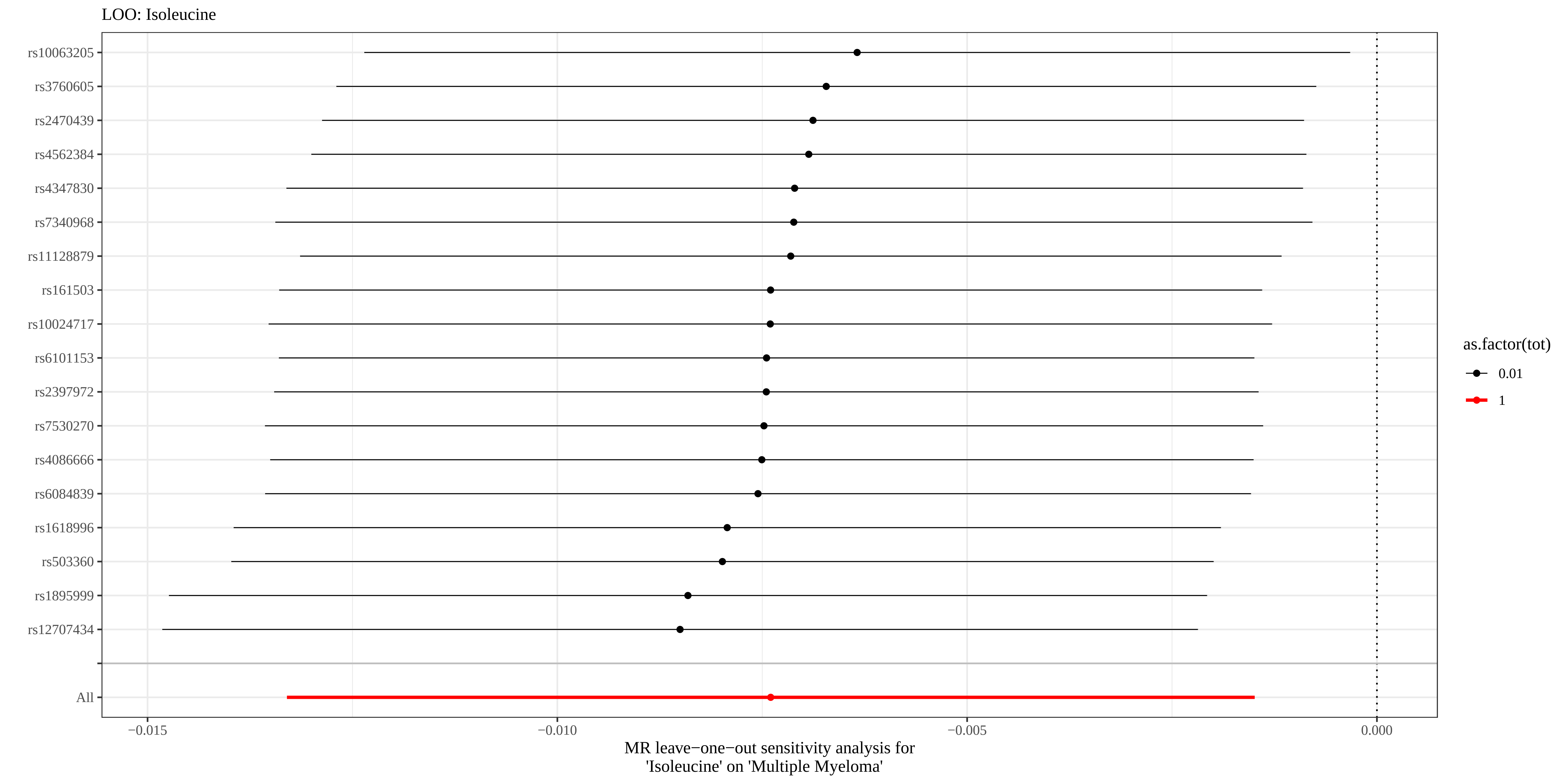

LOO: Isoleucine
rs10063205
rs3760605
rs2470439
rs4562384
rs4347830
rs7340968
rs11128879
rs161503
rs10024717
rs6101153
rs2397972
rs7530270
rs4086666
rs6084839
rs1618996
rs503360
rs1895999
rs12707434
All
−0.015
−0.010
−0.005
0.000
MR leave−one−out sensitivity analysis for
'Isoleucine' on 'Multiple Myeloma'
as.factor(tot)
0.01
1

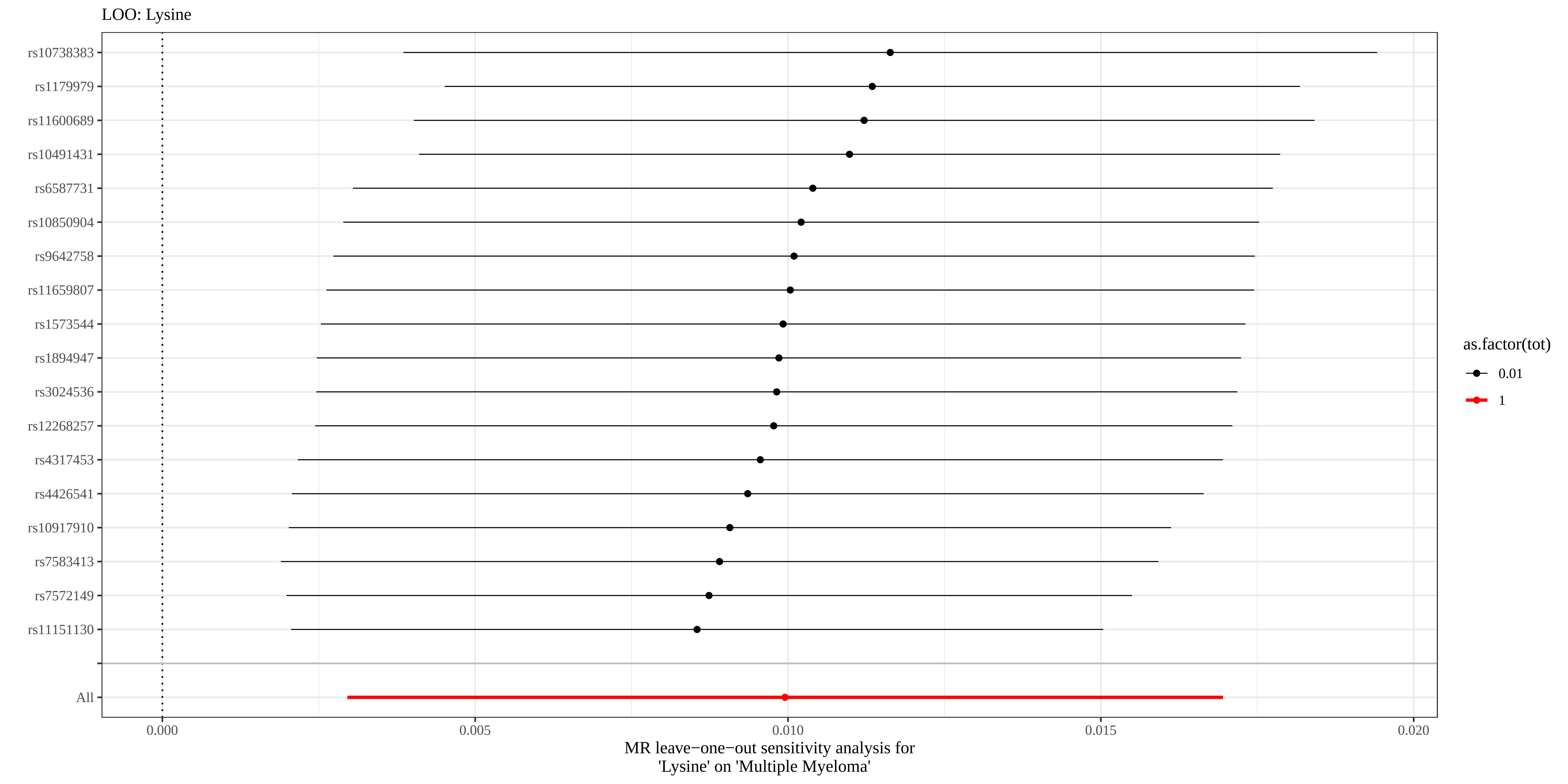
LOO: Lysine
rs10738383
rs1179979
rs11600689
rs10491431
rs6587731
rs10850904
rs9642758
rs11659807
rs1573544
rs1894947
rs3024536
rs12268257
rs4317453
rs4426541
rs10917910
rs7583413
rs7572149
rs11151130
All
0.000
0.005
0.010
0.015
0.020
MR leave−one−out sensitivity analysis for
'Lysine' on 'Multiple Myeloma'
as.factor(tot)
0.01
1

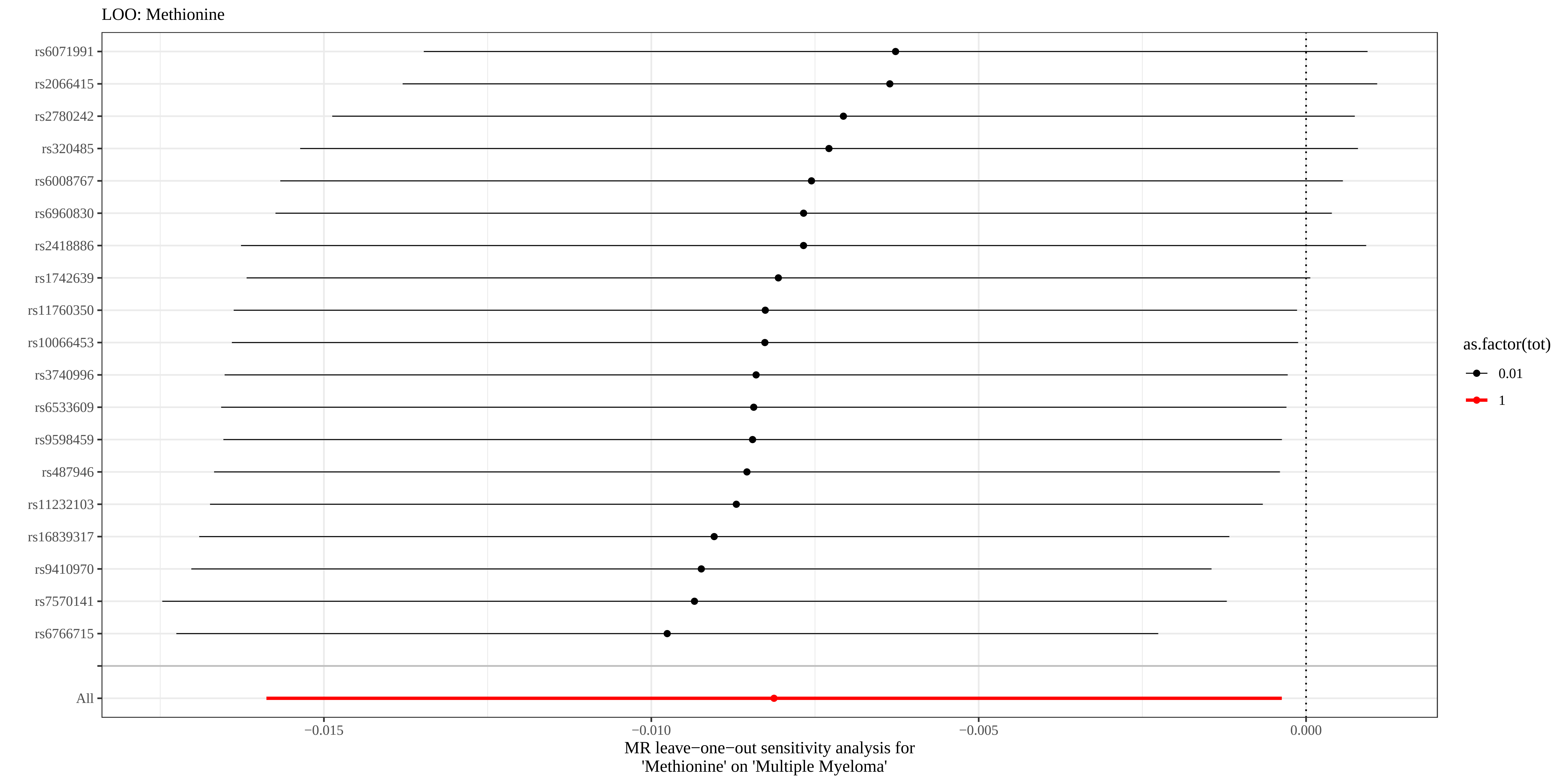
LOO: Methionine
rs6071991
rs2066415
rs2780242
rs320485
rs6008767
rs6960830
rs2418886
rs1742639
rs11760350
rs10066453
rs3740996
rs6533609
rs9598459
rs487946
rs11232103
rs16839317
rs9410970
rs7570141
rs6766715
All
−0.015
−0.010
−0.005
0.000
MR leave−one−out sensitivity analysis for
'Methionine' on 'Multiple Myeloma'
as.factor(tot)
0.01
1

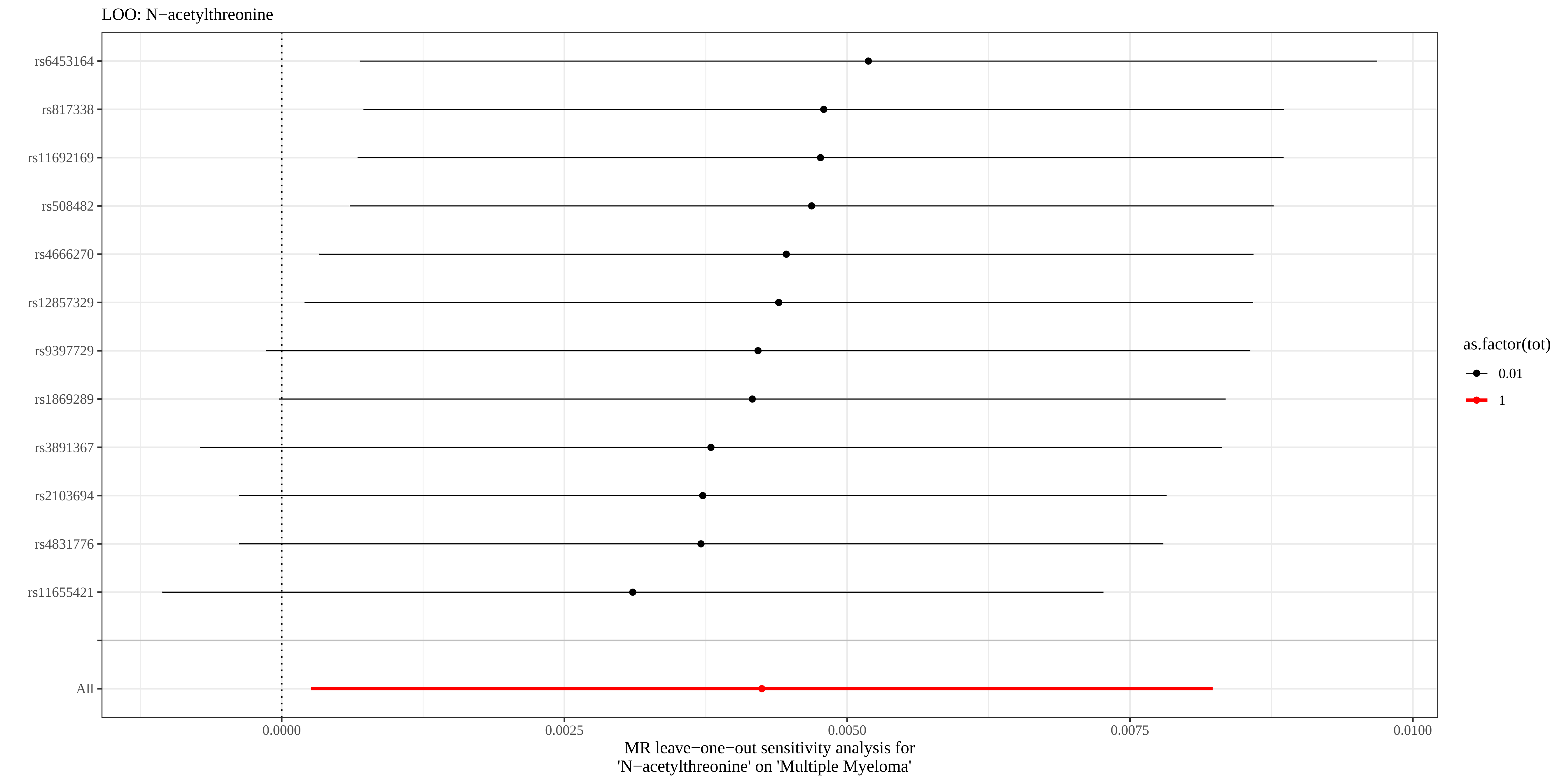

LOO: N−acetylthreonine
rs6453164
rs817338
rs11692169
rs508482
rs4666270
rs12857329
rs9397729
rs1869289
rs3891367
rs2103694
rs4831776
rs11655421
All
0.0000
0.0025
0.0050
0.0075
0.0100
MR leave−one−out sensitivity analysis for
'N−acetylthreonine' on 'Multiple Myeloma'
as.factor(tot)
0.01
1

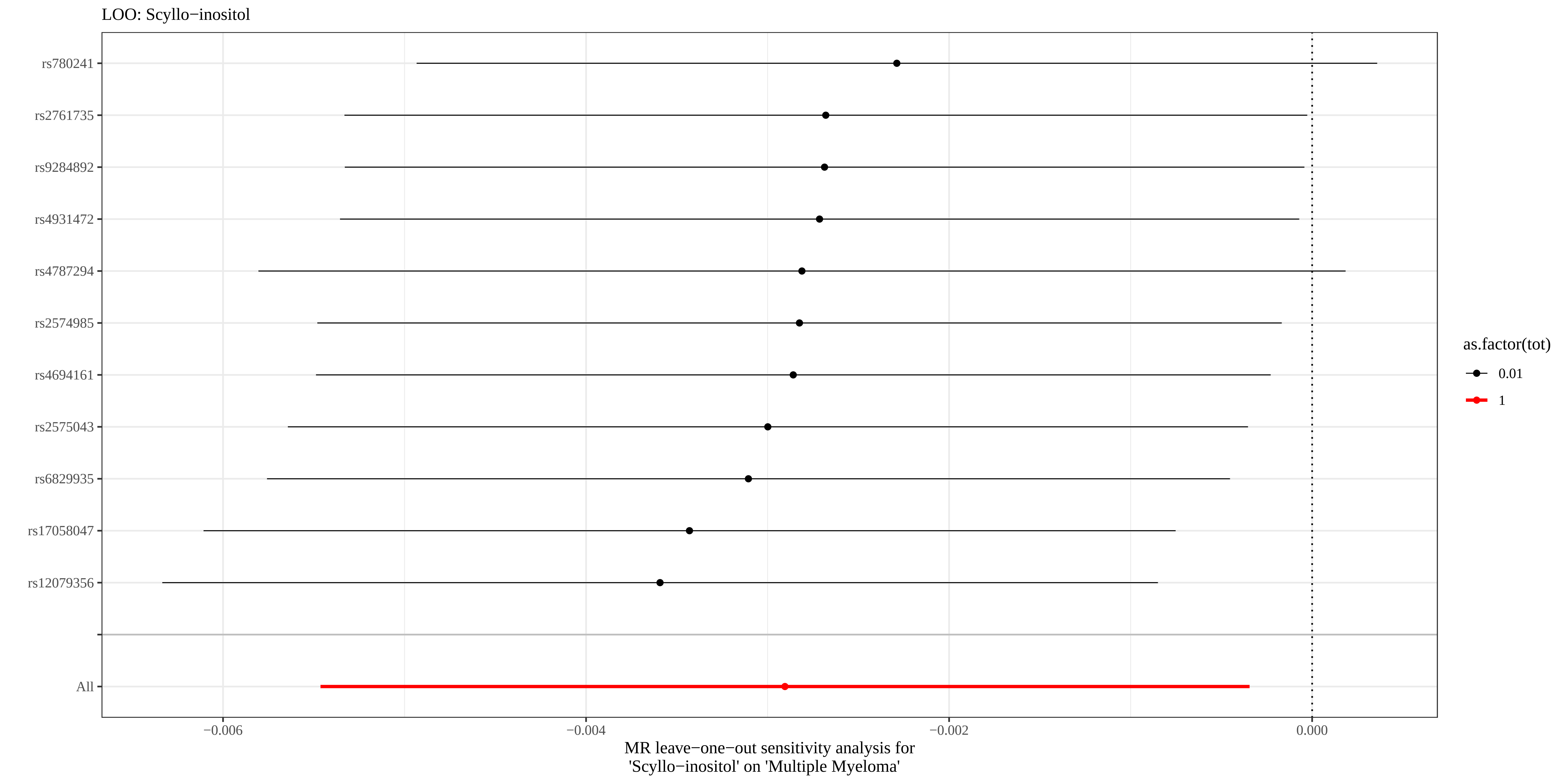

LOO: Scyllo−inositol
rs780241
rs2761735
rs9284892
rs4931472
rs4787294
rs2574985
rs4694161
rs2575043
rs6829935
rs17058047
rs12079356
All
−0.006
−0.004
−0.002
0.000
MR leave−one−out sensitivity analysis for
'Scyllo−inositol' on 'Multiple Myeloma'
as.factor(tot)
0.01
1

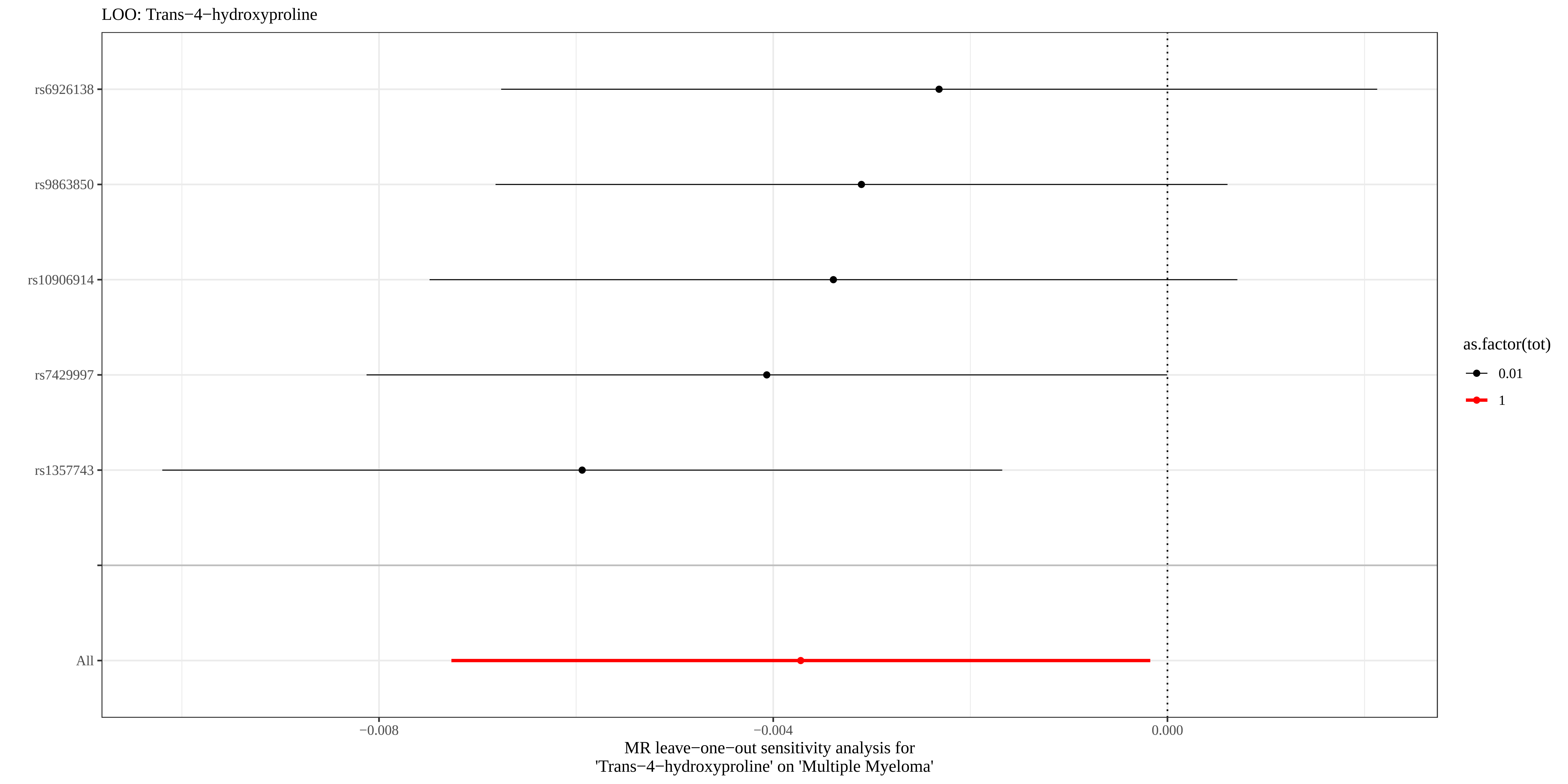

LOO: Trans−4−hydroxyproline
rs6926138
rs9863850
rs10906914
rs7429997
rs1357743
All
−0.008
−0.004
0.000
MR leave−one−out sensitivity analysis for
'Trans−4−hydroxyproline' on 'Multiple Myeloma'
as.factor(tot)
0.01
1

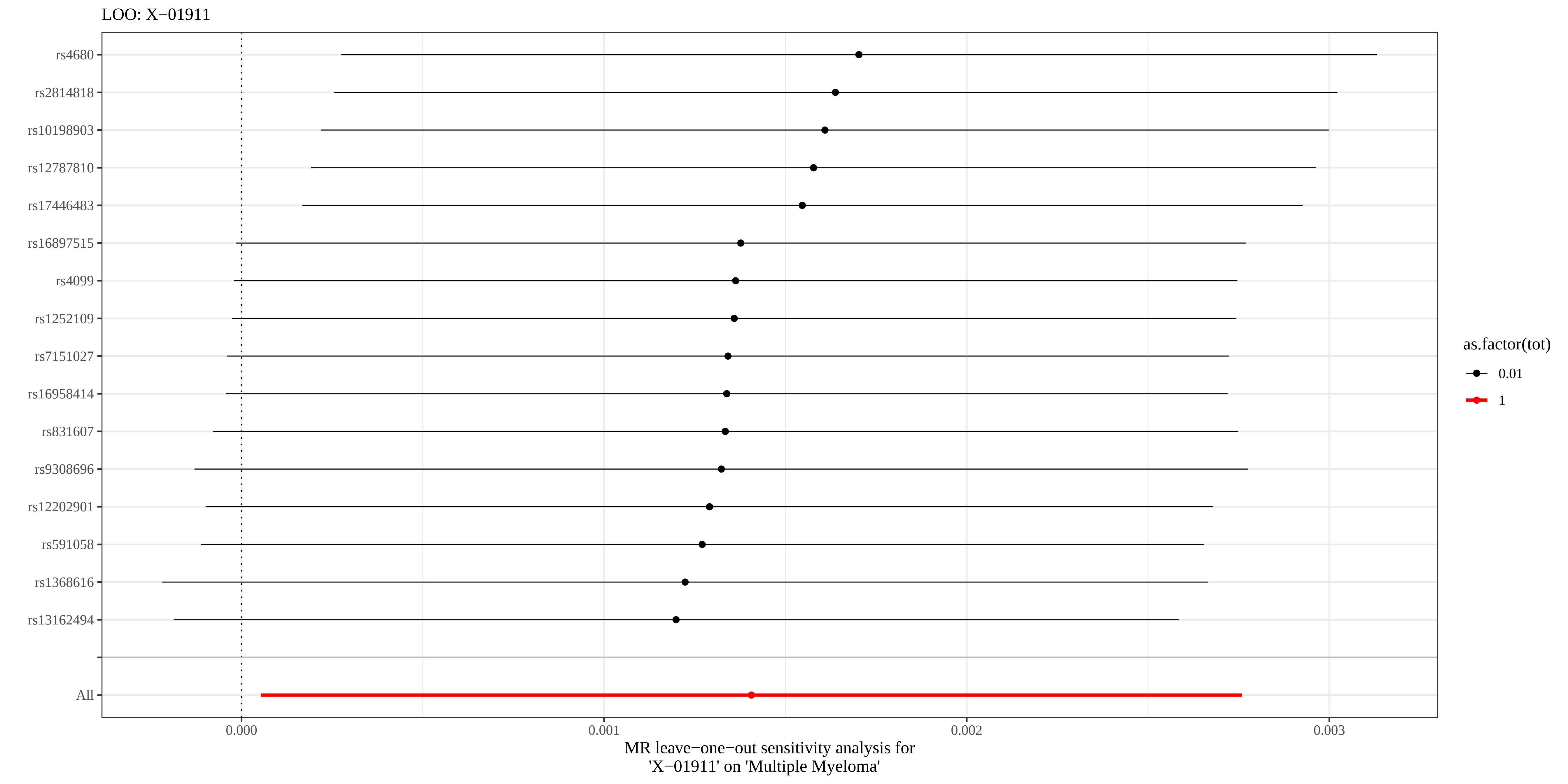

LOO: X−01911
rs4680
rs2814818
rs10198903
rs12787810
rs17446483
rs16897515
rs4099
rs1252109
rs7151027
rs16958414
rs831607
rs9308696
rs12202901
rs591058
rs1368616
rs13162494
All
0.000
0.001
0.002
0.003
MR leave−one−out sensitivity analysis for
'X−01911' on 'Multiple Myeloma'
as.factor(tot)
0.01
1

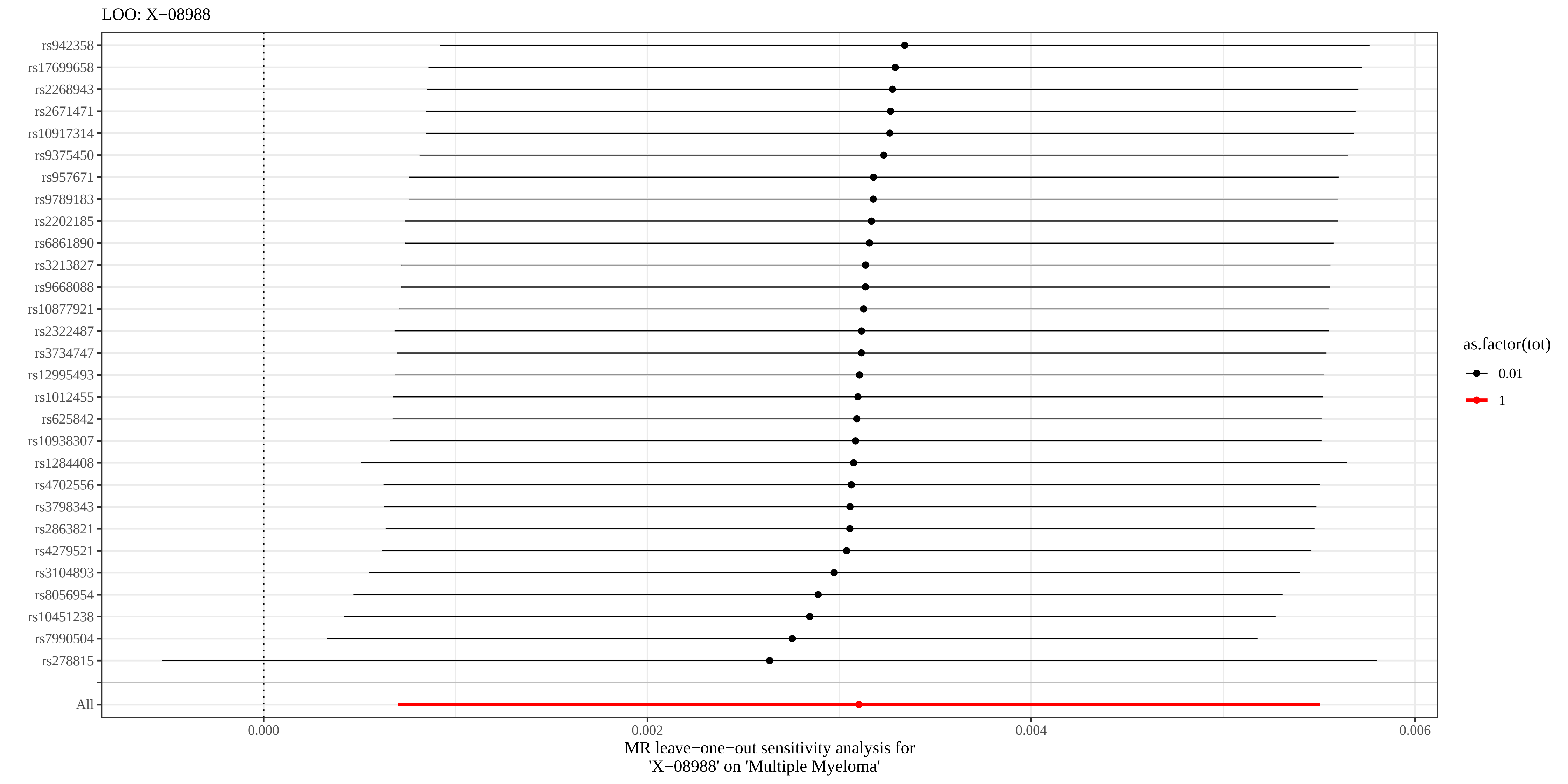

LOO: X−08988
rs942358
rs17699658
rs2268943
rs2671471
rs10917314
rs9375450
rs957671
rs9789183
rs2202185
rs6861890
rs3213827
rs9668088
rs10877921
rs2322487
rs3734747
rs12995493
rs1012455
rs625842
rs10938307
rs1284408
rs4702556
rs3798343
rs2863821
rs4279521
rs3104893
rs8056954
rs10451238
rs7990504
rs278815
All
0.000
0.002
0.004
0.006
MR leave−one−out sensitivity analysis for
'X−08988' on 'Multiple Myeloma'
as.factor(tot)
0.01
1

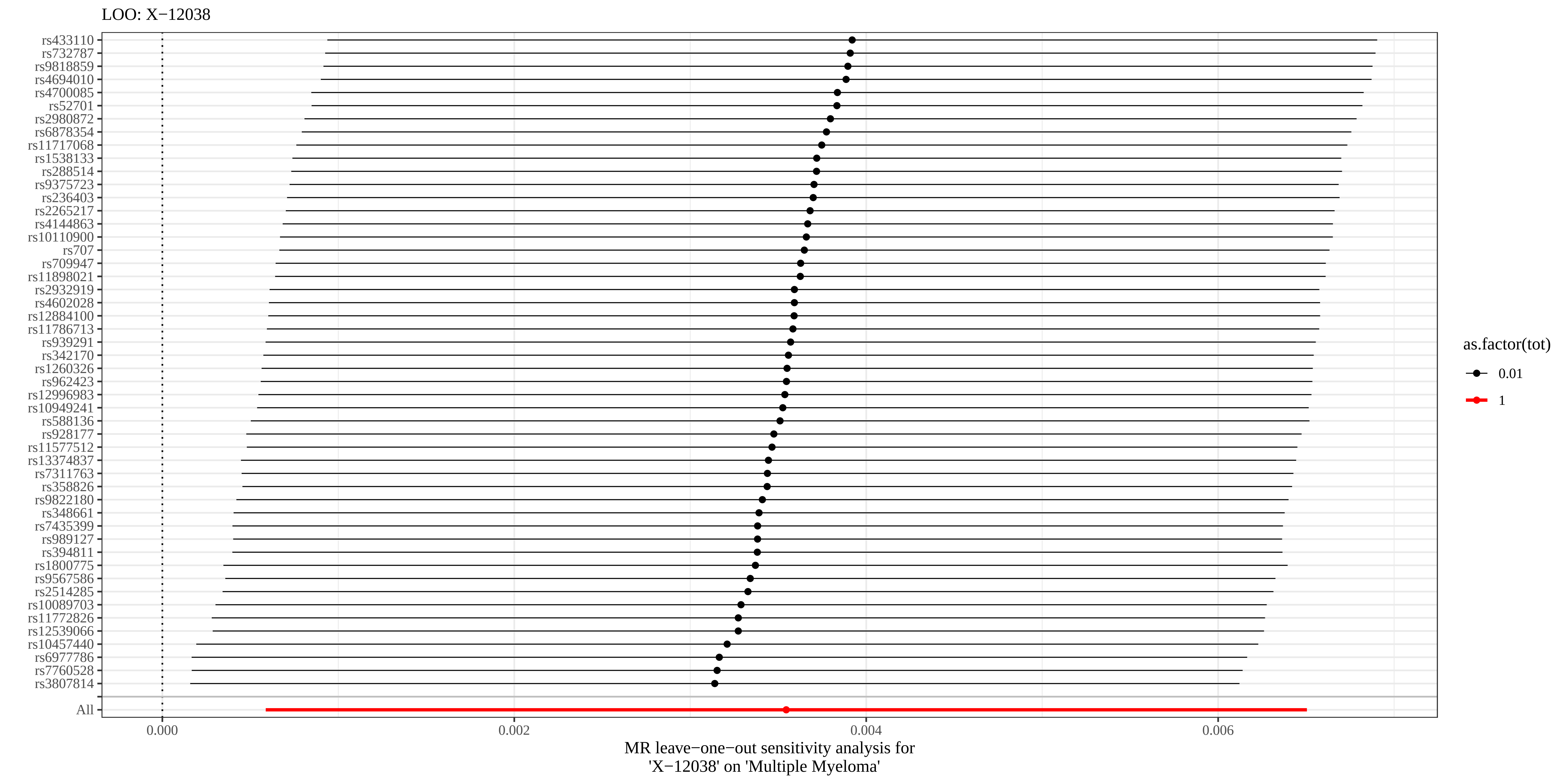
LOO: X−12038
rs433110
rs732787
rs9818859
rs4694010
rs4700085
rs52701
rs2980872
rs6878354
rs11717068
rs1538133
rs288514
rs9375723
rs236403
rs2265217
rs4144863
rs10110900
rs707
rs709947
rs11898021
rs2932919
rs4602028
rs12884100
rs11786713
rs939291
rs342170
rs1260326
rs962423
rs12996983
rs10949241
rs588136
rs928177
rs11577512
rs13374837
rs7311763
rs358826
rs9822180
rs348661
rs7435399
rs989127
rs394811
rs1800775
rs9567586
rs2514285
rs10089703
rs11772826
rs12539066
rs10457440
rs6977786
rs7760528
rs3807814
All
0.000
0.002
0.004
0.006
MR leave−one−out sensitivity analysis for
'X−12038' on 'Multiple Myeloma'
as.factor(tot)
0.01
1

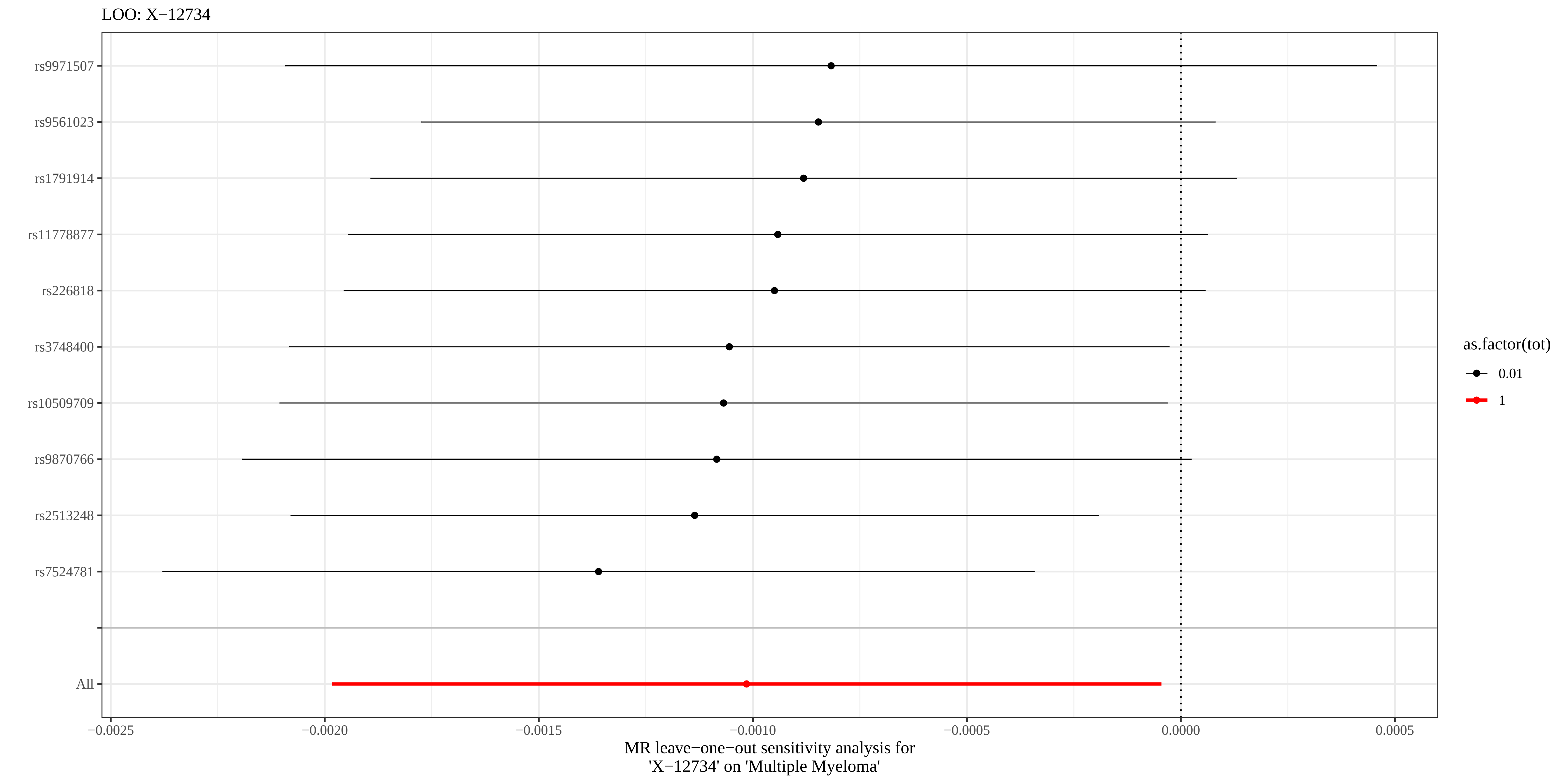

LOO: X−12734
rs9971507
rs9561023
rs1791914
rs11778877
rs226818
rs3748400
rs10509709
rs9870766
rs2513248
rs7524781
All
−0.0025
−0.0020
−0.0015
−0.0010
−0.0005
0.0000
0.0005
MR leave−one−out sensitivity analysis for
'X−12734' on 'Multiple Myeloma'
as.factor(tot)
0.01
1

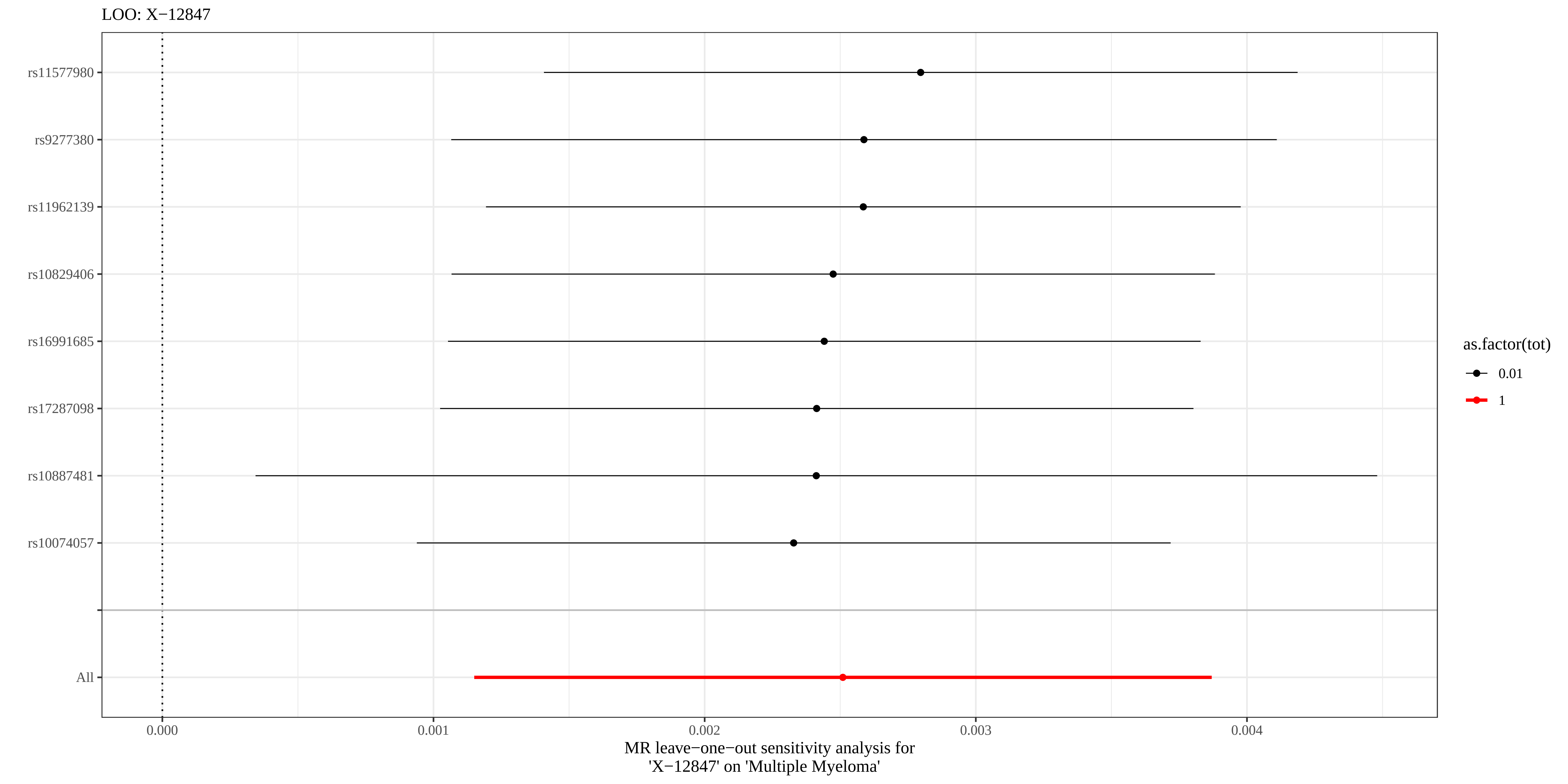

LOO: X−12847
rs11577980
rs9277380
rs11962139
rs10829406
rs16991685
rs17287098
rs10887481
rs10074057
All
0.000
0.001
0.002
0.003
0.004
MR leave−one−out sensitivity analysis for
'X−12847' on 'Multiple Myeloma'
as.factor(tot)
0.01
1

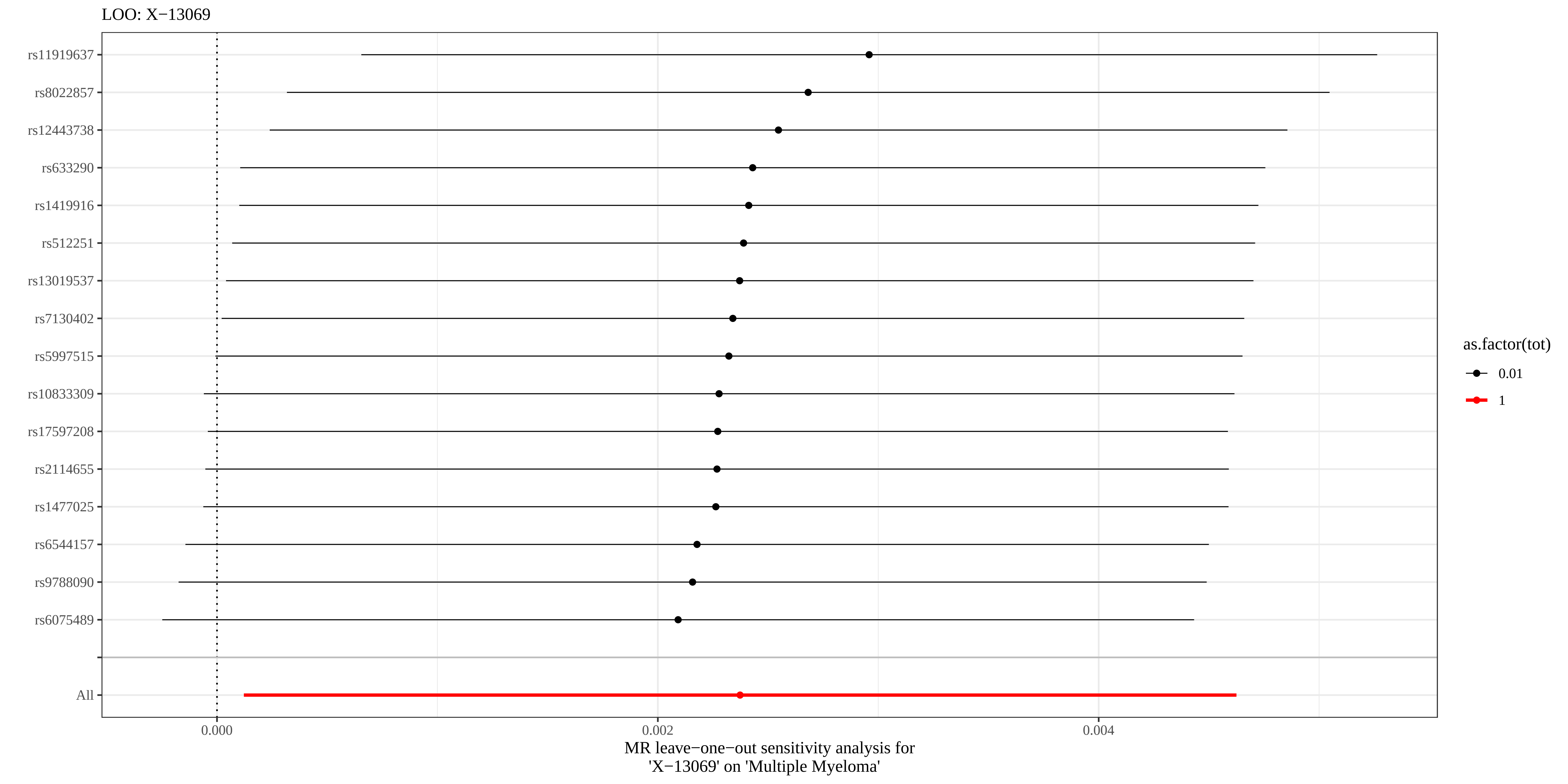

LOO: X−13069
rs11919637
rs8022857
rs12443738
rs633290
rs1419916
rs512251
rs13019537
rs7130402
rs5997515
rs10833309
rs17597208
rs2114655
rs1477025
rs6544157
rs9788090
rs6075489
All
0.000
0.002
0.004
MR leave−one−out sensitivity analysis for
'X−13069' on 'Multiple Myeloma'
as.factor(tot)
0.01
1

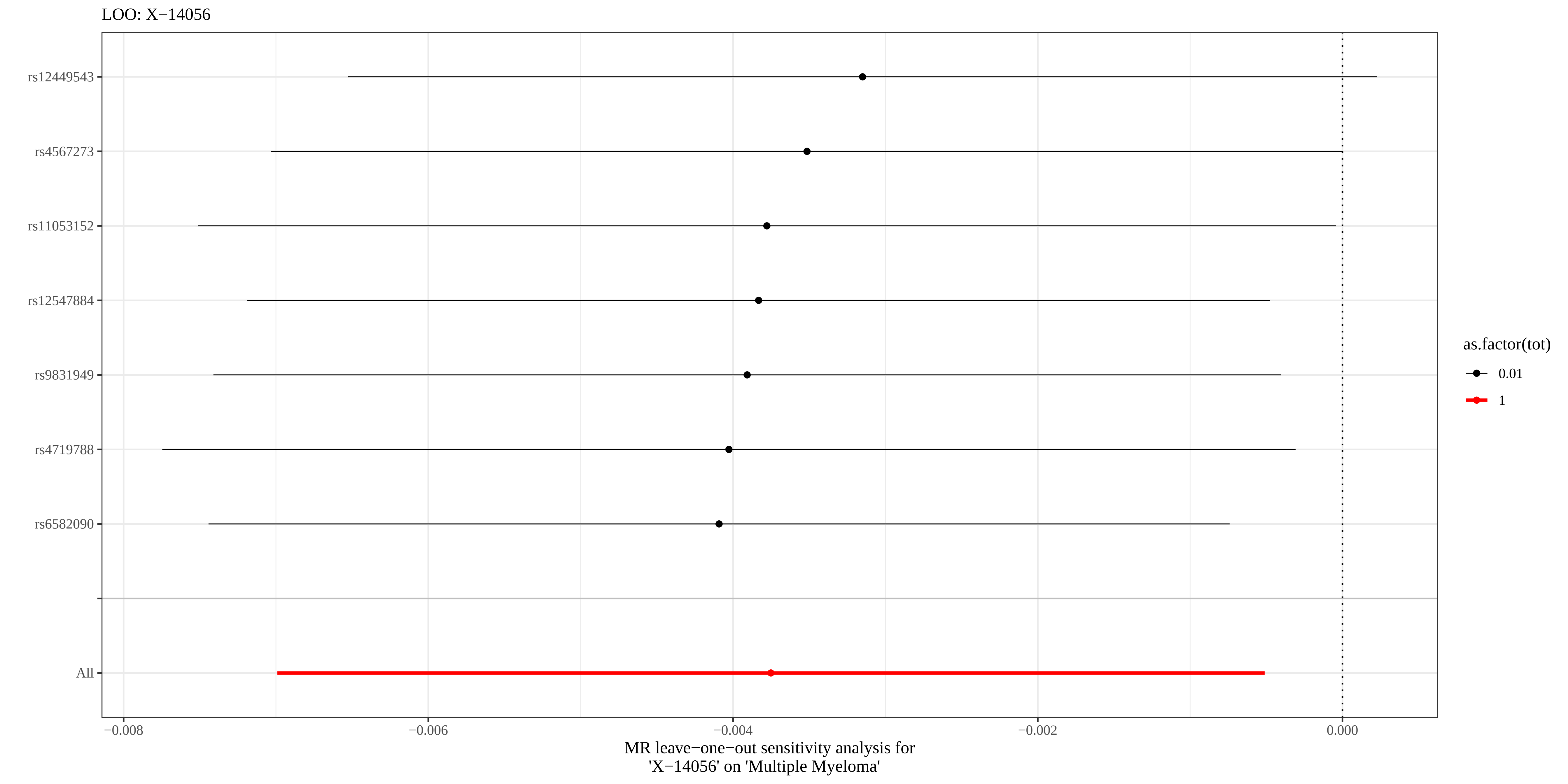
LOO: X−14056
rs12449543
rs4567273
rs11053152
rs12547884
rs9831949
rs4719788
rs6582090
All
−0.008
−0.006
−0.004
−0.002
0.000
MR leave−one−out sensitivity analysis for
'X−14056' on 'Multiple Myeloma'
as.factor(tot)
0.01
1
