# Supplementary material for: Integrating Bidirectional Mendelian Randomization with Multi-Omics Reveals Causal Serum Metabolites and Novel Metabolic Drivers of Multiple Myeloma
Source: Int J Mol Sci. 2026 Feb 16;27(4):1904. doi: 10.3390/ijms27041904 (PMC12941277; doi:10.3390/ijms27041904)
Supplement: Supplementary file 1 [file ijms-27-01904-s001.zip › Figure_S3.pdf]

Supplementary Figure S3. Scatter plots of the genetic association of remaining 19 on MM risk.

Scatter: 1−docosahexaenoylglycerophosphocholine*

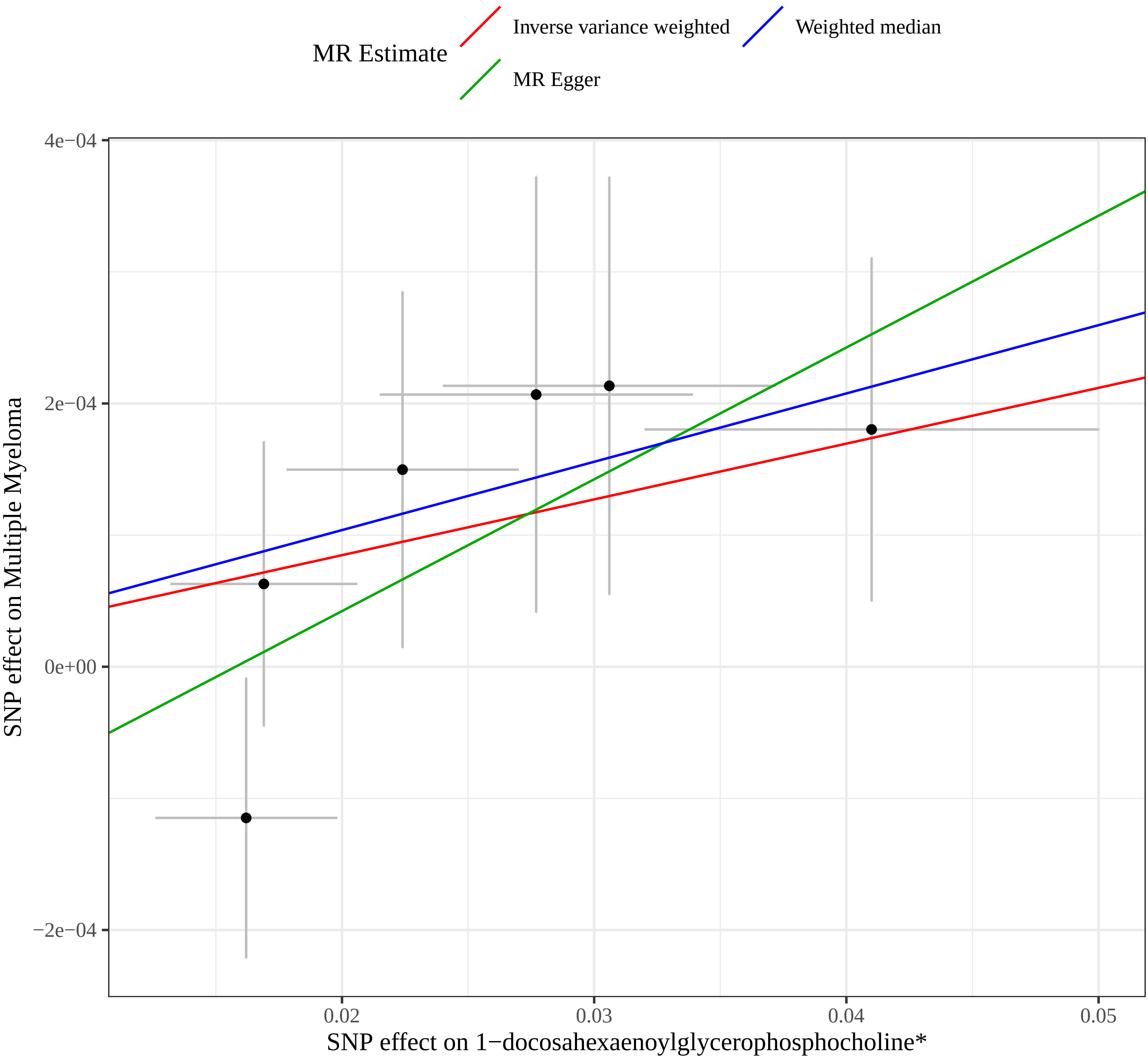

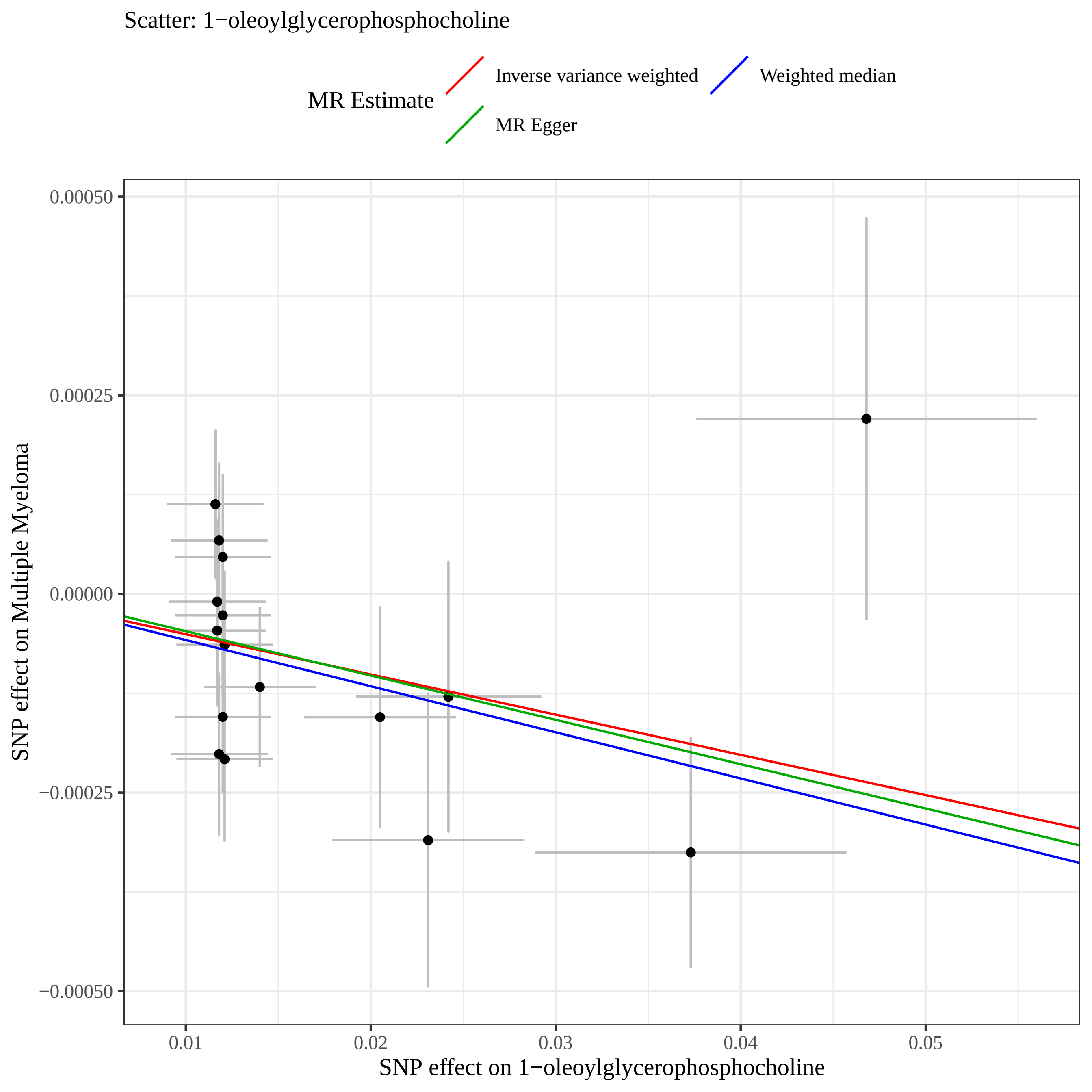

Scatter: 1−oleoylglycerophosphocholine
MR Estimate
Inverse variance weighted
Weighted median
MR Egger
0.00050
0.00025
0.00000
−0.00025
−0.00050
SNP effect on Multiple Myeloma
0.01
0.02
0.03
0.04
0.05
SNP effect on 1−oleoylglycerophosphocholine

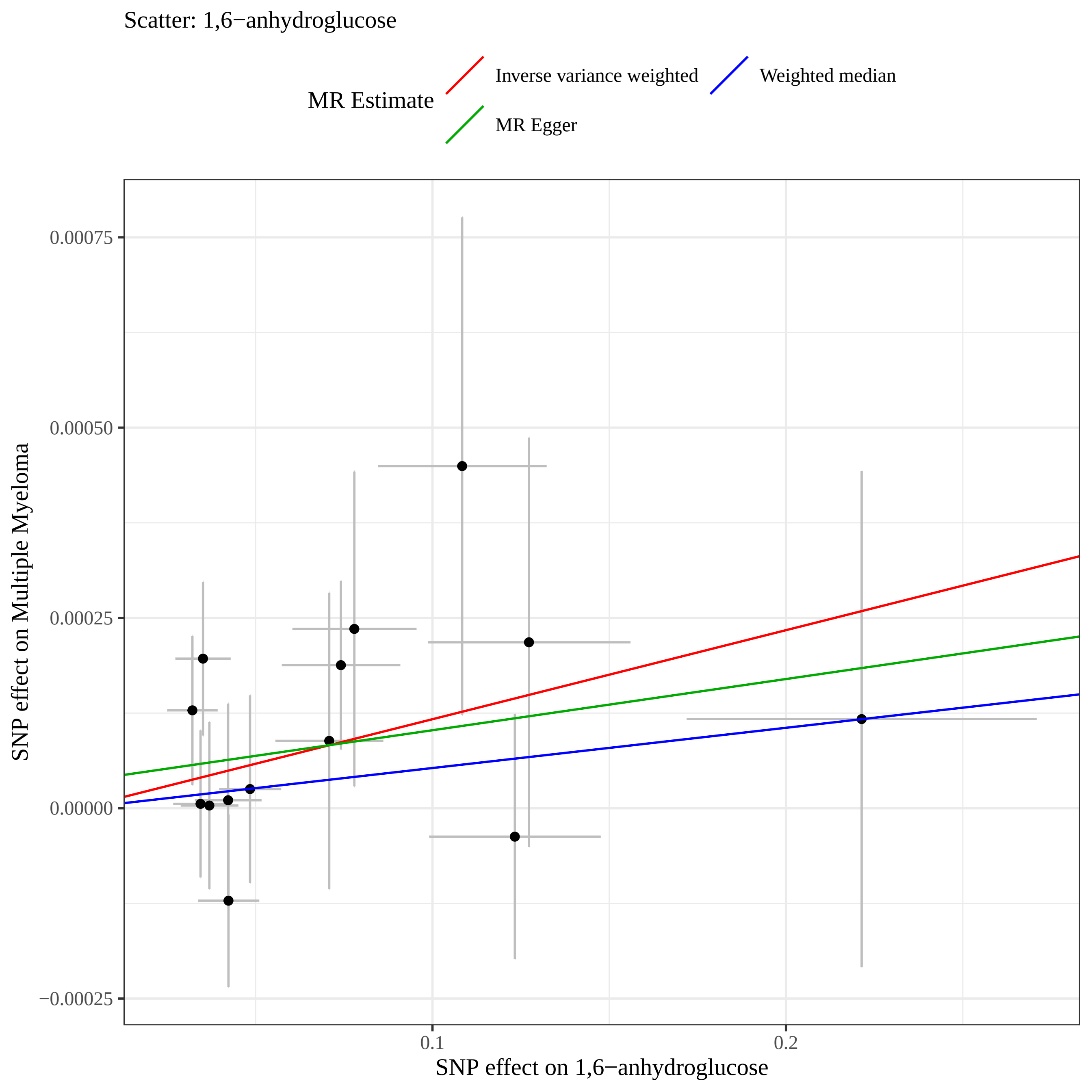

Scatter: 1,6−anhydroglucose
MR Estimate
Inverse variance weighted
MR Egger
Weighted median
0.00075
0.00050
0.00025
0.00000
−0.00025
0.1
0.2
SNP effect on 1,6−anhydroglucose
SNP effect on Multiple Myeloma

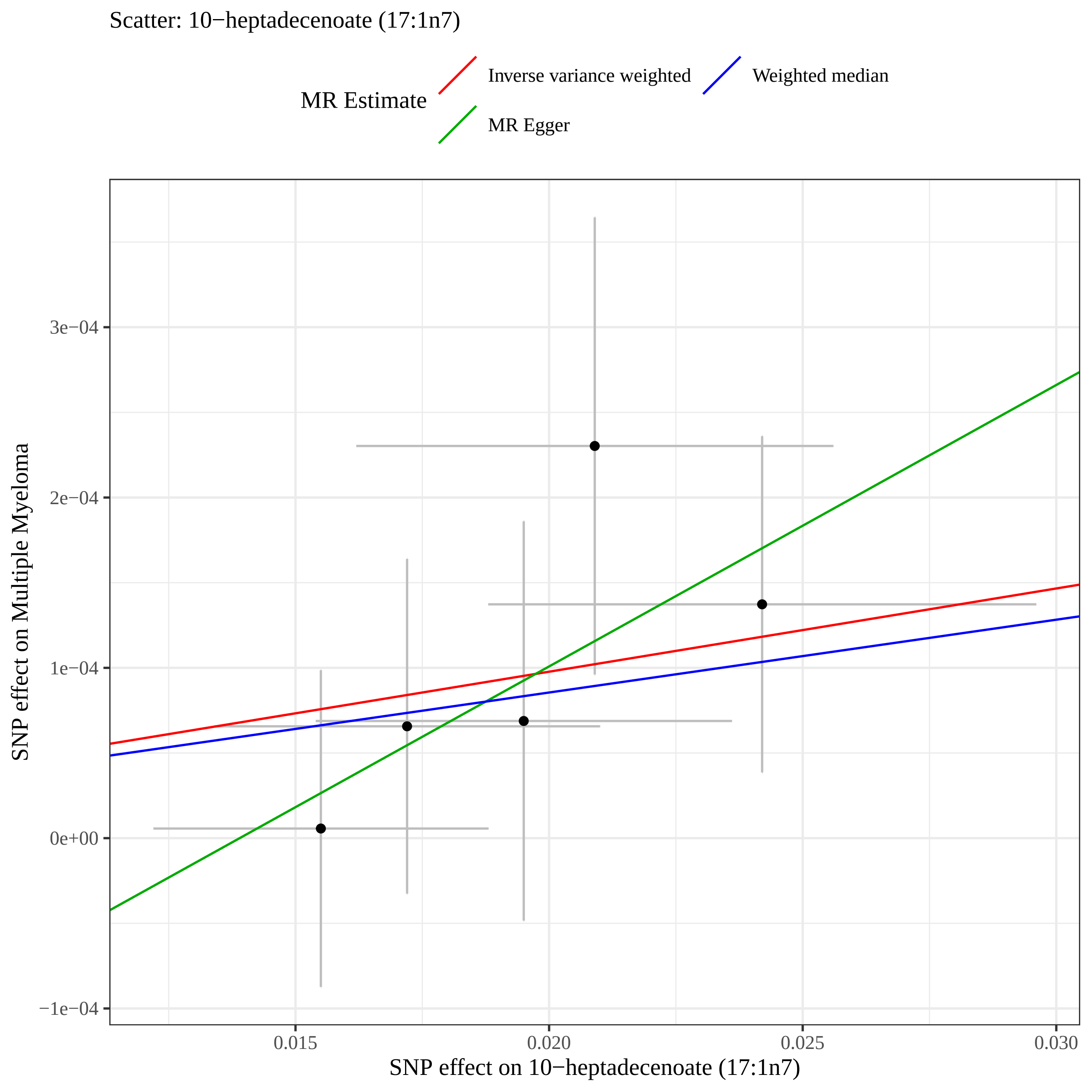

Scatter: 10−heptadecenoate (17:1n7)
MR Estimate
Inverse variance weighted
Weighted median
MR Egger
3e−04
2e−04
1e−04
0e+00
−1e−04
0.015
0.020
0.025
0.030
SNP effect on Multiple Myeloma
SNP effect on 10−heptadecenoate (17:1n7)

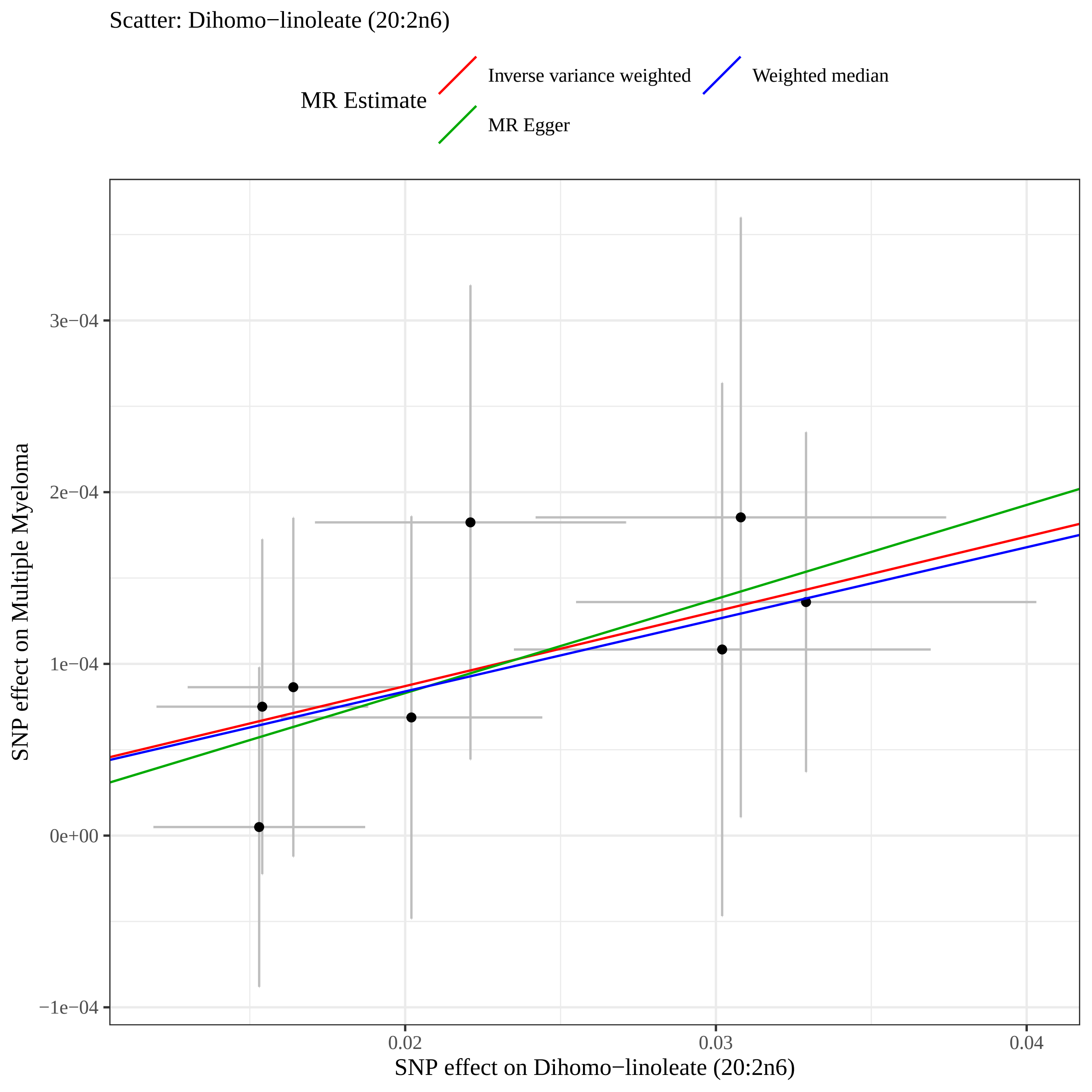

Scatter: Dihomo−linoleate (20:2n6)
MR Estimate
Inverse variance weighted
Weighted median
MR Egger
SNP effect on Multiple Myeloma
3e−04
2e−04
1e−04
0e+00
−1e−04
0.02
0.03
0.04
SNP effect on Dihomo−linoleate (20:2n6)

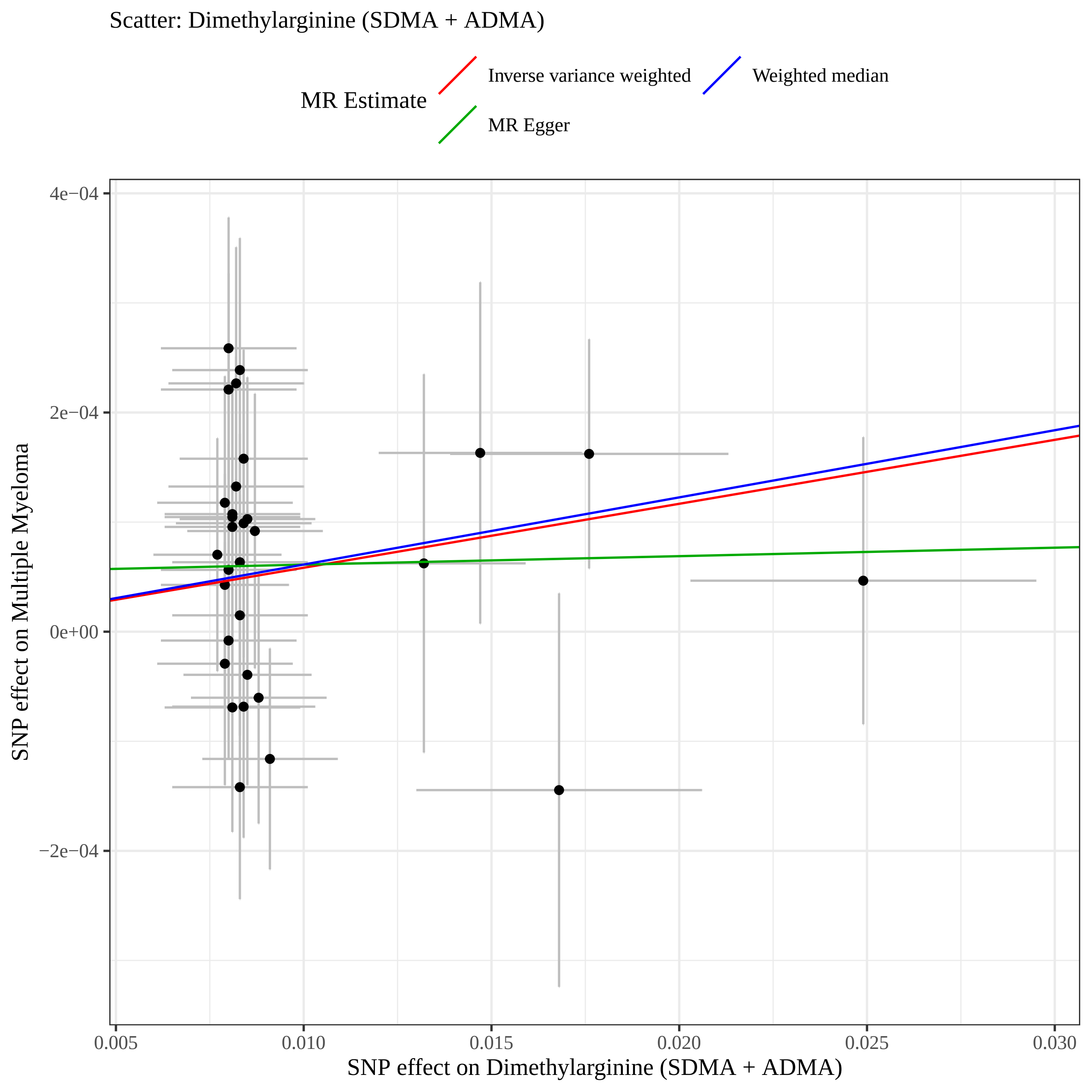

Scatter: Dimethylarginine (SDMA + ADMA)
MR Estimate
Inverse variance weighted
Weighted median
MR Egger
4e−04
2e−04
0e+00
−2e−04
SNP effect on Multiple Myeloma
0.005
0.010
0.015
0.020
0.025
0.030
SNP effect on Dimethylarginine (SDMA + ADMA)

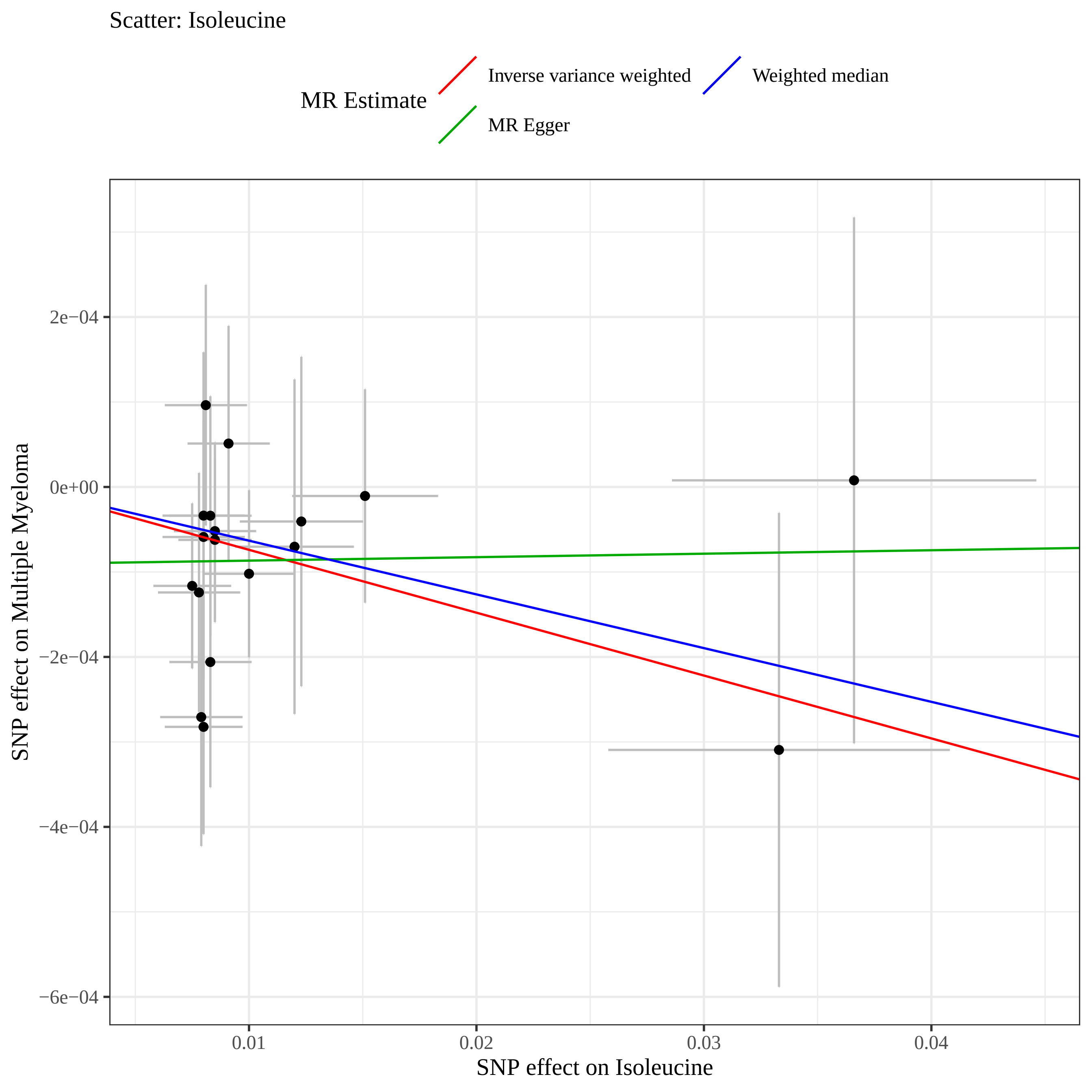

Scatter: Isoleucine
MR Estimate
Inverse variance weighted
Weighted median
MR Egger
SNP effect on Multiple Myeloma
2e−04
0e+00
−2e−04
−4e−04
−6e−04
0.01
0.02
0.03
0.04
SNP effect on Isoleucine

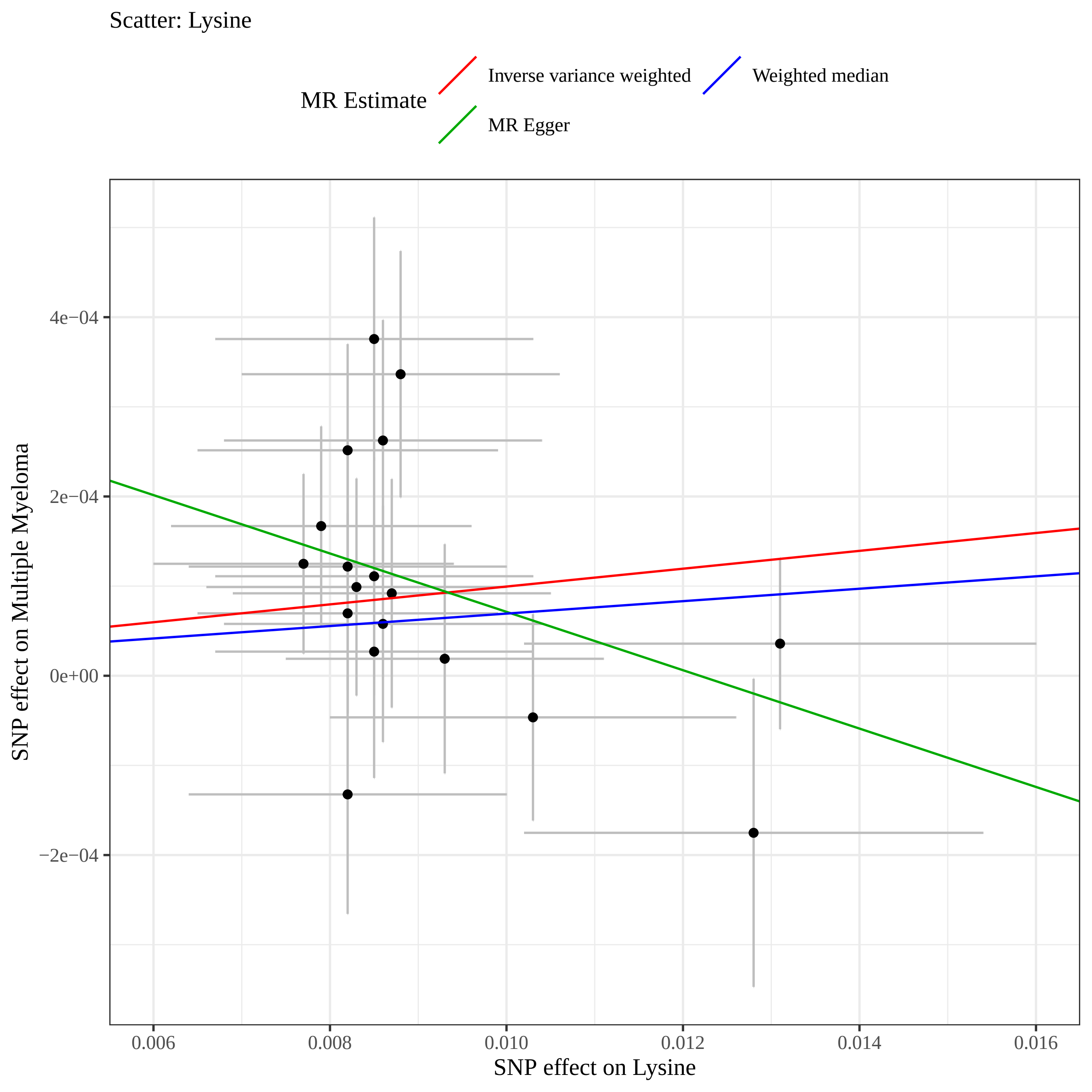

Scatter: Lysine
MR Estimate
Inverse variance weighted
Weighted median
MR Egger
SNP effect on Multiple Myeloma
4e−04
2e−04
0e+00
−2e−04
0.006
0.008
0.010
0.012
0.014
0.016
SNP effect on Lysine

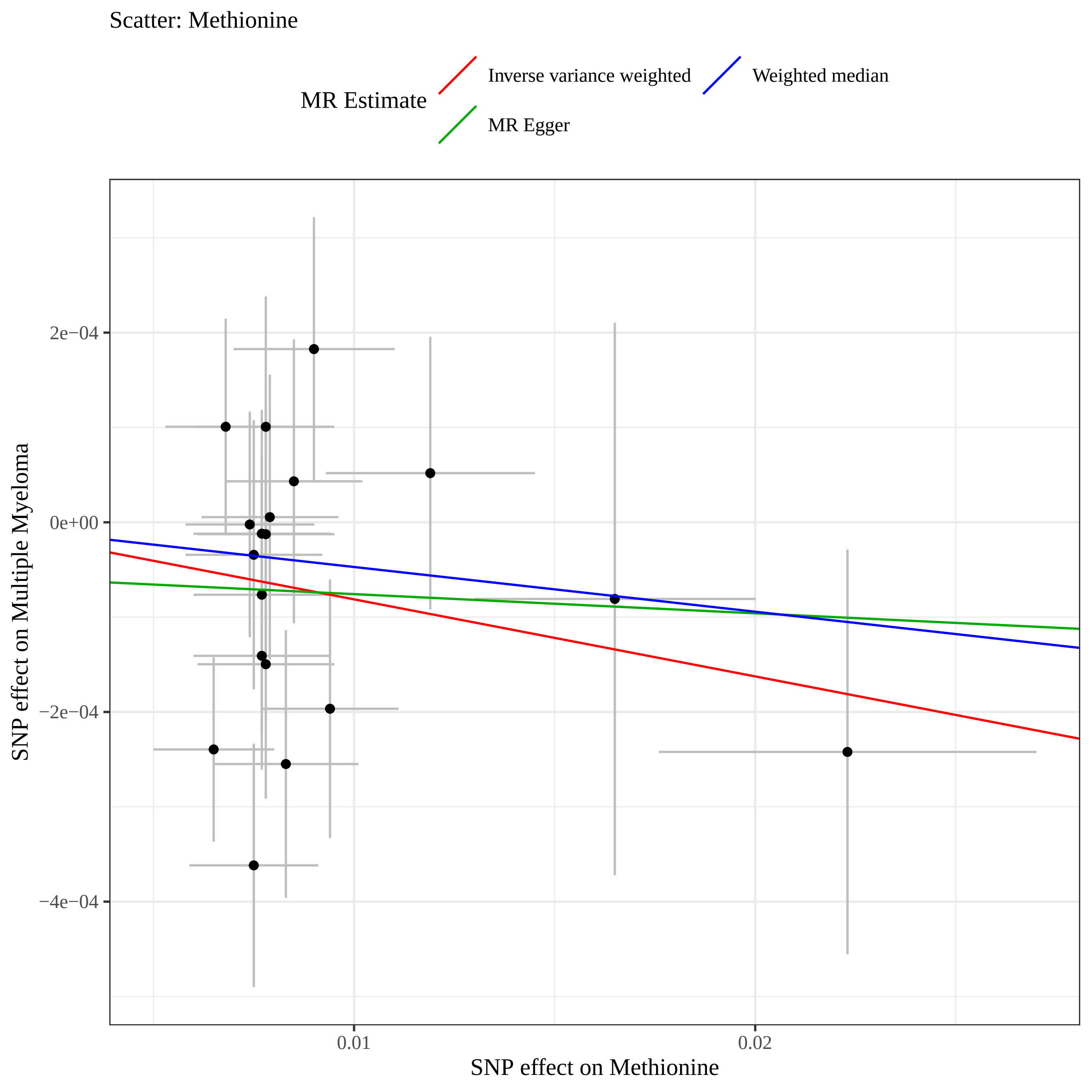

Scatter: Methionine
MR Estimate
Inverse variance weighted
Weighted median
MR Egger
2e−04
0e+00
−2e−04
−4e−04
SNP effect on Multiple Myeloma
0.01
0.02
SNP effect on Methionine

Scatter: N−acetylthreonine

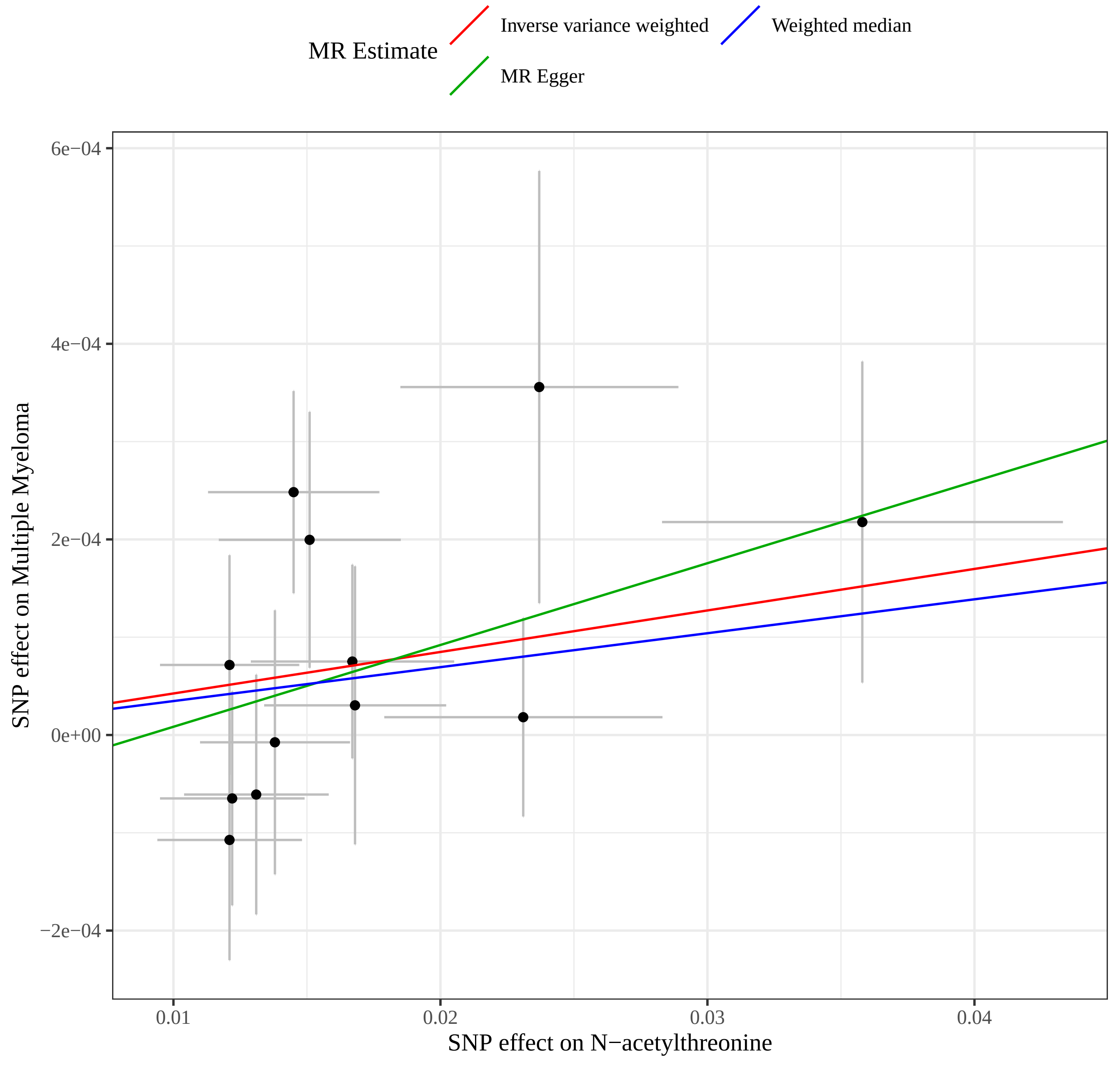

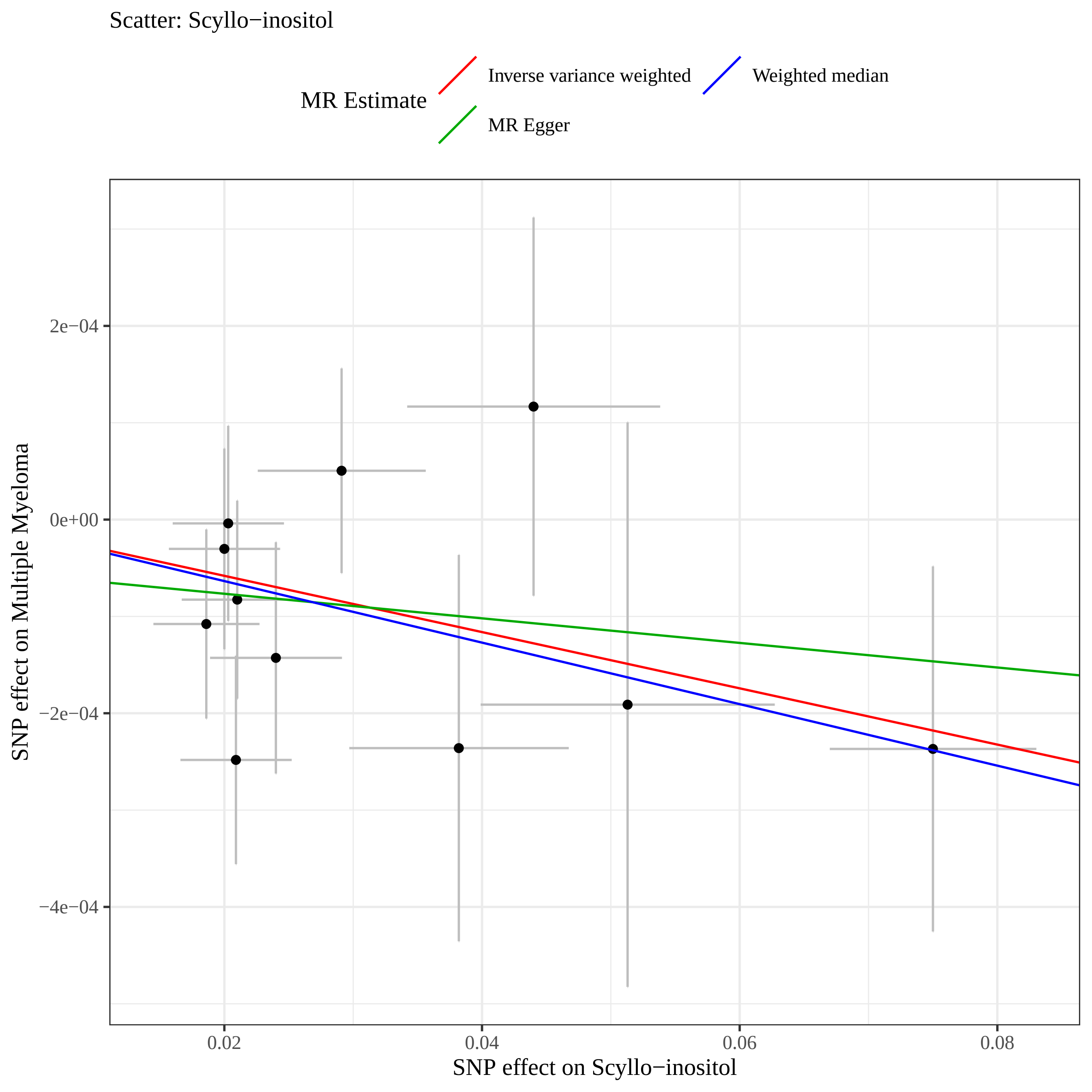

Scatter: Scyllo−inositol
MR Estimate
Inverse variance weighted
Weighted median
MR Egger
SNP effect on Multiple Myeloma
2e−04
0e+00
−2e−04
−4e−04
0.02
0.04
0.06
0.08
SNP effect on Scyllo−inositol

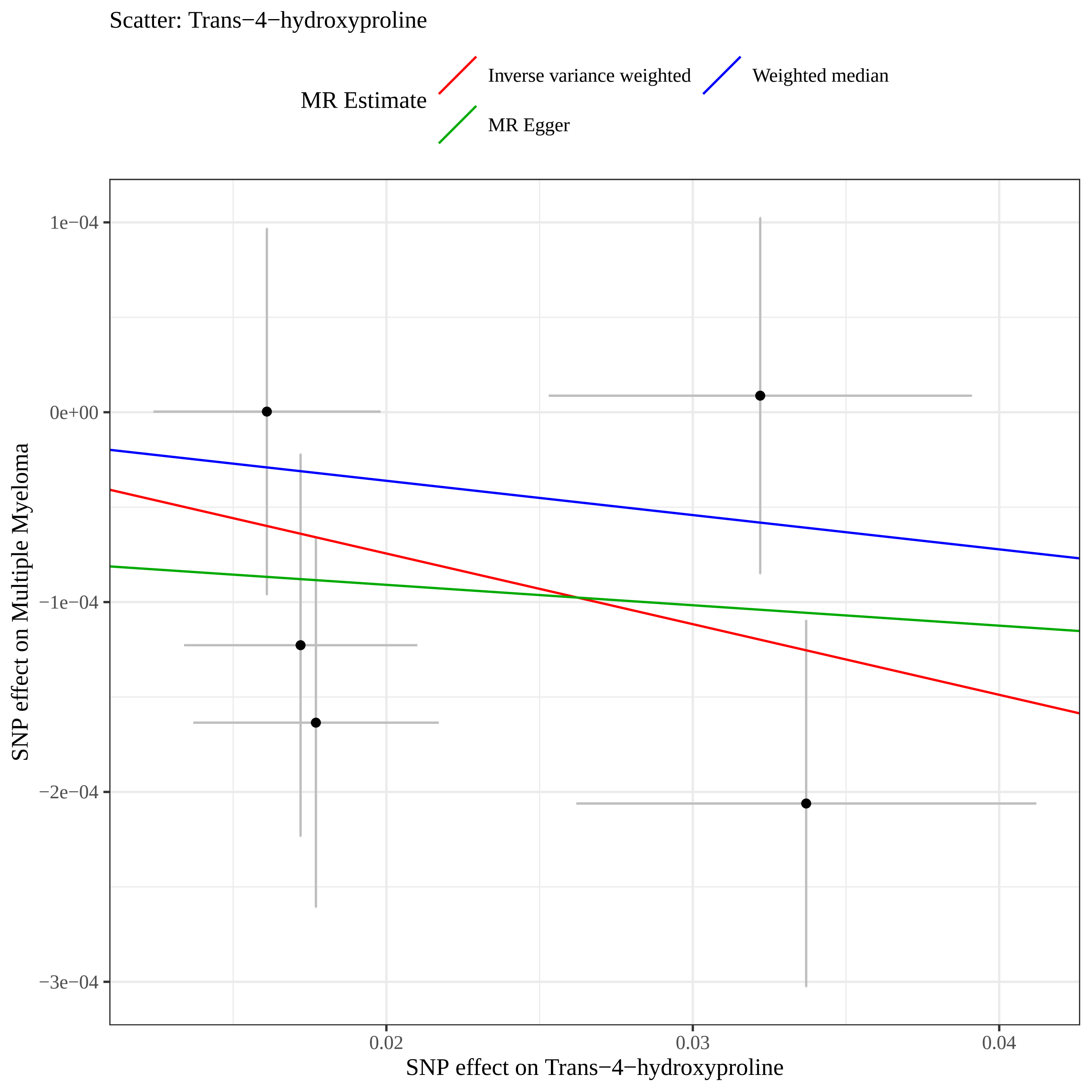
Scatter: Trans−4−hydroxyproline
MR Estimate
Inverse variance weighted
Weighted median
MR Egger
1e−04
0e+00
−1e−04
−2e−04
−3e−04
SNP effect on Multiple Myeloma
0.02
0.03
0.04
SNP effect on Trans−4−hydroxyproline

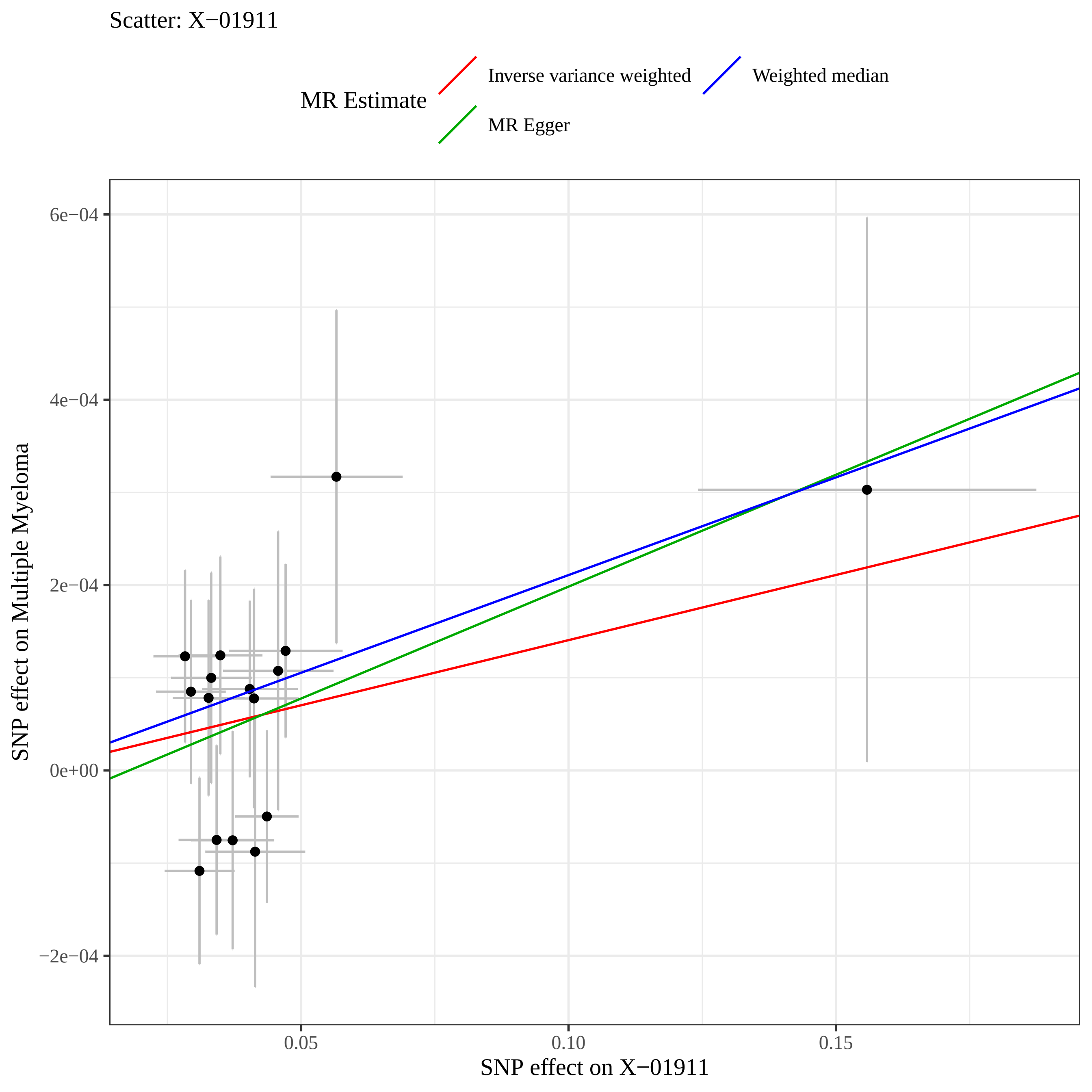
Scatter: X−01911
MR Estimate
Inverse variance weighted
Weighted median
MR Egger
SNP effect on Multiple Myeloma
6e−04
4e−04
2e−04
0e+00
−2e−04
0.05
0.10
0.15
SNP effect on X−01911

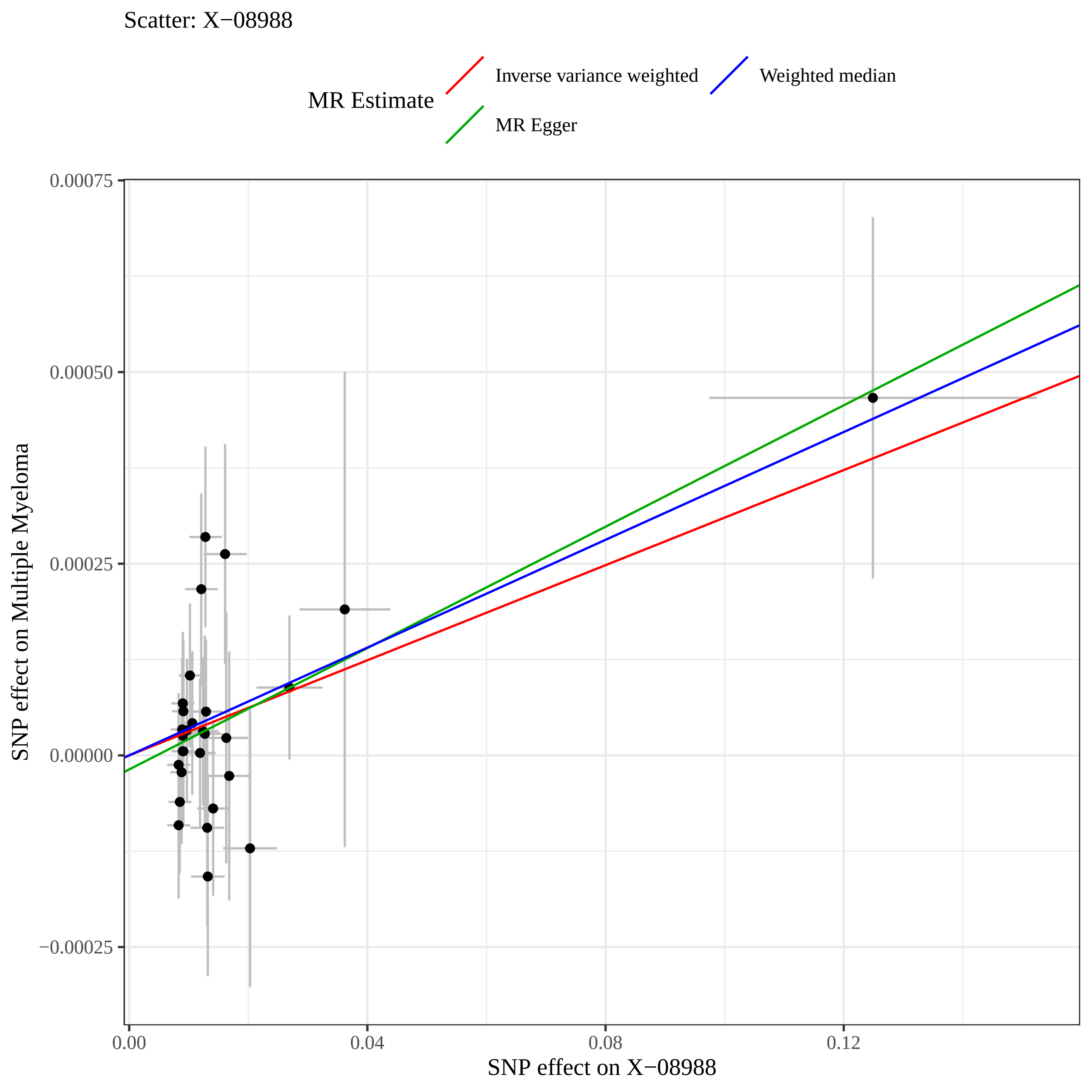

Scatter: X−08988
MR Estimate
Inverse variance weighted
MR Egger
Weighted median
SNP effect on Multiple Myeloma
0.00075
0.00050
0.00025
0.00000
−0.00025
0.00
0.04
0.08
0.12
SNP effect on X−08988

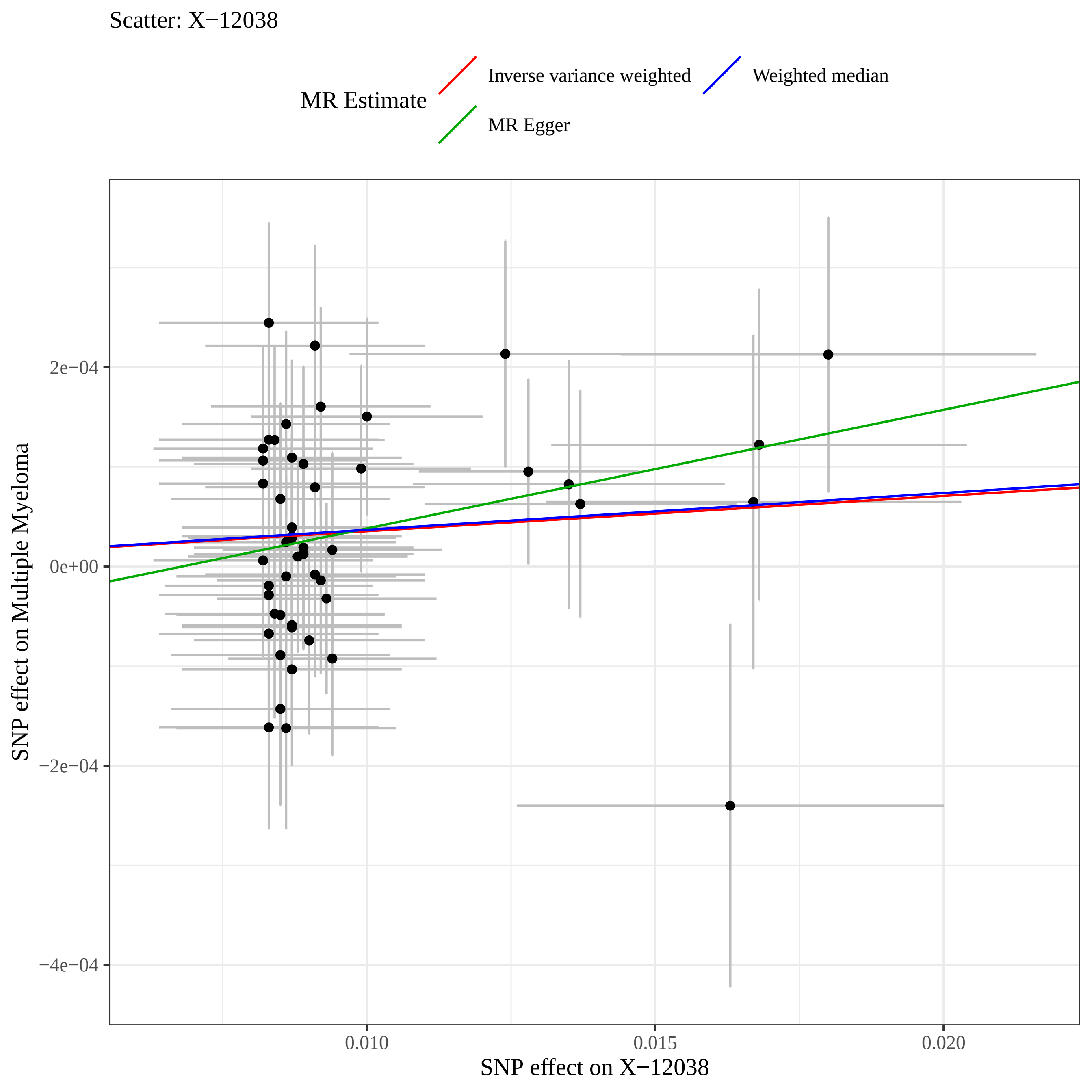

Scatter: X−12038
MR Estimate
Inverse variance weighted
Weighted median
MR Egger
SNP effect on Multiple Myeloma
2e−04
0e+00
−2e−04
−4e−04
0.010
0.015
0.020
SNP effect on X−12038

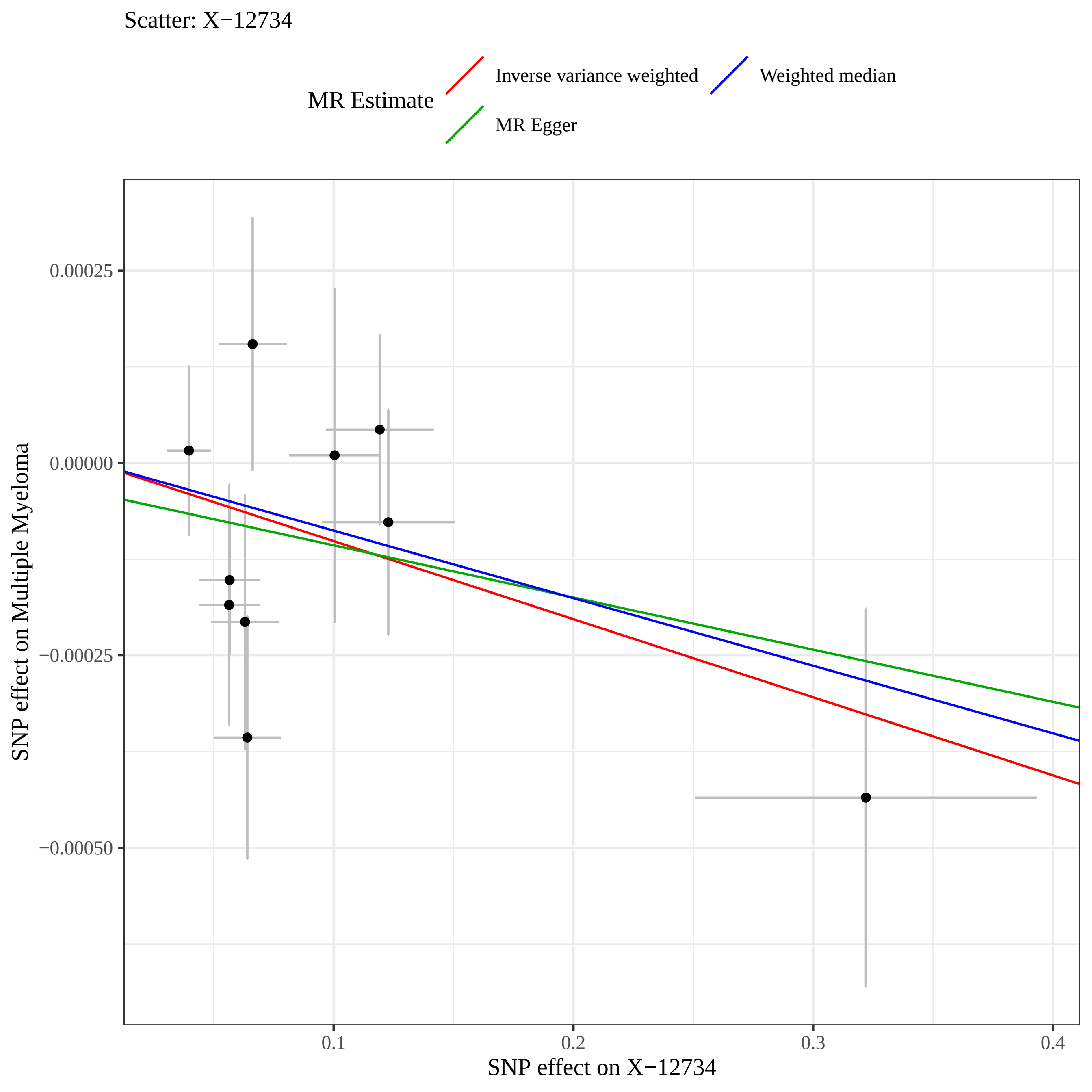
Scatter: X−12734
MR Estimate
Inverse variance weighted
Weighted median
MR Egger
0.00025
0.00000
−0.00025
−0.00050
0.1
0.2
0.3
0.4
SNP effect on Multiple Myeloma
SNP effect on X−12734

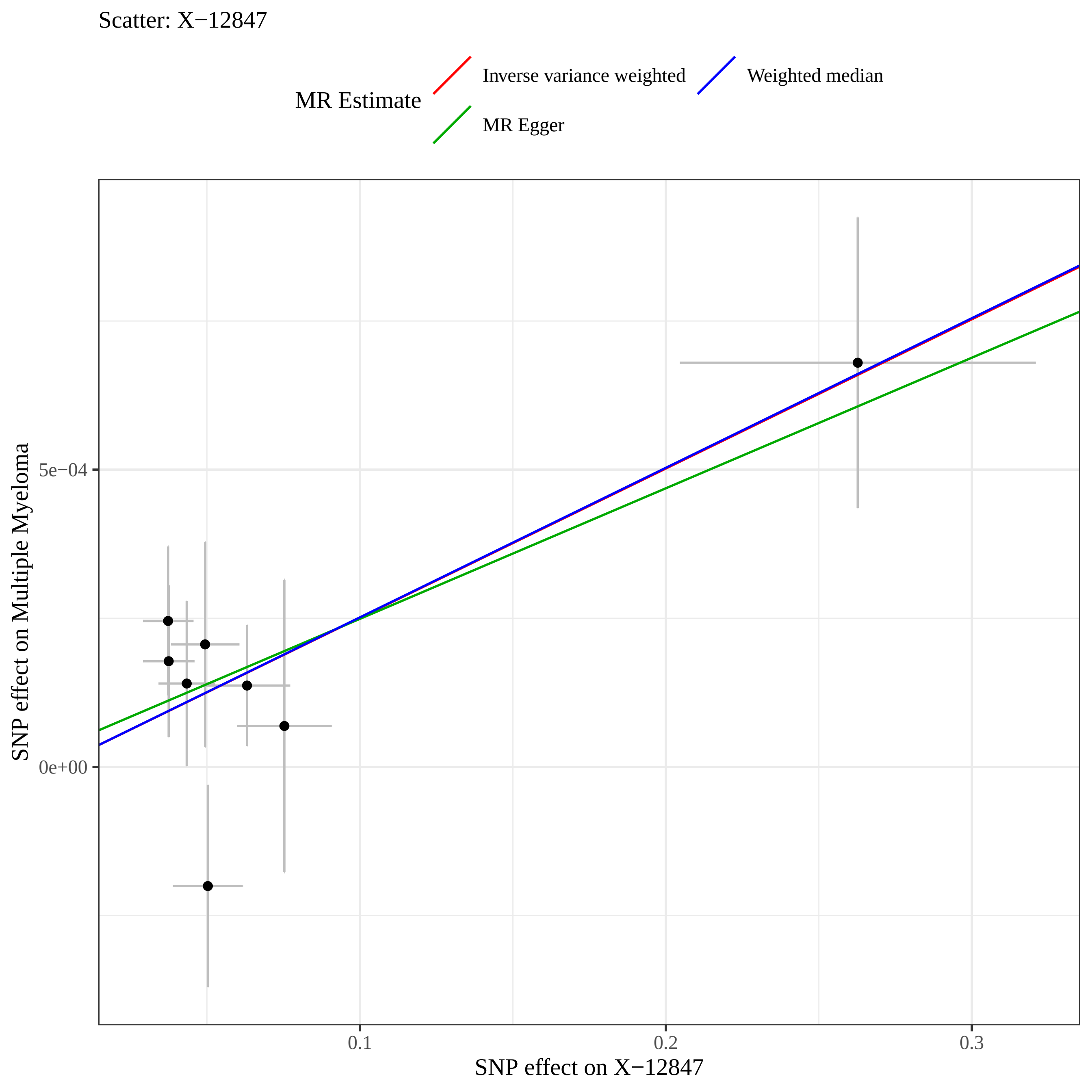

Scatter: X−12847
MR Estimate
Inverse variance weighted
Weighted median
MR Egger
SNP effect on Multiple Myeloma
5e−04
0e+00
0.1
0.2
0.3
SNP effect on X−12847

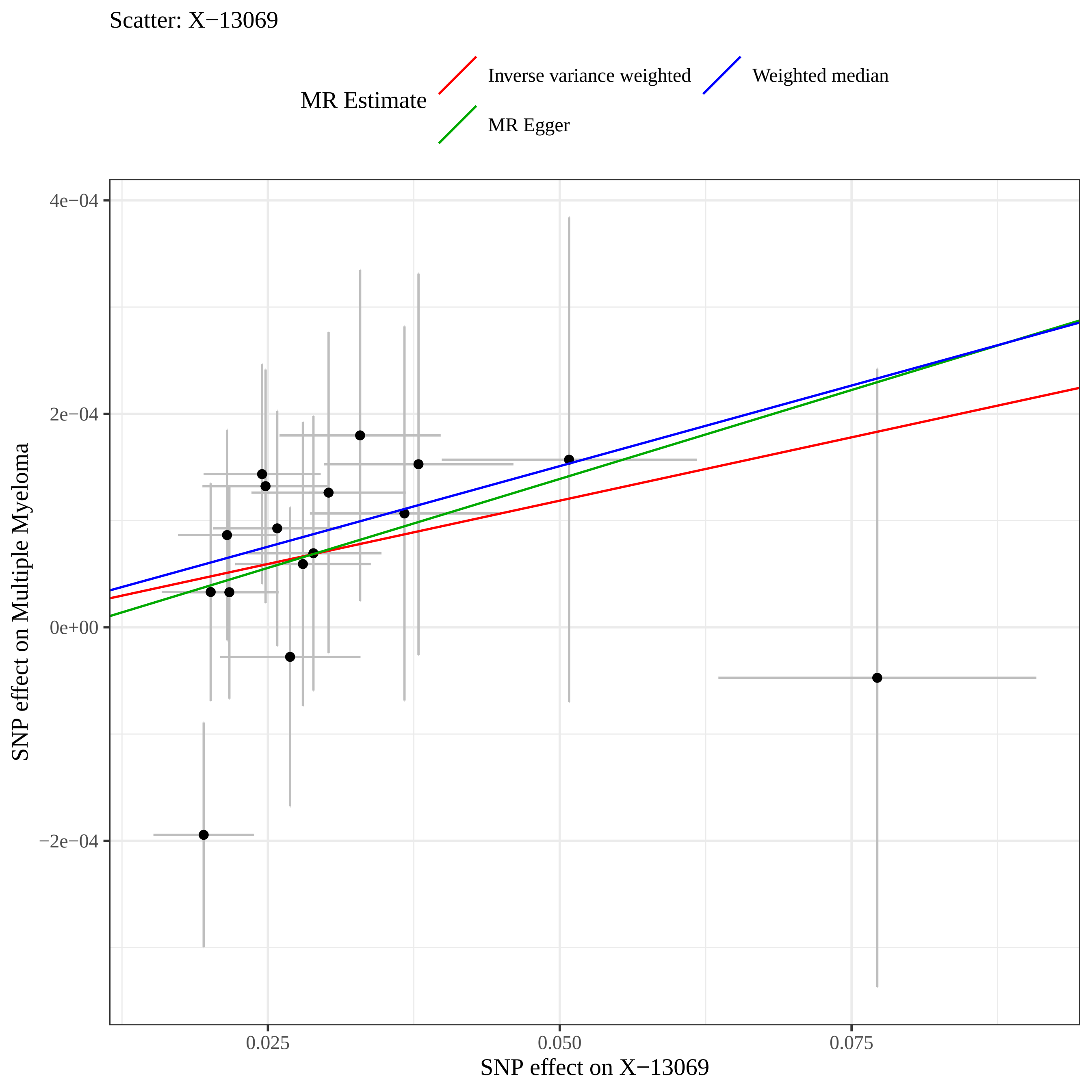

Scatter: X−13069
MR Estimate
Inverse variance weighted
MR Egger
Weighted median
SNP effect on Multiple Myeloma
4e−04
2e−04
0e+00
−2e−04
SNP effect on X−13069
0.025
0.050
0.075

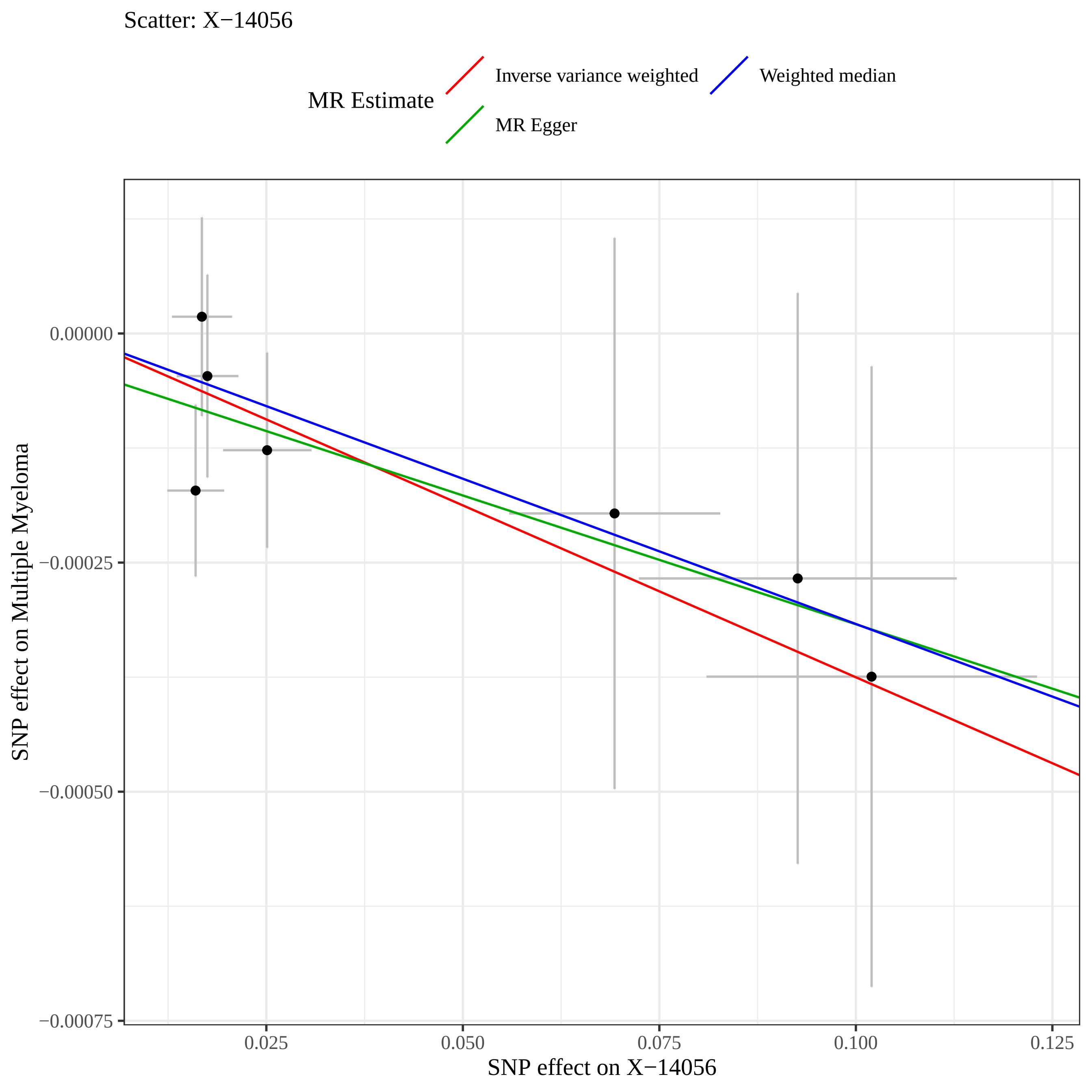
Scatter: X−14056
MR Estimate
Inverse variance weighted
MR Egger
Weighted median
SNP effect on Multiple Myeloma
0.00000
−0.00025
−0.00050
−0.00075
0.025
0.050
0.075
0.100
0.125
SNP effect on X−14056
