# Supplementary material for: Integrating Bidirectional Mendelian Randomization with Multi-Omics Reveals Causal Serum Metabolites and Novel Metabolic Drivers of Multiple Myeloma
Source: Int J Mol Sci. 2026 Feb 16;27(4):1904. doi: 10.3390/ijms27041904 (PMC12941277; doi:10.3390/ijms27041904)
Supplement: Supplementary file 1 [file ijms-27-01904-s001.zip › Table_S1.pdf]

Table S1. Reverse MR analysis of causal effects of MM on significant serum metabolites.

Metabolite	nSNP	Inverse variance weighted		Steiger directionality test	
		Beta	P-value	Correct Direction	P-value
Isoleucine	8	0.054	0.963	FALSE	0.113
Lysine	8	0.920	0.557	FALSE	< 0.001
3-methyl-2-oxovalerate	8	-0.045	0.978	FALSE	0.109
Dihomo-linoleate (20:2n6)	8	-3.288	0.345	FALSE	0.001
1,6-anhydroglucose	8	0.489	0.942	FALSE	0.004
Dimethylarginine (SDMA + ADMA)	8	-3.185	0.016	FALSE	0.001
Trans-4-hydroxyproline	8	2.701	0.438	FALSE	0.002
Scyllo-inositol	8	5.356	0.118	FALSE	0.005
Glutaroyl carnitine	8	-4.685	0.028	FALSE	0.002
10-heptadecenoate (17:1n7)	8	-3.021	0.276	FALSE	0.068
1-docosahexaenoylglycerophosphocholine*	8	-2.113	0.473	FALSE	0.014
N-acetylthreonine	8	-0.636	0.746	FALSE	0.014
1-oleoylglycerophosphocholine	8	2.247	0.304	FALSE	0.011
X-01911	8	0.736	0.889	FALSE	0.019
X-08988	8	-0.126	0.952	FALSE	0.004
X-12038	8	1.333	0.429	FALSE	0.038
X-12734	8	3.592	0.697	FALSE	< 0.001
X-12847	8	0.517	0.936	FALSE	0.001
X-13069	8	-0.719	0.854	FALSE	0.004
X-14056	8	-2.678	0.434	FALSE	0.002
