# Supplementary material for: Integrating Bidirectional Mendelian Randomization with Multi-Omics Reveals Causal Serum Metabolites and Novel Metabolic Drivers of Multiple Myeloma
Source: Int J Mol Sci. 2026 Feb 16;27(4):1904. doi: 10.3390/ijms27041904 (PMC12941277; doi:10.3390/ijms27041904)
Supplement: Supplementary file 1 [file ijms-27-01904-s001.zip › Table_S2.pdf]

Table S2. Metabolic pathway enrichment analysis results for the 21 candidate causal metabolites in relation to MM.

Pathway	Total	Expected	Hits	P-value	FDR	Impact
**Valine, leucine and isoleucine biosynthesis**	8	0.025	1	0.025	1.000	0.000
**Biotin metabolism**	10	0.031	1	0.031	1.000	0.000
**One carbon pool by folate**	26	0.082	1	0.079	1.000	0.050
**Lysine degradation**	30	0.094	1	0.091	1.000	0.000
**Cysteine and methionine metabolism**	33	0.104	1	0.100	1.000	0.105
**Glycerophospholipid metabolism**	36	0.113	1	0.108	1.000	0.017
**Arginine and proline metabolism**	36	0.113	1	0.108	1.000	0.021
**Valine, leucine and isoleucine degradation**	40	0.126	1	0.120	1.000	0.000
